# An Efficient Implementation of the Nwat-MMGBSA Method to Rescore Docking Results in Medium-Throughput Virtual Screenings

**DOI:** 10.3389/fchem.2018.00043

**Published:** 2018-03-05

**Authors:** Irene Maffucci, Xiao Hu, Valentina Fumagalli, Alessandro Contini

**Affiliations:** Dipartimento di Scienze Farmaceutiche, Sezione di Chimica Generale e Organica “Alessandro Marchesini,” Università degli Studi di Milano, Milan, Italy

**Keywords:** MM-GBSA, explicit water, molecular dynamics, GPU, structure based virtual screening, protease, protein-protein interactions

## Abstract

Nwat-MMGBSA is a variant of MM-PB/GBSA based on the inclusion of a number of explicit water molecules that are the closest to the ligand in each frame of a molecular dynamics trajectory. This method demonstrated improved correlations between calculated and experimental binding energies in both protein-protein interactions and ligand-receptor complexes, in comparison to the standard MM-GBSA. A protocol optimization, aimed to maximize efficacy and efficiency, is discussed here considering penicillopepsin, HIV1-protease, and BCL-XL as test cases. Calculations were performed in triplicates on both classic HPC environments and on standard workstations equipped by a GPU card, evidencing no statistical differences in the results. No relevant differences in correlation to experiments were also observed when performing Nwat-MMGBSA calculations on 4 or 1 ns long trajectories. A fully automatic workflow for structure-based virtual screening, performing from library set-up to docking and Nwat-MMGBSA rescoring, has then been developed. The protocol has been tested against no rescoring or standard MM-GBSA rescoring within a retrospective virtual screening of inhibitors of AmpC β-lactamase and of the Rac1-Tiam1 protein-protein interaction. In both cases, Nwat-MMGBSA rescoring provided a statistically significant increase in the ROC AUCs of between 20 and 30%, compared to docking scoring or to standard MM-GBSA rescoring.

## Introduction

Structure based virtual screening (SBVS) methods are widely applied in drug discovery (Enyedy and Egan, 2008; Sousa et al., 2013). In most of the cases, SBVSs are done in the hit-to-lead development phase of the drug discovery process, with multiple successful outcomes (Enyedy et al., 2001a,b; Vangrevelinghe et al., 2003). In SBVS-related studies, scoring functions are mostly applied for potential hit selection. In general, the scoring functions are based on either empirical, knowledge-based, or molecular mechanics force field derived potentials (Wang et al., 2003; Raha et al., 2007). Additionally, to make the virtual screening process computational inexpensive, the scoring functions are most likely simplified. Thus, some important contributions known to influence the binding affinity are neglected (Sousa et al., 2006; Moitessier et al., 2008). Inevitably, applications of such simplified methods tend to fail in the hit optimization phase, where more meticulous selections are required about structurally similar compounds for better prediction of biological activity (Leach et al., 2006; Tirado-Rives and Jorgensen, 2006; Warren et al., 2006; Enyedy and Egan, 2008).

A better scoring can be achieved by considering energy evaluation averaged over an ensemble of conformations from a complex dynamic trajectory, as is the underlying concept of the molecular mechanics Poisson-Boltzmann / Generalized Born surface area (MM-PB/GBSA) analysis. Of course, the applications of MM-PB/GBSA methods are at the cost of increased computational expenses (Massova and Kollman, 2000). Nonetheless, the MM-PB/GBSA methods have been successfully applied to estimate binding energies (Kollman et al., 2000), or incorporated as a scoring method in SBVS applications (Lyne et al., 2006; Zhou et al., 2006; Ferrari et al., 2007; Xiong et al., 2007; Xu et al., 2010; Xu, 2012; Knight et al., 2014). The treatment of the solvent in MM-PB/GBSA calculations is implicit, providing an acceptable estimations of the energy contribution while bulk water is the only solvent-related concern (Wong and Lightstone, 2011; Yang et al., 2013). However, explicit water molecules might also be important in forming biomolecular complexes (Chong and Ham, 2017), particularly waters involved in bridging the ligand and the receptor (Wong et al., 2009; Abel et al., 2011; Ahmad et al., 2011; Wallnoefer et al., 2011; Maffucci and Contini, 2013; Mikulskis et al., 2014). Indeed, by analyzing several thousand of crystallographic complexes, it was recently observed that at least a water molecule mediates contacts between the partners in about two thirds of all the considered systems (Hendlich et al., 2003). Thus, several computational methods were proposed to aid the identification of important water molecules in crystal structures (Raymer et al., 1997; García-Sosa et al., 2003; Amadasi et al., 2006). Moreover, although replacing a water molecule in the binding site is a generally accepted strategy to increase drug potency, it has been shown that better pharmacodynamic properties might be obtained by keeping a tightly bound water as a bridge between the ligand and the receptor (García-Sosa, 2013). The effects of targeting or displacing binding site waters in drug design can be rigorously assessed by free energy calculations (García-Sosa and Mancera, 2010), that however are still too demanding when libraries of hundreds of molecules need to be evaluated. Therefore, some approaches have been attempted to consider the contribution of water-mediated interactions into the ligand docking score (Young et al., 2007; Ricchiuto et al., 2008; Forli and Olson, 2012; Ross et al., 2012; Kumar and Zhang, 2013; Murphy et al., 2016) or into the MM-PB/GBSA estimated binding energy (Checa et al., 1997; Wong et al., 2009; Genheden et al., 2011; Wallnoefer et al., 2011; Greenidge et al., 2013; Maffucci and Contini, 2013).

In this framework, we developed a MM-PB/GBSA variant, that we refer as Nwat-MMGBSA, which provided good-to-excellent results in ranking the binding energies of different protein-ligand or protein-protein complexes (Maffucci and Contini, 2013, 2016). Nwat-MMGBSA is based on the inclusion of a number of explicit water molecules, that are selected to be the closest to the ligand in each frame of the MD trajectory and are included as part of the receptor during the analysis. In addition to our work (Maffucci and Contini, 2013, 2016), Aldeghi and coworkers recently validated, by a thorough statistical analysis, the use of this approach on bromodomains (Aldeghi et al., 2017). Compared to other methods that include explicit water in MM-PB/GBSA calculations, Nwat-MMGBSA might have some advantages. For instance, relevant explicit water might be selected from the crystal structure (Wong et al., 2009; Wallnoefer et al., 2011). However, this imply that high resolution crystal structures are available, while Nwat-MMGBSA calculations can be performed on receptor models obtained by other techniques, such as homology modeling or NMR. Moreover, crystallographic water sites might derive from the average electron density of several molecules competing for the same position (Schiffer and Hermans, 2003). Indeed, we previously observed that a water-bridge between the ligand and the receptor found in the crystal structure of topoisomerase I in complex with topotecan (Staker et al., 2002) was described by the competition of three different waters in a 4 ns MD trajectory (Maffucci and Contini, 2013). It was also reported that explicit water for MM-GB/PBSA calculations might be selected from MD simulations accordingly to their distance from the ligand (Zhu et al., 2014). In this case, the distance from the ligand atoms is fixed, while the number of waters is different in each snapshot selected for MM-PB/GBSA analysis. However, by comparing this method to Nwat-MMGBSA, where the number of selected water is constant among all snapshots, we observed that Nwat-MMGBSA provided a better correlation with experiments and a better reproducibility among multiple repetitions of the same calculation (Maffucci and Contini, 2016). In this work, aiming to make Nwat-MMGBSA suitable for rescoring ligands in low- to medium-throughput SBVS experiments, we optimized the protocol to improve its efficiency, without losing in accuracy. We selected penicillopepsin (James et al., 1992; Ding et al., 1998; Hou et al., 2011a), HIV1-protease mutants (Shen et al., 2010; Olajuyigbe et al., 2011) and BCL-X_L_ (Lessene et al., 2013) as test systems with known experimental data, either binding free energy (Δ*G*), inhibition constant (*k*_*i*_), or IC_50_. Our studies have shown improvements in the coefficient of determination to experimental data (*r*^2^) ranging from 10 to 60%, depending on the number of explicit water molecules considered in the energy evaluation. Moreover, we assessed the Nwat-MMGBSA approach for SBVS rescoring performance in a ligand-protein interaction and a protein-protein interaction (PPI) scenario (AmpC β-lactamase and Rac1-Tiam1, respectively). In both cases, improved outcomes were observed compared to either docking scoring or to standard MM-GBSA rescoring. Furthermore, the complete SBVS workflow applied in this work, including Nwat-MMGBSA rescoring, is provided in the Supplementary Information as a set of bash and tcsh scripts that, together with working tutorials, should make it readily applicable to other biomolecular systems of interest.

## Methods

### Preparation of complexes

Crystal structures of the penicillopepsin [PDB codes: 1APU, 1APV, 1APT, 1APW (James et al., 1992), 2WEA, 2WEB, and 2WEC (Ding et al., 1998)] and HIV1-protease [PDB codes: 3NU3, 3NU4, 3NU5, 3NU6, 3NUJ, 3NU9, 3NUO (Shen et al., 2010), 3NDW, and 3NDX (Olajuyigbe et al., 2011)] complexes (Table S1) were obtained from RCSB Protein Databank (Figures S1, S2). However, for the BCL-X_L_ system, (Figure S3) only 3ZK6, 3ZLN, 3ZLO, and 3ZLR complexes were available as crystal structures (Lessene et al., 2013). Therefore, the starting structures of the unavailable complexes were reconstructed using MOE software (Molecular Operating Environment, v2016.08, 2016) starting from the available ones. Ligand partial charges were derived with the AM1-BCC method using the *antechamber* (Wang et al., 2006) software of AmberTools15 package (Case et al., 2014). All waters, ions and stabilizing agents present in the crystal structures were removed. The protonation state of every titratable residue within the complexes were assigned at physiological conditions using the Protonate-3D module of MOE.

### MD simulations

MD simulations were performed with the *pmemd.MPI* or *pmemd.cuda* (Götz et al., 2012; Salomon-Ferrer et al., 2013) modules, depending on the hardware (classical HPC environment or GPU equipped workstations, respectively), included in the Amber14 package (Case et al., 2014). The ff14SB (Maier et al., 2015) and the gaff (Wang et al., 2004) force fields were adopted for the protein and the ligand in all simulations respectively. In each complex, the total charge was neutralized by adding Na+ or Cl- ions, and the systems were solvated by an octahedral box of TIP3P water (Jorgensen et al., 1983), with a box size of 10 Å from the solute.

The equilibration and production protocols were updated to optimize performance, in respect to previous studies (Maffucci and Contini, 2013, 2016). The systems were initially relaxed by optimizing the position of hydrogens (1,000 cycles of steepest descent (SD) and 5,000 cycles of conjugated gradient (CG), up to a gradient of 0.01 kcal mol^−1^ · Å; restraints of 100 kcal·mol^−1^ · Å^2^ were applied on heavy atoms) and of ions and waters (2,000 cycles of SD and 5000 cycles of CG up to a gradient of 0.1 kcal·mol^−1^·Å; restraints of 50 kcal·mol^−1^·Å^2^ were applied on atoms other than ions and water). The solvent box was then equilibrated at 300 K by 100 ps of NVT and 100 ps of NPT simulation using a Langevin thermostat with a collision frequency of 2.0 ps^−1^ (restraints of 50 and 25 kcal·mol^−1^·Å^2^ were applied on the solute for NVT and NPT simulations, respectively). Successively, two cycles of restrained minimization (2500 cycles of steepest descent and 5,000 cycles of conjugated gradient, up to a gradient of 0.1 kcal mol^−1^ Å, with restraints of 25 and 10 kcal mol^−1^ Å^2^ on backbone atoms, respectively) were performed. The systems were then heated up to 300 K in 6 steps (ΔT = 50 K) of 5 ps each, where backbone restraints were gradually reduced from 10.0 to 5.0 kcal mol^−1^ Å^2^. An equilibration of 1.6 ns was then performed by initially using the NVT ensemble (100 ps, ligand and backbone restraints = 5.0 kcal mol^−1^ Å^2^) followed by NPT (1 step of 200 ps with ligand and backbone restraints = 5 kcal mol^−1^ Å^2^, then 3 steps of 100 ps each reducing the ligand and backbone restraints from 5.0 to 1.0 kcal mol^−1^ Å^2^, and finally 1 step of 500 ns with ligand and backbone restraints of 1.0 kcal mol^−1^ Å^2^). The last equilibration step consisted in 500 ps of unrestrained NVT simulation. Finally, production runs were conducted under the NVT condition at 300 K for 1 or 4 ns. An electrostatic cutoff of 8.0 Å, PME (Darden et al., 1993) for long electrostatic interactions, and the SHAKE (Ryckaert et al., 1977) algorithm were applied to all the calculations. Three independent simulations were performed for each hardware set-up (GPU workstation or CPU HPC cluster). For the simulations performed on GPUs, the default single precision/fixed precision (SPFP) version of *pmemd.cuda* (Le Grand et al., 2013) was applied in all steps, except for geometry minimizations where the double precision/fixed precision (DPFP) version was adopted.

All MD production trajectories were processed by *cpptraj* for backbone RMSD analyses (Figures S4–S11), solute-solvent hydrogen bond (donor-acceptor distance cutoff at 4.0 Å, angle cutoff at 150°) and water density (grid analysis over a cubic box 50 Å × 50 Å × 50 Å, mesh = 0.5 Å, centered on ligands) analyses. Images of water density plots were obtained by using UCSF Chimera (Pettersen et al., 2004).

### Nwat-MMGBSA analyses

MM-GBSA and Nwat-MMGBSA analyses were performed with the *MMPBSA.py* script (Miller et al., 2012) of the AmberTools15 package. The analyses were conducted on either the 1st or the 4th ns of the production runs by selecting 100 frames evenly spaced out. The GB-Neck2 implicit solvent model (Nguyen et al., 2013) was chosen for the GB calculations and the salt molar concentration in solution was set at 0.15 M. Entropy was neglected in all calculations, since the benefits of including its contribution still remain controversial (Weis et al., 2006; Hou et al., 2011a; Wallnoefer et al., 2011; Yang et al., 2011) and normal mode calculations are also extremely time consuming. It should be noted that neglecting entropy, although acceptable when comparing ligands of similar size and structure (Kollman et al., 2000; Wang et al., 2001; Wong et al., 2009), might lead to errors when the analysis involves ligands that are structurally rather different (Oehme et al., 2012).

**Figure 1:**
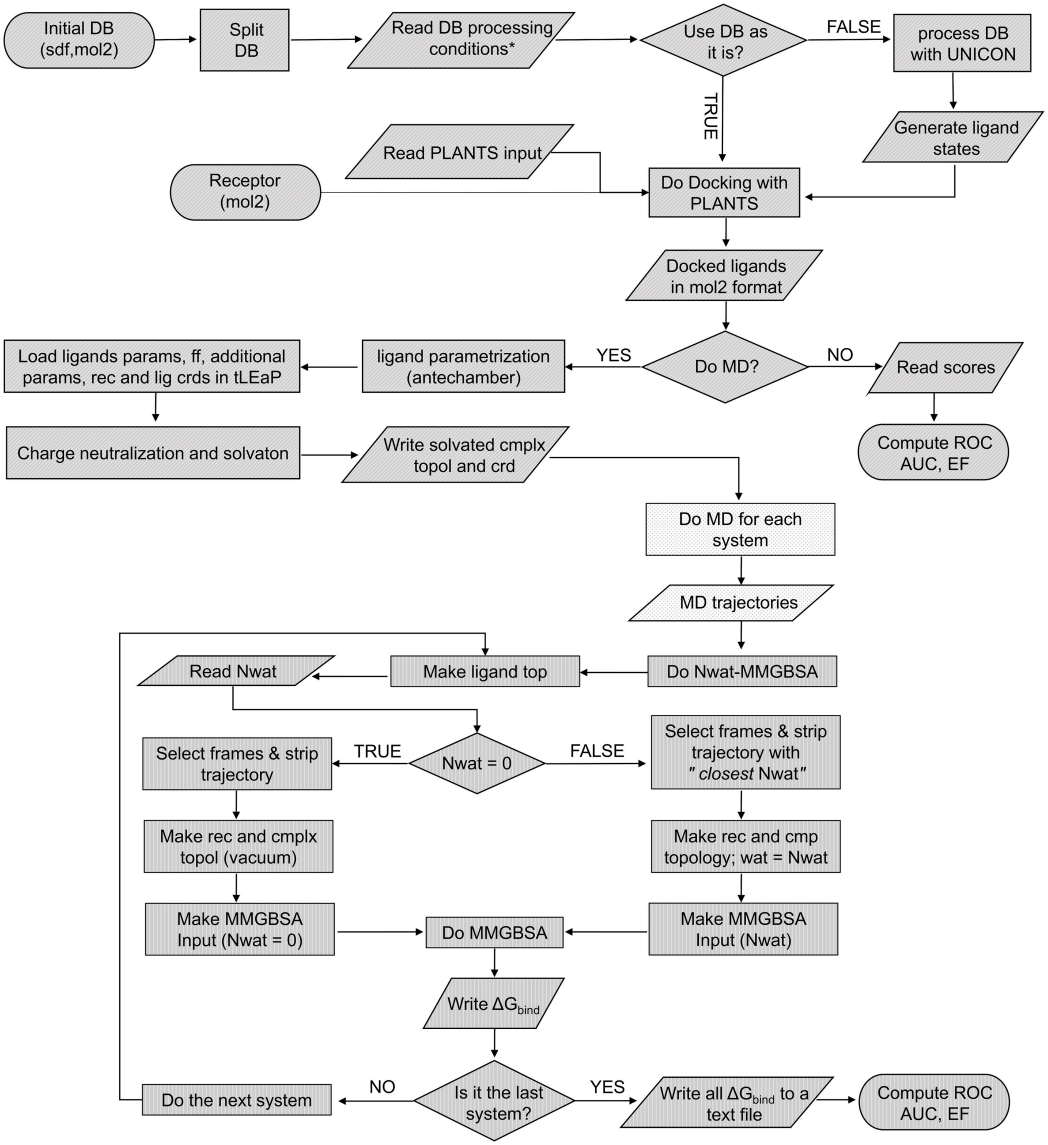
Pseudocode of the complete screening and rescoring workflow. Each texture represents an independent script: VScreen (diagonal), autoMD (dots), and Nwat-MMGBSA (vertical).

The *Nwat-MMGBSA* script (Figure 1) uses the *cpptraj* module of AmberTools15 to process the solvated MD trajectory. When Nwat > 0, the water molecules closest to the ligand were preserved while the remaining were stripped from the selected frames by using the *cpptraj* command *closest*. The total number of water molecules to be kept in the trajectory is given by the *Nwat* flag in the script input section (in this work, we evaluated Nwat = 10, 20, 30, 40, 50, 60, 70, 80, 90, or 100). The number of frames that are going to be selected from the original MD trajectory is defined by the *r* flag in the script input section, that corresponds to the *interval* keyword in the *MMPBSA.py* script (Miller et al., 2012). In this work, *r* was set at 10, meaning that one every 10 frames (i.e., 100 frames per nanosecond) was sampled. The preserved closest water molecules are considered as part of the receptor during the MM-GBSA analysis. In analogy with studies on the MM-PB/GBSA performance previously reported by us and by others (Hou et al., 2011b; Maffucci and Contini, 2013, 2016; Xu et al., 2013), the coefficient of determination (*r*^2^) between experimental data and calculated binding energies was used as the evaluation metric.

### Restrospective virtual screening

#### Preparation of the receptor

The AmpC β-lactamase receptor was derived from the 2HDS PDB file (Babaoglu and Shoichet, 2006) according to what described on the DUD-E website (Mysinger et al., 2012). Starting from the crystal structure, only chain B was preserved, crystallographic water molecules were removed and the “Structure preparation” module of the MOE software was used to check the protein structure and correct eventual errors. The receptor was then capped by acetyl (ACE) and methylamino (NME) groups at the N- and C-termini, respectively. Missing hydrogen atoms were added using the “Protonate 3D” function of the MOE package, considering a physiological pH. Partial charges were added accordingly to the AMBER10:EHT force field and solvation was treated with the Born model. The system geometry was then optimized up to a gradient of 0.1 kcal mol^−1^ Å, with protein backbone atoms restrained to the original position.

The Rac1 receptor, used in the VS simulation, was prepared as described elsewhere (Ferri et al., 2009, 2013a; Ruffoni et al., 2014).

#### AmpC β-lactamase testing library

The DUD-E database provides 48 experimentally determined active ligands and 2850 decoy molecules for AmpC β-lactamase. However, considering the computational cost of rescoring by MD simulations followed by Nwat-MMGBSA analyses, we considered using a smaller library that decently represent the original database. Hence, fingerprint clustering methods included in MOE package were applied to reduce the size of the test set. Multiple fingerprint/similarity metric method combinations have been trial-and-error-ed. We found that the application of Typed Graph Triangle (TGT) fingerprint and Tanimoto Superset/Subset (tanimoto-ss) similarity metric method provides the closest reproduction of virtual screening results as the data provided by DUD-E. However, the combination applied here might not be directly transferred to other biomolecular systems. The clustering process reduced the original database to 20 active ligands and 378 decoys (Table S2) with a docking AUC at 74.82% and top 1% enrichment factor of 9.5, in comparison to the original 78.92% and 8.3 provided on DUD-E database. The smiles of the final database are reported in Table S2.

#### Rac1 testing library

By analyzing the literature and by using in-house data, we collected a set of 116 compounds, 10 of which were active and 106 inactive on the Rac1 protein (Table S3). The active compounds were selected among those able to inhibit at least the 50% of Rac1 activity, as assessed by G-LISA biochemical assays (Ferri et al., 2009, 2013a). Conversely, the decoys were chosen among molecules that were designed (or identified by computational screening) as Rac1 inhibitors, but turned out to be completely inactive on experimental evaluation (Ferri et al., 2009, 2013a; Hernández et al., 2010; Surviladze et al., 2010; Shang et al., 2012; Rahimi et al., 2015; Lu et al., 2017). The selected compounds were designed with MOE, minimized and subjected to a conformational search (MMFF94x force field, Born solvation, with the other parameters as default). The lowest energy conformation of each compound was selected to form the final test set.

#### Virtual screening

The workflow included in the *VScreen* script (see Supplementary Information) allows the following combinations for library processing:

1. Use the library as it is
2. Generate tautomers
3. Generate stereoisomers and tautomers
4. Generate ring conformations and tautomers
5. Generate stereoisomers, ring conformations, and tautomers
6. Generate stereoisomers, ring conformations, tautomers, and protonation states.

The sixth library processing tandem was applied in this work. The UNICON software is used to generate tautomer and protonation states (Sommer et al., 2016). We chose the *topscoring* keyword to generate only the most favored tautomers and protomers, as in preliminary evaluation we observed that the generation of all tautomers and protomers (using the *ensemble* keyword) did not provide improved results and was significantly more time consuming. The SPORES software (ten Brink and Exner, 2009, 2010) is instead used to obtain stereoisomer and ring conformation, as well as for the final assignment of atom types, as requested by the PLANTS software (Korb et al., 2006, 2007, 2009, 2010) used for all dockings. Specific docking parameters, including search speed and scoring functions, can be set directly in the *VScreen* script. In the examples reported here, PLANTS was used in a low speed / high accuracy mode (search speed = *speed1*) and with the *CHEMPLP* scoring function (Korb et al., 2009). Additional PLANTS commands, such as H-bond or NMR constraints (Korb et al., 2010), can also be inserted in the input section of the *VScreen* script. Concerning the Rac1 example, we requested a H-bond constrain of 3 kcal/mol between any H-bond donor of the docking ligand and the carbonyl oxygen of Leu 70, since the literature evidence the importance of such ligand-receptor interaction for a proper activity (Montalvo-Ortiz et al., 2012; Ferri et al., 2013a; Ruffoni et al., 2014). Binding site radii were optimized to 16 Å for Rac1 and 7 Å for AmpC β-lactamase test sets, respectively. After the virtual screening process, the outcomes were ranked according to total PLANTS_CHEMPLP_ score, using the top ranked pose of each ligand. Receiver operating characteristic (ROC) curves and corresponding area under curve (AUC) were then generated at the end of each docking run by using an R script integrated in the *VScreen* program.

#### Ligand parameterization

Following the docking, automatized parametrization of ligands for later MD simulations can be enabled by setting the *doMD* keyword to 1. The user is allowed to choose a “top percentage” of the ranked ligands to be subjected to parametrization, by setting the *fract* keyword. We have chosen 100% (*fract* = 100), *i.e.*, the full test set, as we were interested in a full assessment of the Nwat-MMGBSA methods in terms of virtual screening rescoring. The *antechamber* software (Wang et al., 2006) of the AmberTools15 package is used for deriving AM1-BCC partial charges for each ligand and to assign atom types accordingly to the *gaff* force field (Jakalian et al., 2002; Wang et al., 2004). The quantum mechanical calculations necessary to perform the charge parameterization can be accomplished by using the default *sqm* software included in AmberTools15, or with MOPAC2016 (Stewart, 2016) by setting the *qm* keyword to 2 or 0, respectively. The topology and starting coordinate files of each complex are then generated by calling the *tleap* software, included in the AmberTools15 package. Each complex is neutralized and solvated by adding Na^+^ or Cl^−^ ions and a TIP3P water box of 10 Å from the solute. MD simulations and Nwat-MMGBSA analyses can then be performed as detailed in previous sections.

Nwat-MMGBSA analyses were performed on the obtained trajectories with number of closest water molecules set to 0, 30, 60, or 100. ROC curves and corresponding AUCs were evaluated by using the rankings derived from each Nwat-MMGBSA analysis.

All scripts applied in this work are available in the Supplement Materials. Eventual updates might also be requested to the authors.

## Results

### Optimization of the Nwat-MMGBSA protocol

To optimize the Nwat-MMGBSA protocol for low- or medium-throughput virtual screening procedures, such as those applied in the hit-to-lead optimization phase of a drug discovery process, we worked on a significant reduction of the overall simulation time in comparison to our previous implementations (Maffucci and Contini, 2013, 2016). Then, we integrated Nwat-MMGBSA in a continuous workflow that includes the library setup, docking and the preparation of complexes that is propaedeutic to MD, as shown in Figure 1. The following steps of the protocol were redesigned for an optimal ratio between accuracy and speed:

- The application of AM1-BBC charges to reduce the computational cost for ligand parameterizations. Indeed, it has been reported that AM1-BCC charges behaved fairly well in MM-PB/GBSA calculations, compared to more sophisticated methods (Xu et al., 2013; Sun et al., 2014).
- The use of the NVT ensemble instead of NPT for last equilibration step and production run. This allowed a 30% reduction on the overall MD simulation time, without a significant variation in the results.

Generalized Born (GB) implicit solvent model is used by default in Nwat-MMGBSA calculations. Indeed, several articles report that GB can provide outcomes comparable to the PB method, at a fraction of the computational cost, especially when relatively short MD trajectories are used for MM-PB/GBSA calculations (Hou et al., 2011a,b; Maffucci and Contini, 2013, 2015, 2016). However, the PB method can still be requested by the user by setting the *solv* keyword in the input section of the Nwat-MMGBSA script (see scripts and examples provided as Supplementary Information).

Moreover, the reproducibility between independent MD simulation repeats of the same system, especially when using GPU, was also improved. This required some protocol adjustments, including a longer equilibration of the solvent box, the use of geometric restraints instead of constraints, the use of the Langevin thermostat instead of the weak coupling algorithm, and a slightly extended final equilibration phase.

The protocol modifications allowed approximately 1.5 h per ligand on a standard workstation equipped with a single GeForce GTX TITAN Black card, including parameterization, minimization, equilibration, 1 ns of production run and Nwat-MMGBSA analysis. This is roughly the same time required for the simulation on a HPC architecture using 12 nodes equipped with two 2.40 GHz octa-core processors under similar simulation settings.

Considering our interest in using Nwat-MMGBSA calculations to rescore docking results in a reasonable time, the following tests were also designed to evidence any statistical difference in the correlation to experiment when the analysis is performed on 1 ns or 4 ns long MD trajectories. All the energies computed for the discussed examples are reported in Tables S4–S39. Correlations to experiments and statistical analyses are shown in Tables S40–S42 and Table S43, respectively.

#### Test on penicillopepsin

**Figure 2:**
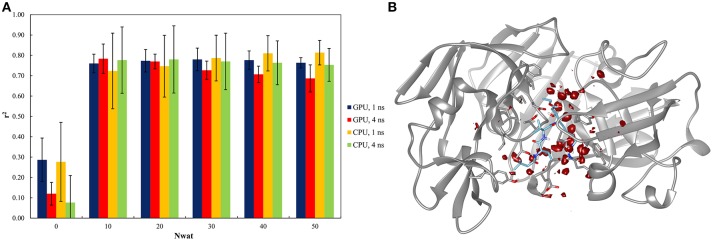
Results of the penicillopepsin system regarding the application of Nwat-MMGBSA method. **(A)** Bar chart of *r*^2^ in dependency at different Nwat and computational conditions. Nwat = 0 corresponds to a standard MM-GBSA calculation. **(B)** Water density plot obtained by grid analysis of penicillopepsin-1APT complex (visualization with Chimera, step = 1, level = 15).

This system was already evaluated, although with a different protocol, in a previous work where the bases of the Nwat-MMGBSA approach were described (Maffucci and Contini, 2013). The results of the Nwat-MMGBSA analysis obtained with the new protocol agreed with those reported in the previous work in terms of correlation between predicted and experimental binding energies (Figure 2A and Table S40). This confirms the robustness of the Nwat-MMGBSA method toward the modifications in the MD simulation conditions. However, the new protocol showed the beneficiary application of Nwat-MMGBSA method even when only 10 closest water molecules were considered (Nwat = 10), while in the previous evaluation no significant improvement was observed at this condition. Water density plot around the binding site (Figure 2B) confirm the role of water in mediating the ligand-receptor binding. Indeed, for this system, the use of the Nwat-MMGBSA methods allowed to increase the *r*^2^ from about 0.3, obtained with the standard MM-GBSA approach (Nwat = 0), to about 0.8 (Figure 2A).

In addition, relatively low standard deviations, obtained when averaging the *r*^2^ obtained by independent repetitions of the whole run, were observed when higher numbers of closest water molecules were considered (Table S40 and Figure 2A), thus suggesting that the inclusion of explicit waters is likely to improve the reproducibility of results from individual runs.

Moreover, the outcome obtained by running simulations on GPU and CPU hardware were statistically equivalent (Table S43), and the same was true for the analyses performed on either the 1st or the 4th ns of MD simulations (Figure 2A). This suggests that Nwat-MMGBSA analysis is suitable for the analysis of short MD simulations run on GPU cards, with a great improvement in speed and no impairments in accuracy.

#### Test on HIV1-protease

**Figure 3:**
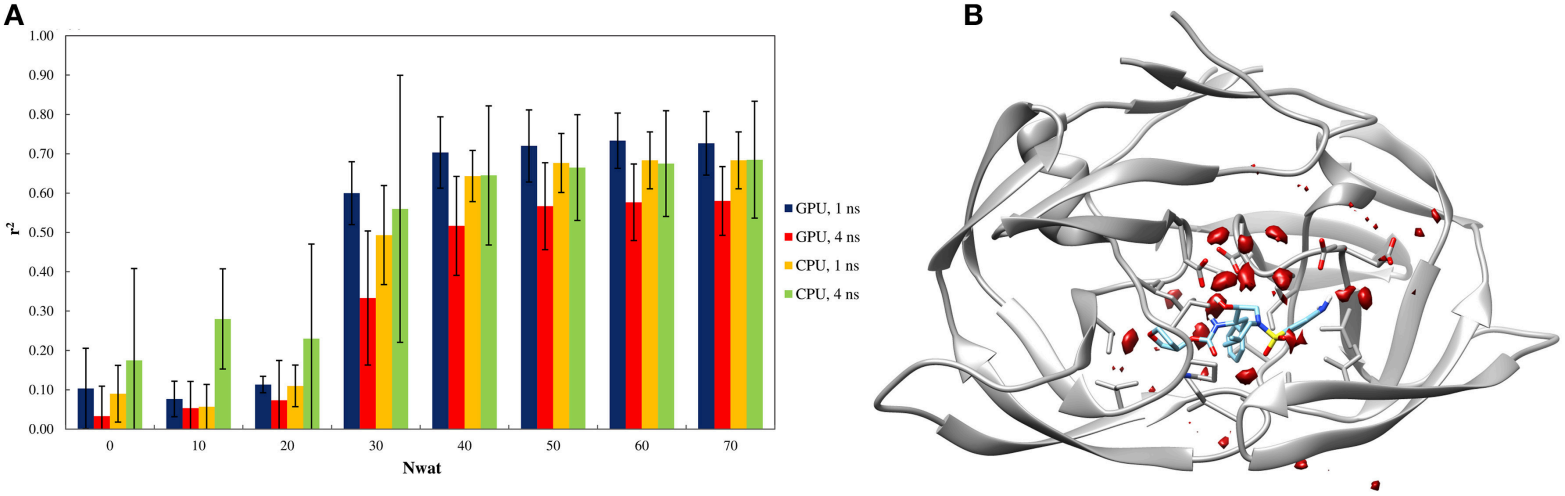
**(A)** Trend of *r*^2^ in dependency of Nwat for HIV1-protease. Nwat = 0 corresponds to a standard MM-GBSA calculation. **(B)** Water density plot obtained by grid analysis of HIV1-protease-3NUO complex (visualization with Chimera, step = 1, level = 15).

Similarly to other aspartic proteases (Brik and Wong, 2003). HIV1-protease exhibits a close relationship with water-mediated bridging effects in the crystal structure (Shen et al., 2010). Consequently, the effects of explicit waters were also reflected by the Nwat-MMGBSA workflow (Figure 3A). The high water-density around a wide area at the binding site also confirms the likelihood of the involvement of explicit water during binding process (Figure 3B).

When considering the correlation between the experimental *k*_*i*_ and the predicted binding energies, a significant improvement in *r*^2^ was obtained with the inclusion of a hydration shell of 30–70 water molecules (Figure 3A and Table S41). Although, results with a lower Nwat value showed no significant difference from standard MM-GBSA analyses, suggesting that smaller hydration shells around the ligand might have excluded certain solute-solvent interactions important for binding free energy estimations. However, water-mediated H-bond analyses showed that only one or two stable (occupancy > 20%) water-mediated interactions did involve the ligand, while majority of the bridging water molecules were found between protein residues (Tables S44, S45). Furthermore, crystallographic data provides that only 10–15 water molecules are generally present within 4 Å from the ligand molecules (Olajuyigbe et al., 2011). These imply potential conflicts between the lower numbers of the observed “stable” bridging water molecules to the evidently better binding free energy estimations when higher amount of closest explicit solvent (up to 70) is included. Such conflicts can only be explained when transient water bridges are considered. The averaged free energy contribution of these transient interactions is more likely been captured by Nwat-MMGBSA calculations, whereas not necessarily detectable through population distribution or electron density analyses. Indeed, for example, the inclusion of crystallographic water molecules up to 3.5 Å from the ligand did not provide a clear benefit over standard MM-PB/GBSA approach (Greenidge et al., 2013).

Similar to the penicillopepsin system, the outcomes did not show statistically significant differences between the 1st and 4th ns of MD simulations and were independent from the hardware. Apparently, 4 ns MD simulations performed using CPU averagely provided higher correlation to experiments for Nwat ≤ 20, although the high standard deviations make this result not statistically significant (Figure 3A and Table S41).

#### Test on BCL-X_L_

**Figure 4:**
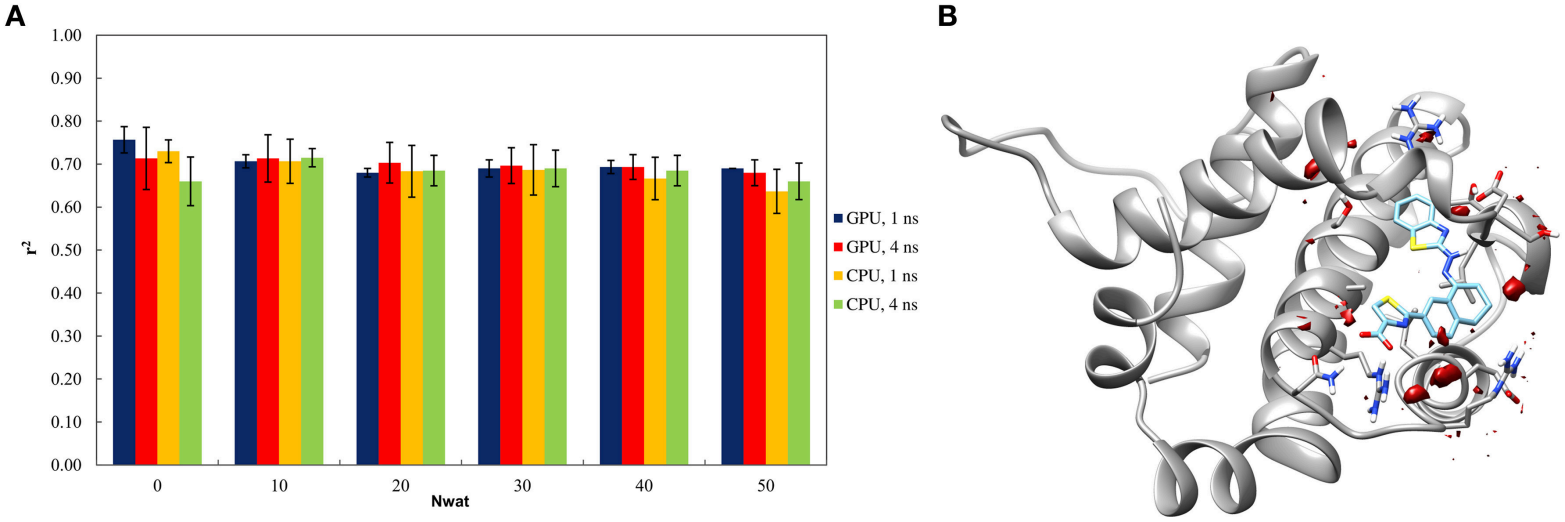
**(A)** Trend of *r*^2^ in dependency of Nwat for BCL-X_L_. **(B)** Water density plot obtained by grid analysis of BCL-X_L_-3ZC4 complex (visualization with Chimera, step = 1, level = 15).

The Nwat-MMGBSA trails up to 50 closest water molecules have no statistical difference from standard MM-GBSA calculations, despite of different hardware environment (Figure 4A). A lower water density was indeed observed around the ligand for BCL-X_L_ system (Figure 4B). This implies that explicit water molecules are playing a less important role in ligand binding, as reflected by the relatively deluding performance of Nwat-MMGBSA compared to MM-GBSA. A relatively high *r*^2^ (~0.7) was indeed consistent throughout the multiple trials for all of the conditions evaluated. These apparently non-effective results, however, positively suggest that the Nwat-MMGBSA method does not impair the statistical outcomes of the estimations for systems where explicit water molecules are deemed less important. Thus, it can be concluded that, even if system-specific tuning is necessary for optimal performance, Nwat-MMGBSA can be safe for binding free energy estimation even without an *a priori* knowledge of the bridging water in the system of interest.

### Retrospective virtual screening test

To assess the performance of the Nwat-MMGBSA method in rescoring virtual screening results, we have chosen two case studies for protein-ligand (PLI) and protein-protein (PPI) interactions, respectively. The first system, AmpC β-lactamase (Usher et al., 1998), was selected from the Dud-E database to provide an example were water plays an active role in the target catalytic cycle. Indeed, the inclusion of an explicit water molecule was found beneficial in previously reported virtual screenings (Powers et al., 2002). Conversely, the second system, the Rac1 protein targeted at the Tiam1 binding site (Worthylake et al., 2000), was chosen because of the availability of reliable in-house activity data, including those of several inactive compounds that were however selected as potential hits by virtual screening studies previously conducted (Ferri et al., 2009, 2013a).

The AUC of the ROC curves was chosen as the main metric of comparison, since enrichment values are not indicated for databases of limited sizes (Enyedy and Egan, 2008). For the AmpC system, the full virtual screening workflow, followed by MD simulation and Nwat-MMGBSA (Nwat = 0, 30, 60 and, for Rac1 only, 100) rescoring, was repeated twice, while for Rac1 a third repetition was added due to a higher variance among the obtained correlations. Docking scores and Nwat-MMGBSA binding energies for AmpC and Rac1 screenings are reported in Tables S46, S47 and Tables S48–S50, respectively.

#### AmpC β-lactamase

The receptor in Dud-E include an explicit water molecule. We did preliminary docking evaluations by including the water, using the “water_molecule” function implemented in PLANTS, but comparable results were obtained (see Figure S12). For this reason, to simplify and standardize the procedure, we decided not to include any explicit water in the docking part of the virtual screening workflow.

**Figure 5:**
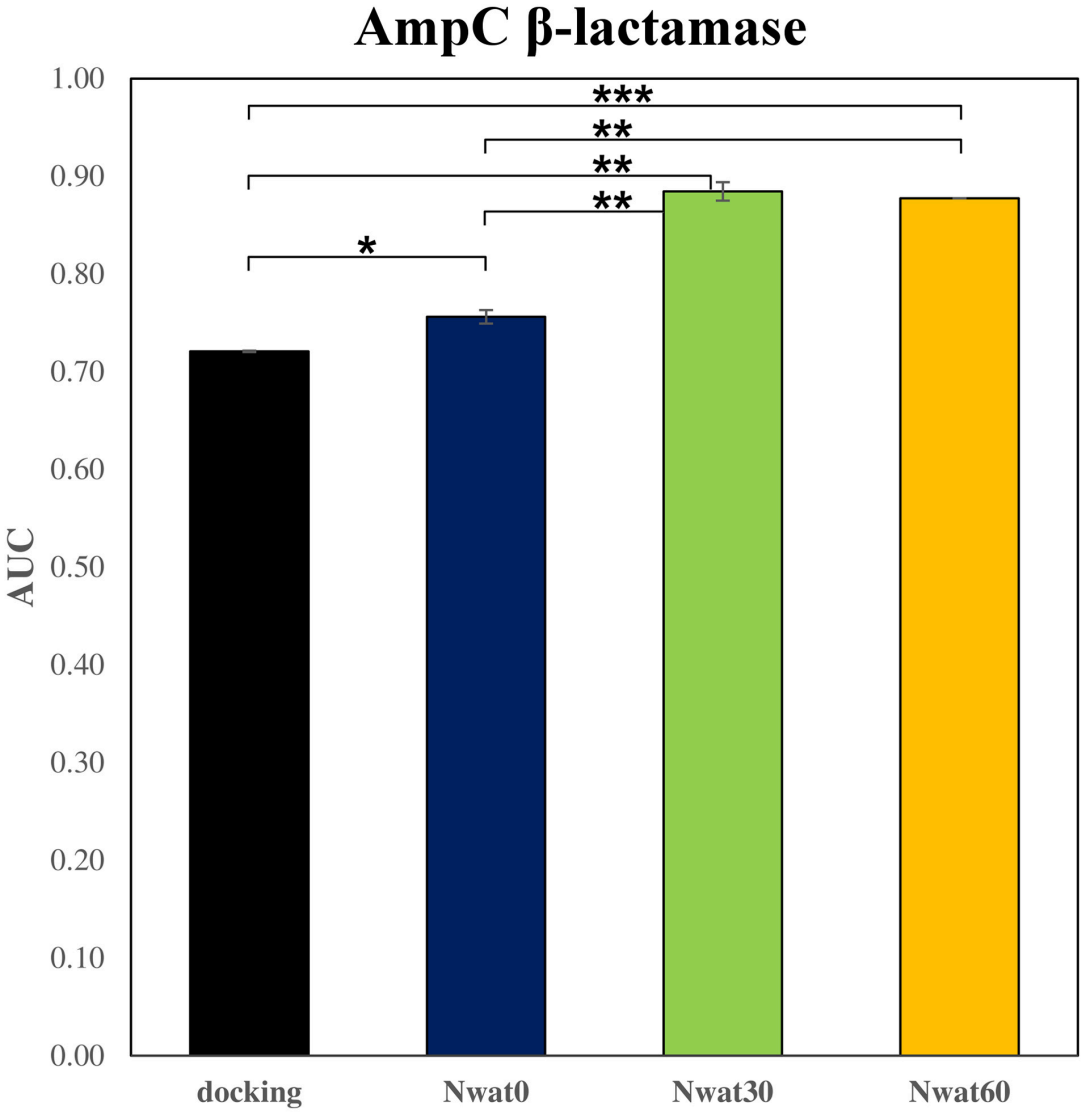
Average ROC AUC values for AmpC β-lactamase. Statistical significance was calculated by *t*-test and is graphically reported only when a significant variation was observed (^*^P < 0.05; ^**^P < 0.01; ^***^P < 0.001).

**Figure 6:**
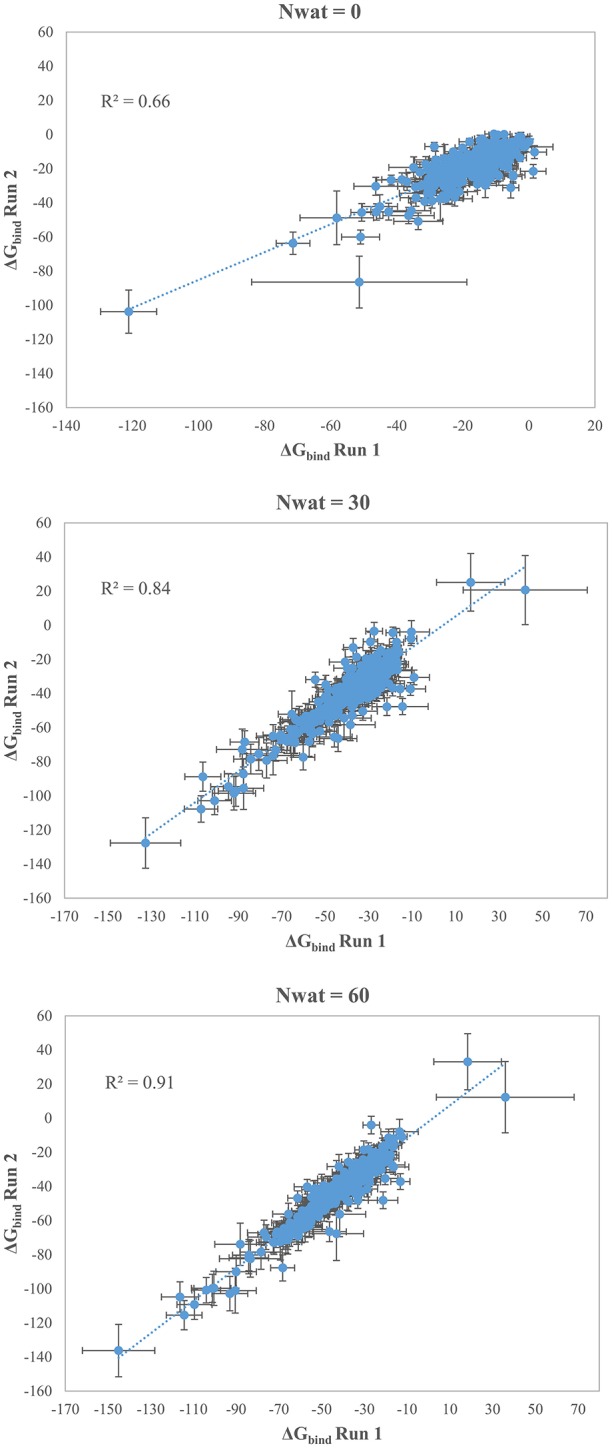
Correlations between the binding energies computed in two independent repetitions (Run 1 and Run 2) for AmpC β-lactamase by standard MM-GBSA (Nwat = 0) or by Nwat-MMGBSA *with* Nwat = 30 or Nwat = 60.

We initially noticed that virtual screening has already provided a decent discrimination of active from decoys with a ROC AUC that averaged at 72.0% (Table 1). The application of the standard MM-GBSA method (Nwat = 0) only provided a barely significant increase of ROC AUC value, respect to docking (Table 1), while improvements appeared once explicit water molecules were included, as shown by the Nwat = 30 and 60 scenarios (Figure 5, Figure S15). Considering that the ROC AUCs for Nwat = 30 and 60 were fully converged, no additional analyses at higher Nwat values were done. Additionally, ligand-to-ligand correlations in calculated free energies were evaluated between the two repeated runs. The standard MM-GBSA run provides a *r*^2^ of 0.66 when correlating the energies obtained by the two repetitions, while Nwat-MMGBSA with Nwat = 30 and 60 resulted in *r*^2^ of 0.84 and 0.91, respectively (Figure 6). This implies that Nwat-MMGBSA rescoring is likely to provide better reproducibility between separate runs. Moreover, the good ligand-to-ligand inter-method correlation between Nwat = 60 and 30 (*r*^2^ = 0.95 and 0.94 for runs 1 and 2, respectively; Figure S13) further confirmed the improvements in reproducibility. Interestingly, a positive binding energy was computed for two ligands by Nwat-MMGBSA calculations, but not by docking or standard MM-GBSA rescoring. The two ligands belong to the decoy set (ligands 088 and 179, Tables S46, S47) and are thus supposed to be poorly ranked. Indeed, by analyzing the binding modes of the decoy 088 (Figure S17), it can be observed that an isopropyl group overlaps with the position occupied by a water molecule present in the crystal structure (Babaoglu and Shoichet, 2006), but not explicitly considered during docking (see Methods). Conversely, decoy 179 does not overlap with the crystallographic water site (Figure S18). However, it can be observed that a solvent-exposed chloropropyl group overlaps to a position occupied by a hydrophilic amino acidic moiety of the crystallographic ligand. In both cases, it appears that Nwat-MMGBSA rescoring can correctly penalize compounds that do not offer an optimal orientation of hydrophobic groups.

**Table 1:** ROC AUC values obtained at different scoring conditions for AmpC β-lactamase.

	**Docking**	**Nwat0^a^**	**Nwat30**	**Nwat60**
*r^2^* ^b^	0.72 ± 0.00	0.76 ± 0.01	0.88 ± 0.01	0.88 ± 0.00
P_docking_		0.019	0.002	<0.001^c^
P_MMGBSA_			0.004	0.002
Δ%_docking_		4.9	22.7	21.7
Δ%_MMGBSA_			17.0	16.0

*Percentage of variation (Δ%_docking_ and Δ%_MMGBSA_) and P values respect to ChemPLP scoring (P_docking_) and to standard MM-GBSA rescoring (P_MMGBSA_) are also reported*.

a *Corresponding to a standard MM-GBSA calculation, with no explicit waters included*.

b *Average of two full repetitions*.

c *0.00001*.

#### Tiam1-Rac1 PPI interface as the PPI test set

Virtual screening targeting PPIs has been suggested as a challenging task, especially when only traditional docking and scoring procedures are used (Bienstock, 2012; Scott et al., 2016). In the past, we have applied standard computational methods to identify and design inhibitors of the Rac1-Tiam1 PPI, thus collecting data on compounds identified as potential hits, but that turned out to be inactive upon experiments (Ferri et al., 2009, 2013a,b; Ruffoni et al., 2014). In addition, we searched the literature to identify compounds that were tested against Rac1 inhibition, but turned out to be inactive (Hernández et al., 2010; Surviladze et al., 2010; Shang et al., 2012; Rahimi et al., 2015; Lu et al., 2017). By this way, the resulting ligand test set shared similar physico-chemical and structural features between the actives and the inactives, thus making this virtual screening a difficult discrimination process to tackle.

The docking protocol was optimized to maximize AUC by evaluating the effect of the different scoring functions available in PLANTS, by variating the binding site radius, the search speed and by using hydrogen bond constraints with residues known to be essential for activity (i.e., Leu70 or Ser71) (Gao et al., 2004). The docking poses were visually inspected to check their consistency with the poses obtained in previous studies (Ferri et al., 2013a). With the optimized protocol and for each library processing condition, all the active compounds showed a similar binding pose, except for ligand109 (Figure S14).

**Figure 7:**
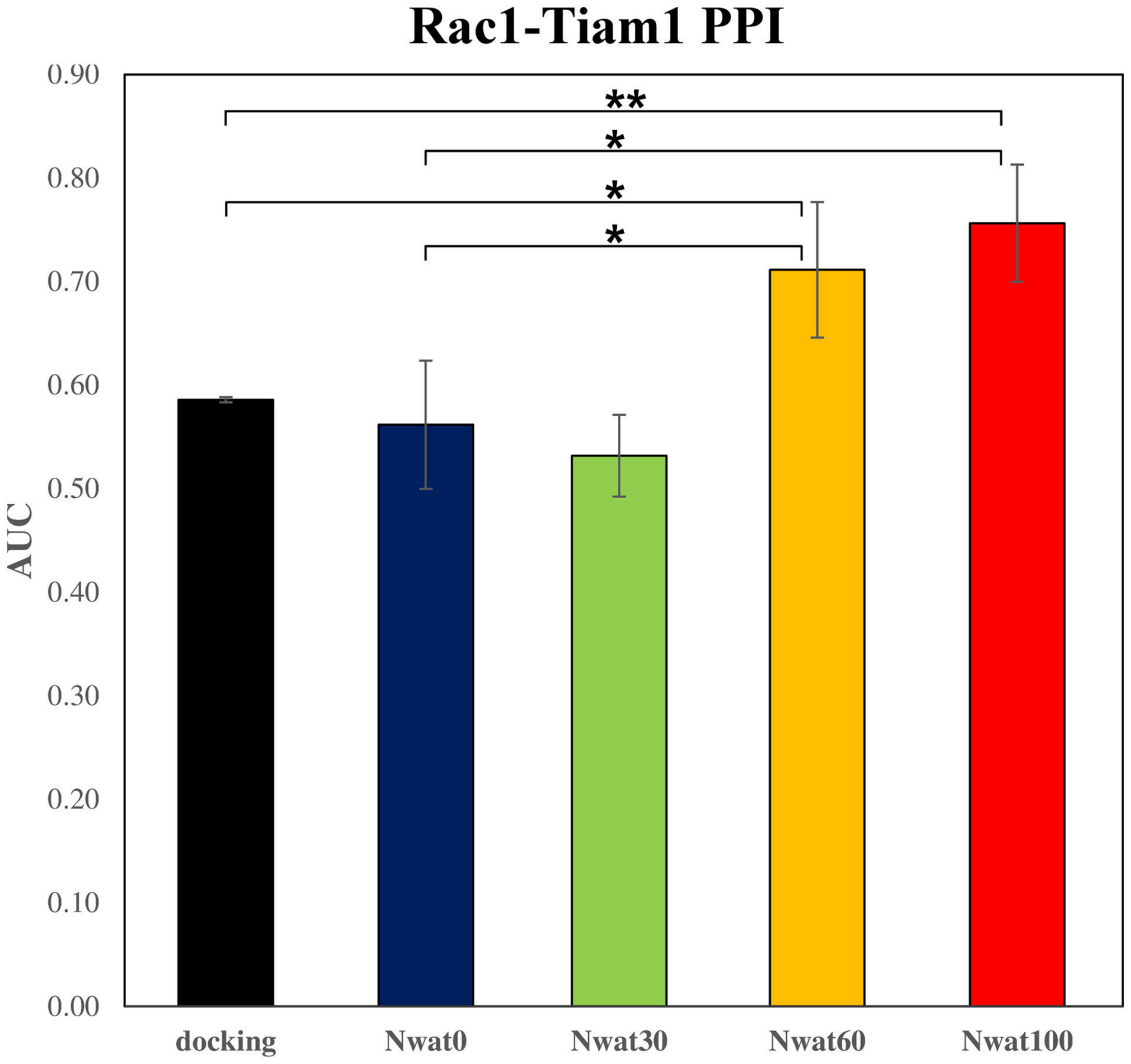
Average ROC AUC values for Rac1-Tiam1 virtual screenings. Statistical significance was calculated by *t*-test and is graphically reported only when a significant variation was observed (^*^P < 0.05; ^**^P < 0.01).

The ROC computed on the scores obtained by docking showed a moderate ability of this procedure in discriminating active from inactive compounds, with AUCs of about 0.6 (Table 2, Figure 7). Considering the strained characteristic of both the target and database, this result is acceptable, if compared to the ROC AUCs obtained in other benchmarks reported by literature (Brozell et al., 2012; Liebeschuetz et al., 2012; McGann, 2012; Neves et al., 2012; Novikov et al., 2012; Repasky et al., 2012; Schneider et al., 2012; Lavecchia and Di Giovanni, 2013; Yuriev et al., 2015).

**Table 2:** ROC AUC values obtained at different scoring conditions for the Rac1-Tiam1 system.

	**Docking**	**Nwat0**	**Nwat30**	**Nwat60**	**Nwat100**
*r^2^* ^a^	0.59 ± 0.00	0.56 ± 0.06	0.53 ± 0.04	0.71 ± 0.07	0.76 ± 0.06
P_docking_		0.536	0.077	0.029	0.007
P_MMGBSA_			0.520	0.045	0.016
Δ%_docking_		−4.1	−9.2	21.4	29.1
Δ%_MMGBSA_			−5.3	26.7	34.7

*Percentage of variation (Δ%_docking_ and Δ%_MMGBSA_) and P-values respect to ChemPLP scoring (P_docking_) and to standard MM-GBSA rescoring (P_MMGBSA_) are also reported*.

a *Average of three full repetitions*.

This time, the application of the standard MM-GBSA (Nwat = 0) rescoring did not provide any significant improvement in the AUC compared to docking (Table 2). Unexpectedly, Nwat-MMGBSA performed with 30 water molecules (Nwat = 30) behaved similarly. Conversely, the ROC AUCs improved of about 20 and 30% after rescoring with Nwat = 60 or 100, respectively (Table 2, Figure 7, and Figure S16). Since the difference in AUC between the two last scenarios was not statistically significant, no additional simulations were conducted at higher Nwat. An improvement in the ROC AUC of about 20–30%, although reproducible and significant (Zhang et al., 2014), might be questionable against the increased computational effort of rescoring with either MM-GBSA or Nwat-MMGBSA. However, in the framework of a lead optimization study, the payback of a simulation that can be easily run on relatively inexpensive hardware can be an increased chance of synthesizing a good molecule. Considering the costs associated with the synthesis of new molecules, having even only a 20% higher probability of preparing an active compound can be considered a rather good result.

## Discussion

When developing new drugs, computational calculation can help in identifying new hits in either the hit-to-lead or lead optimization phases. While the first task is generally performed by using very fast computational methods to screen large databases, the lead optimization phase is generally done by applying more accurate, although more computationally demanding, methods. Indeed, starting from a lead, a virtual library of hundreds-to-thousands congeneric molecules can be generated and evaluated computationally. However, the prioritization of the synthesis of a few derivatives by computational methods might still be quite challenging. In this framework, we optimized a variant on the well-known MM-GBSA method, referred as Nwat-MMGBSA (Maffucci and Contini, 2013, 2016). This approach consists in the inclusion, during the MM-GBSA analysis, of a fixed number of water molecules, which in each frame of the MD simulation are the closest to the ligand, or to a binding interface, and are therefore potentially mediating interactions between the receptor and the ligand. We demonstrated that this approach might improve the correlation between predicted and experimental binding energies up to 50%, compared to the standard MM/GBSA method (corresponding to Nwat = 0), with only a modest increase in computation time (Maffucci and Contini, 2016). Of course, the potential improvement in correlation depends on the role played by water in facilitating the ligand-receptor binding. However, we also found that when water does not play a specific role in mediating this interaction, the application of Nwat-MMGBSA is not detrimental on the quality of correlation, compared to the default approach. In the light of this, we automatized the process and optimized the MD protocol for running simulations on standard workstations equipped with a GPU, on which a full calculation can be completed in about 1–2 h per complex, depending on system size. Indeed, the results obtained by using a single GPU card are comparable, in both quality and duration, with those obtained by running MDs on a relatively large HPC environment (12 nodes with 2 octa core processors per node). Moreover, we also observed that Nwat-MMGBSA analyses provided comparable results when applied on 1 or on 4 ns MD trajectories, thus making this simulation attractive for medium-throughput virtual screenings.

In the second part of this article, we described the integration of Nwat-MMGBSA as a method to rescore docking results in SBVS studies. By applying Nwat-MMGBSA rescoring (Nwat = 60 or 100) we obtained, in both the examples, an increase in the ROC AUCs of between 20 and 30%, compared to the docking scorings or default MM/GBSA (Nwat = 0), depending on the system. In the adopted conditions, we were able to process more than 20 compounds per day using a standard octa core workstation equipped by a single GPU. Although this might appear a quite long time, compared to the thousands of compounds that can be screened per day by docking, the investment becomes reasonable when considering the time and resources required for the synthesis of new molecules. Moreover, we can expect that the fast development of GPU hardware will make MD-based rescoring even faster in short time. Indeed, in 2010 we could run a MD simulation on a Rac1 complex at a speed of 8.7 ns/day on a Tesla C1060 card, while a few years later, the same simulation was run at a speed of 59.3 ns/day on a GeForce GTX TITAN Black card.

Unfortunately, we were not able to find an ideal number of water that need to be included during Nwat-MMGBSA rescoring. Indeed, while Nwat = 30 appeared to be reasonable in most of the examples, including those reported previously (Maffucci and Contini, 2013, 2016), it failed in the Rac1 VS example. Indeed, in this case, at least 60 waters were necessary to observe a significant improvement over docking and standard MM-GBSA, possibly due to the large and solvent-exposed nature of the Rac1 binding site. Conversely, it was recently reported that MM-PBSA calculations on a set of Mnk1 and Mnk2 inhibitors provided improved correlations to experiments only when including up to 10 water molecules (Kannan et al., 2017). This quite low number, compared to other examples, was justified by the rather small interface between Mnk1/Mnk2 kinases and the respective ligands.

## Author contributions

AC coordinated the team, designed the scripts and performed the calculations on Rac1. IM performed the calculations on penicillopepsin, HIV1 and BCL-X_L_. XH provided important updates to the VScreen script. VF performed the calculations on AmpC β-lactamase. IM, XH, and AC wrote the article.

### Conflict of interest statement

The authors declare that the research was conducted in the absence of any commercial or financial relationships that could be construed as a potential conflict of interest.

## Abbreviations

SBVS: structure based virtual screening
VS: virtual screening
ROC: receiving operator characteristic
AUC: area under curve
MD: molecular dynamics
MM-GBSA: molecular mechanics Generalized Born surface area
PPI: protein-protein interaction
SD: steepest descendent
CG: conjugated gradient
NVT: constant number of particles, volume and temperature
NPT: constant number of particles, pressure and temperature.
