# Supplementary material for: An Efficient Implementation of the Nwat-MMGBSA Method to Rescore Docking Results in Medium-Throughput Virtual Screenings
Source: Front Chem. 2018 Mar 5;6:43. doi: 10.3389/fchem.2018.00043 (PMC5844977; doi:10.3389/fchem.2018.00043)
Supplement: Supplementary file 1 [file DataSheet1.pdf]

Supplementary Material

An Efficient Implementation of the Nwat-MMGBSA Method to Rescore Docking Results in Medium-Throughput Virtual Screenings

Irene Maffucci, Xiao Hu, Valentina Fumagalli and Alessandro Contini*

*** Correspondence:** Corresponding Author: alessandro.contini@unimi.it

1 Supplementary Figures and Tables

Figure S1-S3. Penicillopepsin, HIV1-protease and BCL-X_L ligands. S2

Table S1. HIV1-protease complexes with related mutations. S3

Table S2. Database of compounds evaluated in the AmpC VS (SMILES) S4

Table S3. Database of compounds evaluated in the Rac1 VS (SMILES) S13

Tables S4-S15. Predicted binding energies of for penicillopepsin ligands. S16

Tables S16-S27. Predicted binding energies for HIV1-protease ligands. S20

Tables S28-S39. Predicted binding energies for BCL-X_L ligands S26

Tables S40-S42. Correlation values between experimental data and Nwat-MMGBSA predicted binding energies obtained for penicillopepsin, HIV1-protease and BCL- X_L. S30

Table S43. Statistical analyses S32

Tables S44-S45. Water mediated H-bonds for 3NUO and 3NDW with Nwat = 10 and 70. S34

Figure S4-S11. RMSD of the backbone atoms from the starting coordinates for MD simulations of penicillipepsin, HIV1 protease and BCL-X_L S35

Figure S12. Comparison of the ROC AUCs obtained from the AmpC VS with or without including the crystallographic water molecule S42

Figure S13. Correlations between the binding energies computed for AmpC β-lactamase by Nwat-MMGBSA with Nwat = 30 and with Nwat = 60 (results of two independent runs) S43

Figure S14. Rac1 Docked poses of the active compounds S43

Figure S15. ROC AUCs obtained from the two repetitions of the AmpC VS S44

Figure S16. ROC AUCs obtained from the three repetitions of the Rac1 VS S44

Figures S17-S18. Predicted binding poses for AmpC decoys ligand088 and ligand179 S45

Tables S46-S47. Docking scores and Nwat-MMGBSA binding energies for AmpC VS S47

Tables S48-S50. Docking scores and Nwat-MMGBSA binding energies for Rac1 VS S68

2 Supplementary Data

All the scripts, input files and parameters to reproduce the calculations described in this article, together with working tutorials, are provided as a separate zip file. Questions regarding the use of the scripts can be addressed to alessandro.contini@unimi.it

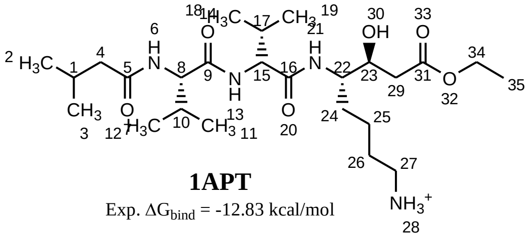

1APT

Exp. ΔG_{bind} = -12.83 kcal/mol

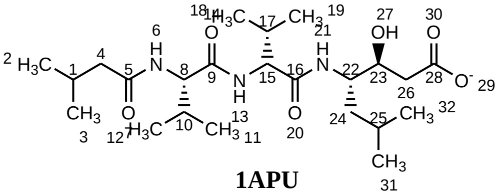

1APU

Exp. ΔG_{bind} = -10.51 kcal/mol

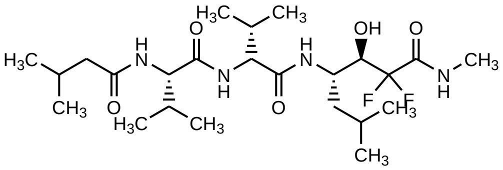

1APW

Exp. ΔG_{bind} = -10.91 kcal/mol

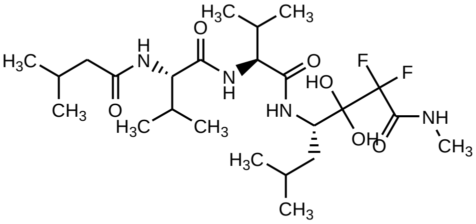

1APV

Exp. ΔG_{bind} = -12.27 kcal/mol

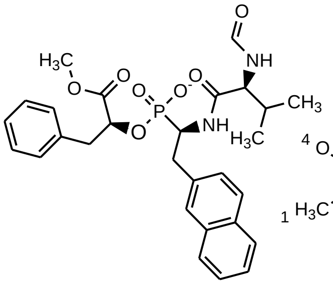

2WEB

Exp. ΔG_{bind} = -7.03 kcal/mol

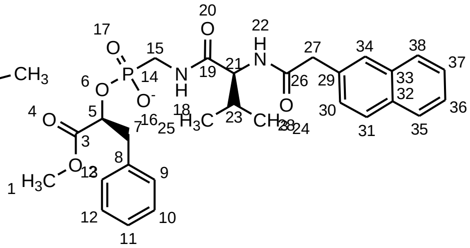

2WEC

Exp. ΔG_{bind} = -6.80 kcal/mol

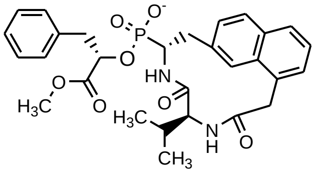

2WEA

Exp. ΔG_{bind} = -8.37 kcal/mol

Figure S1. Penicillopepsin ligands with experimental ΔG_{bind} at the protonation states considered for the analysis.

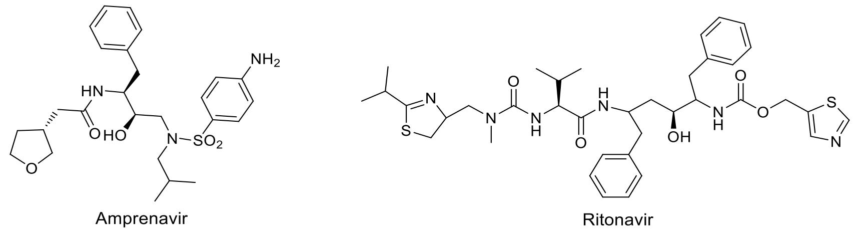

Figure S2. HIV1-protease ligands.

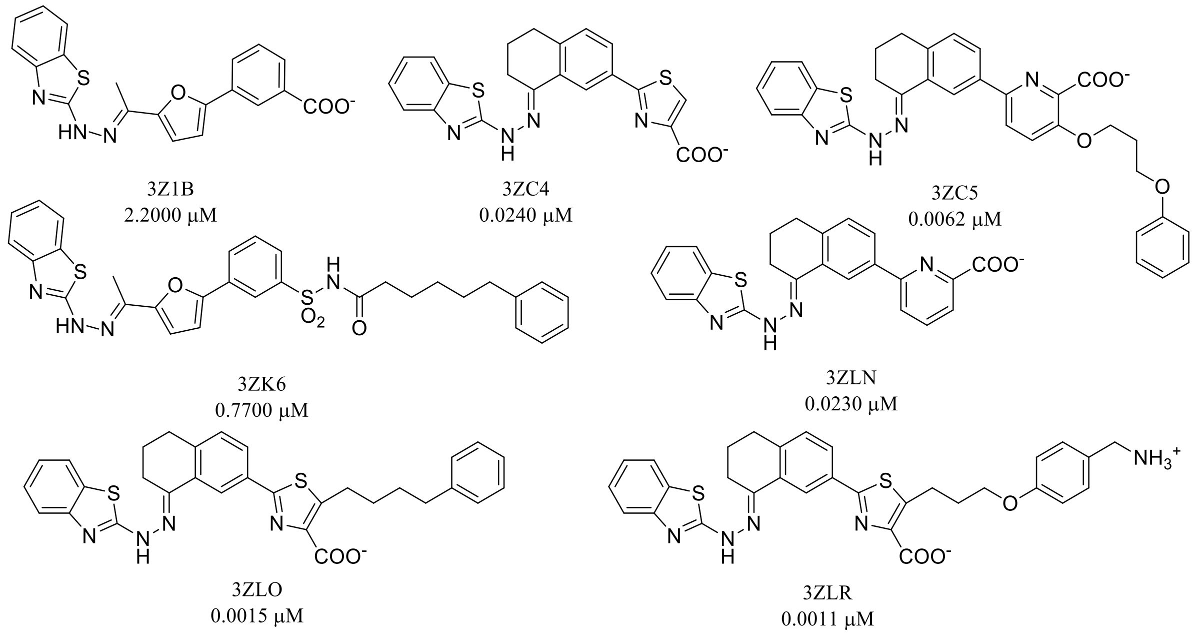

Figure S3. BCL-X_L ligands with experimental IC_{50} at the protonation states considered for the analysis.

Table S1. HIV1-protease complexes with related mutations and k_i values (APV= amprenavir, RTV = ritonavir).

**Complex**	**Mutation**	**Ligand**	**k_i (nM)**
3NU3	Wild type	APV	0.150
3NU4	V32I	APV	1.500
3NU5	I50V	APV	4.500
3NU6	I54M	APV	0.500
3NUJ	I54V	APV	0.410
3NU9	I84V	APV	0.900
3NUO	L90M	APV	0.160
3NDW	Q7K	RTV	0.055
3NDX	Q7K	RTV	0.055

Table S2. Compounds used in the AmpC VS. Inactives are labelled as “0”, actives as “1”.

Lig. #	Name	Mol	activity
1	C05753705	Clc1c([C@H](C(=O)[O-])CC(=O)[O-])ncc(C(F)(F)F)c1	0
2	C52292858	S=C1N([C@H]2[C@@H](C)C2)C(C)=N[N-]1	0
3	C45929952	O=C([O-])c1c(C)c(OC)n(C)n1	0
4	C03871852	O=C([O-])[C@@]1(C)[C@](C(=O)[O-])(C)[C@@H]2O[C@H]1CC2	0
5	C04785749	S=C1NC=Nc2c3c([nH]c12)ccc(F)c3	0
6	C64693634	S(Cc1ccc(C)cc1)C1=NC(=O)CC(N)=N1	0
7	C71481946	O=C1[C@]2(NC(=O)N1)c1c(c(C)cc(C)c1)NCC2	0
8	C17920592	Clc1c(N/C=C/2\C(C(F)(F)F)=NNC\2=O)cccc1	0
9	C49033427	Clc1c(N=NC2C(c3ncccc3)=NNC2=O)cccc1	0
10	C39280606	S=C1N=C(N)C=2C(S[C@H]3C=2CCC3)=N1	0
11	C20581416	S=C1SC(N[C@@H](C(C)C)C)=NN1	0
12	C18140608	O=C1/C(=C\NCc2ccc(C)cc2)/C(C)=NN1	0
13	C53720869	Clc1cc2c(O[C@@H](C(=O)NOC)C2)cc1	0
14	C71606243	S=C1C(F)C(=O)N=C(C)N1	0
15	C65314490	FC(F)(F)C(=O)/N=C/1\N2C(=NN\1)C=CC=C2	0
16	C13597626	O=C1/C(=C\NCc2cnccc2)/C(C)=NN1	0
17	C17950920	S=C1N=C(COC)CC(=O)N1	0
18	C63110904	O=C1NC(=O)c2c(C)nn(C)c2N1	0
19	C16957825	O=C1C=2C(=NN=C(N)NC=2)C[C@@H](C)C1	0
20	C34581412	O=C1[C@@]2(C(=O)Nc3c2cccc3)NC(=O)N1	0
21	C21953365	S(=O)(=O)(/N=C/1\C(=O)CC=CN\1)C	0
22	C19851744	ClC(Cl)(Cl)/C(=N/S(=O)(=O)C)/N	0
23	C69701096	O=C(Nc1[nH]c(C(C)C)nn1)c1cn(-c2c(C)cccc2)nc1	0
24	C41602804	Fc1ccc(CNc2nc(O)c(Cc3occc3)nn2)cc1	0
25	C17878675	O=C(OCCCC)CN1C(=O)[C@](O)([C@@H]2C(=O)N=C(N)S2)c2c1cccc2	0
26	C08726141	Fc1ccc(-c2[nH]c(NC(=O)c3c(OC)cccc3)nn2)cc1	0
27	C65044802	Fc1c(CCC(=O)Nc2[nH]nc(-c3occc3)n2)cc(F)cc1	0
28	C67774090	Fc1ccc(-c2[nH]c(NC(=O)/C(/C#N)=C/c3ccc(C)cc3)nn2)cc1	0
29	C58510858	O=C(Nc1[nH]cnn1)/C(/C#N)=C/c1c(C)n(c(C)c1)-c1ccc(C)cc1	0
30	C66397636	O=C(N[C@@H](C#N)c1ccc(C(C)(C)C)cc1)C=1C(=O)NC(C)=CC=1	0
31	C67660207	FC(F)(F)c1[nH]c(NC(=O)/C=C/c2oc([C@H]3[C@@H](C)C3)cc2)nn1	0
32	C08116584	Fc1ccc(-c2[nH]c(NC(=O)c3ccc(C#N)cc3)nn2)cc1	0
33	C13409505	O=C1N(C)c2c(C(=O)C1C=1/C(=C\c3c(O)cccc3)/C(=O)NN=1)cccc2	0
34	C42605257	Clc1cc(NC(=O)[C@H](Sc2nc(C)[nH]n2)C)c(F)cc1	0
35	C11340818	S(CC(=O)N[C@@H](C)c1ccc(F)cc1)c1n[nH]cn1	0
36	C69774359	Clc1n(C)nc(C)c1CSC1=Nc2[nH]ncc2C(=O)N1	0
37	C00129398	S(CC(=O)Nc1c(F)cc(F)cc1)c1[nH]c2ncccc2n1	0
38	C05465784	O=C1/C(=C(/C)\c2c(O)ccc(C)c2)/C(=O)NC(=O)N1	0
39	C65344544	O=C1C(c2ccc(CO)cc2)=CNC(=O)N1	0
40	C44813136	O=C(Nc1scnn1)[C@@H](C#N)C	0
41	C20064359	S=C1NC(=O)/C(=C\c2oc(-c3cc(C(=O)[O-])c(O)cc3)cc2)/C(=O)N1	0

42	C15012796	Clc1cc(OC)c(O)c(/C=C/2\C(=O)NC(=O)N\2)c1	0
43	C71805141	O=C(Nc1c(O)cccn1)c1ncccc1	0
44	C36882145	O(C)c1c(O)cc(C2=NC(=O)C=CN2)cc1	0
45	C66322335	O(C)c1c(O)cc2N=C(C)NC(=O)c2c1	0
46	C20344738	O=C([O-])c1cc(C(=O)[O-])cc(-n2c(C)c(/C=C\3/C(=O)NC(=O)N/3)cc2C)c1	0
47	C04398438	S(Cc1c(F)cccc1)c1n(Cc2ccccc2)c2C(=O)NN=C(O)c2n1	0
48	C08618899	S(CCC(C)C)c1n(Cc2ccccc2)c2C(=O)NN=C(O)c2n1	0
49	C71621468	O=C(OCC)C1C(=O)N=C(N)S1	0
50	C06045573	O=C/1N(c2ccccc2)N=C(O)\C\1=C\c1n(-c2cc3C(=O)NC(=O)c3cc2)ccc1	0
51	C62716729	C(c1sc2c([n+H]1)CCCCC2)c1nnn[n-]1	0
52	C49591564	O=C([O-])/C=C/c1ccc(CN(C(=O)c2[nH]ccc2)C)cc1	0
53	C37657769	S(=O)(=O)(Nc1cc(C#CCO)ccc1)C(C)C	0
54	C57534217	S(=O)(=O)(Nc1cc(C)cc(C)c1)CCCO	0
55	C71519308	S(=O)(=O)(NO)c1c(C)cc(C)c(C)c1	0
56	C00235891	O=C1C2=N/C(=C\Nc3c(O)cccc3)/C=CC2=CC=C1	0
57	C37552662	S(=O)(=O)(Nc1c(F)cc(C#CCCO)cc1)N1CCCC1	0
58	C41721189	O=C(/N=C\1/SC=C(C(C)C)N/1)/C(/C#N)=C\c1ccc(O)cc1	0
59	C37577510	S(=O)(=O)(/N=C\1/NOC(C)=C/1)c1c(C#CCO)cc(C)cc1	0
60	C19837149	Clc1c(O)ccc(C(=O)NCCOC)c1	0
61	C00249507	O(C)c1ccc([C@@H]2NN/C(=C\3/C(=O)c4c(C/3=O)cccc4)/C2)cc1	0
62	C37549660	O=C(Nc1sc(C#CCCO)cn1)c1n(CC)ccc1	0
63	C37552353	S(=O)(=O)(/N=C/1\SC(C#CCO)=CN\1)N1[C@H](C)CCCC1	0
64	C20709644	O=[N+]([O-])c1cc([C@H]2Nc3c(C(=O)N(C)C4CCCCC4)cccc3[C@H]3[C@@H]2CC=C3)c(O)cc1	0
65	C36927218	Clc1c(C)c(N2C(=O)C([O-])=NC2=O)ccc1	0
66	C55303393	O=C1Oc2c(N1)ccc([C@H](O)[C@H]1OCCOC1)c2	0
67	C49575329	S=C1SC(NCC[N+H]2CCCCC2)=NN1	0
68	C54128923	S=C1SC(NCC[N+H](C(C)C)C)=NN1	0
69	C65341679	S(=O)(=O)(Nc1cc(-c2cc(O)c(C#N)cc2)ccc1)C	0
70	C37395041	O=C(Nc1scnn1)C[N+H2]C1CC1	0
71	C49660525	O=C1N(CC)c2nc(N)sc2C(=O)N1	0
72	C11919853	O=C1NN=C(c2sccc2)c2sc(N)nc12	0
73	C05211034	S([C@@H](C(=O)NC1CC1)C)c1nc(-c2ccc(C)cc2)[nH]n1	0
74	C67284644	Clc1cc(Nc2c(C(=O)N3CCSCC3)[nH]nn2)ccc1	0
75	C12506010	FC(F)(F)[C@]1(O)C(=O)N/C(=C/C(=O)OCC)/C1	0
76	C48505853	Brc1cc(/C(/[O-])=N\S(=O)(=O)CCCC)[nH]c1	0
77	C69675862	S(=O)(=O)(/N=C\1/NC=C(F)C=C/1)c1c2c([nH]c1)nccc2	0
78	C70131456	S(=O)(=O)(C([C@H](O)c1cc2OC(=O)Nc2cc1)(C)C)C	0
79	C05999572	S([C@@H](C(=O)Nc1[nH]cnn1)c1ccccc1)c1ccccc1	0
80	C63473443	O(C)c1ccc([C@@H]2NC(=O)c3n[nH]c(-c4sccc4)c23)cc1	0
81	C44926420	O(C)[C@@H]1[C@H]([N+H2][C@H](C)c2nnn[n-]2)CCCC1	0
82	C69582423	FC(F)(F)c1[nH]c(NC(=O)c2n(C)nc(C(C)(C)C)c2)nn1	0
83	C35739677	S=C1N([C@H](C)c2occc2)N=N[N-]1	0
84	C39113249	O=C1[N-]c2onc(C)c2C(=O)C1	0

85	C21079621	ClC=1C(=O)/C(=C\NNc2[nH]c(SCCOC)nn2)/C=C(Cl)C=1	0
86	C00124470	S(C)C1=C(C#N)[C@@](O)(c2sccc2)N=C(C(F)(F)F)N1	0
87	C36748156	S(=O)([O-])(=NNC(=O)CC(C)C)c1cc([N+](=O)[O-])c(O)cc1	0
88	C22591123	P(=O)([O-])([O-])[C@@]1(C)P(=O)([O-])Oc2c1cccc2	0
89	C40176456	O=C1NC(=O)c2n3C(=O)C=Cc3nc2N1	0
90	C61591519	S=C1N(c2c(C)cccc2)C(=O)[N-][N-]1	0
91	C12176818	S=C1S/C(=C/Nc2sccn2)/C(=O)[N-]1	0
92	C17992321	S=C1NC(=O)/C(=C\c2oc(-c3c(O)ccc([N+](=O)[O-])c3)cc2)/C(=O)N1	0
93	C43479560	Clc1c(O)ccc(CNc2c(-n3nccc3)nccc2)c1	0
94	C48416362	O=C(N[C@@H](C)c1cnccc1)c1nnc(O)cc1	0
95	C70419275	Clc1c(NS(=O)(=O)c2nc(CC)[nH]c2)ccc(F)c1	0
96	C48733940	Fc1ccc([C@H](CC(=O)Nc2[nH]cnn2)C)cc1	0
97	C68949934	O=C(Nc1[nH]cnn1)CC[C@@H]1OCCCC1	0
98	C03457151	Clc1cc(C)c(OCC2=N[N-]C(=S)N2)cc1	0
99	C35409733	Fc1c(O)c(F)c(C(=O)N[C@H](C(=O)[O-])CC)cc1F	0
100	C52005052	O=C([O-])/C=C/C(=O)N[C@@H]1[C@@H](O)CCCC1	0
101	C71165866	O=C([O-])/C(=C(\C(=O)N[C@@]1(CO)C[C@@H](C)CCC1)/C)/C	0
102	C04784407	S=C1n2nc(O)cc2N=C(c2c(F)cccc2)N1	0
103	C42514979	O=C(N(CCO)C1CCCC1)C=1N=CC(=O)NC=1	0
104	C70886803	O=C(N[C@@](CO)(CC)C)C=1N=CC(=O)NC=1	0
105	C43751384	Clc1nccc(C(=O)Nc2[nH]c(C(F)(F)F)nn2)c1	0
106	C08619782	S=C1N(c2c(F)cccc2)C(=O)c2c([nH]nc2)N1	0
107	C48794375	Brc1cc(NC(=O)CSc2n[nH]cn2)c(C)cc1	0
108	C23141138	O=C(Nc1n[nH]cn1)[C@@H]1Sc2c(cccc2)C1	0
109	C71766630	Clc1c(O/C=C/2\OC([O-])=NN\2)ccc(Cl)c1	0
110	C45136139	S=C1N(C[C@H]2C[N+H](C(C)C)CC2)N=N[N-]1	0
111	C58332804	Clc1c(C(=O)Nc2[nH]c(C(C)C)nn2)cc(Cl)s1	0
112	C49474190	S=C1[N-][N+H]=C(c2ccc(C)cc2)N[N-]1	0
113	C05945177	S(SC1=NNC(=S)C=C1)C1=NNC(=S)C=C1	0
114	C69055083	Fc1c(O)c(F)c(C(=O)NOCC(=O)[O-])cc1F	0
115	C37405181	O=C1c2c(NN=C1)cc1OCOc1c2	0
116	C04878981	O=C([O-])c1c(C(=O)C)c(C)c(C(=O)[O-])[nH]1	0
117	C20583359	S=C1N([C@H]2CS(=O)(=O)CC2)C=N[N-]1	0
118	C49450205	Fc1c(C(=O)Nc2n[nH]c(CC)c2)c(O)cc(F)c1	0
119	C39334766	[N+H3][C@H]1[C@@H](c2nnn[n-]2)C2=C(S1)CCCC2	0
120	C62716726	C(c1sc2c([n+H]1)CC[N+H2]C2)c1nnn[n-]1	0
121	C71467882	O=C1n2nc(-c3ccc(C)cc3)cc2N=C(C[N+H3])C1	0
122	C51989901	Clc1c(O)ccc(C(=O)N[C@@H]2[C@@H](O)CCCC2)c1	0
123	C66584053	S(=O)(=O)(Nc1c2n[nH]cc2c(C)cc1)C1CCCCC1	0
124	C26465236	O=C(NCCCC)/C(=N\[O-])/C#N	0
125	C49913910	S(=O)(=O)(N[C@H](C#N)c1c(C)cccc1)c1c(C)[nH]nc1C	0
126	C03106116	O=C([O-])/C(=C/C(=O)NN(C)C)/C	0
127	C48996328	O=C(N1[C@@H](C(=O)[O-])C[C@@H](O)C1)N1CC[N+H](C2CC2)CC1	0

128	C46091078	O=C(N[C@@](C#N)(C(C)C)C)[C@H](C)[N+H]1CCC(CO)CC1	0
129	C70416995	S(=O)(=O)(N1[C@@H](c2[nH]ccc2)CCC1)c1nc[nH]c1	0
130	C35015891	O=C1C2([N+H2][C@H](c3occc3)N1)CCCC2	0
131	C70681329	S=C1N(C[C@H]2NC(=O)CC2)C=N[N-]1	0
132	C71467902	O=C1n2nc(-c3occc3)cc2N=C(C[N+H3])C1	0
133	C06073484	O=C([O-])C1=NC=2N(C(=O)C3=C(N=2)CCC3)N1	0
134	C01728517	S=C(N=N/C=C\1/NC=CC=C/1)N=NCc1ncccc1	0
135	C69971460	O=C(N1C[C@H](C[N+H]2CCOCC2)CCC1)c1c(O)ccc(C(C)C)c1	0
136	C17326685	S=C1NC(=O)C2NC(=O)C(=O)N=C2N1	0
137	C44931991	Nc1c2c(ccc1)C[N+H]([C@H](C)c1nnn[n-]1)C2	0
138	C18189761	N#C/N=C(/N[C@@H](C(C)(C)C)C)\Nc1cc[n+H]cc1	0
139	C19359254	O=C([O-])c1oc(C[N+H]2CC[N+H](Cc3oc(C(=O)[O-])cc3)CC2)cc1	0
140	C17078663	S(=O)([O-])(=N/[N+H]=C(/C)\c1c(O)c2c(cc1)cccc2)c1cc([N+](=O)[O-])ccc1	0
141	C18124909	FC(F)(F)/C(/[O-])=N\c1[nH]c(-c2[nH]nc(NC(=O)C(F)(F)F)n2)nn1	0
142	C39302923	S(=O)(=O)([O-])[C@@]12C(C)(C)[C@@H](CC1=O)CC2	0
143	C21993392	[PH](=O)([O-])CC	0
144	C04878318	BrC=1C(=O)NNC=1C	0
145	C44496437	S[C@H](C)c1cn(C)nc1	0
146	C08116500	Fc1ccc(-c2[nH]c(NC(=O)/C=C/c3occc3)nn2)cc1	0
147	C69742907	Fc1c(C(=O)Nc2[nH]cnn2)ccc(-c2ccc(F)cc2)c1	0
148	C62869418	Brc1cc(O)c(C(=O)N2C[C@](C(=O)[O-])(C)CCC2)cc1	0
149	C52266754	O=C([O-])C(C)(C)[C@@]1(O)[C@H](C)CCCC1	0
150	C41291510	Clc1cc(C)c(OC[C@@H](O)CC(=O)[O-])cc1	0
151	C04362873	SCC(O)CS	0
152	C26440976	ClCCCc1c(O)nc(C(F)(F)F)nc1O	0
153	C54930868	Oc1nnc2c(c1)CSCC2	0
154	C36533270	Oc1c2c(n(C)nc2)ccc1	0
155	C51002199	Brc1c(O)c(Br)cc(/C=C(\C#N)/c2[nH]c3c(n2)ccc(C)c3)c1	0
156	C29546673	Clc1c(C2=C(C#N)C(=O)NC(c3c(O)cccc3)=C2)cccc1	0
157	C38864543	FC(F)C=1NC(=O)C=CC=1	0
158	C37154404	S([C@H]1[C@@H](C#N)CC[C@@H](C)C1)c1n[nH]cn1	0
159	C04149778	ClCCCSc1ncnc2nc[nH]c12	0
160	C69947966	S(=O)(=O)(N1CCSCC1)c1nc[nH]c1	0
161	C41669608	OCCN(CC)c1nc2c(O)cccc2cc1	0
162	C20233592	Oc1cnc(-c2ccc(-c3[nH]ncc3)cc2)nc1	0
163	C28164987	S(Cc1[nH]nc(O)c1)c1nc(C)nc2sc(CC)cc12	0
164	C06094144	O=C1c2c(O)cc(O)cc2Oc2c1cc(O)cc2	0
165	C00091681	S(Cc1[nH]nc(O)c1)c1ncccc1	0
166	C64218986	O=C1[C@H](c2cc(O)c(O)cc2)Oc2c(O)c(C)ccc12	0
167	C44496871	Oc1nnc(-c2cn(C)nc2)cc1	0
168	C20158895	S=C1N(N)C(=O)C(c2ccc(C)cc2)N=N1	0
169	C41600921	Oc1nc(N2C[C@H](C)C[C@H](C)C2)nnc1	0
170	C51036757	S(CC(=O)c1c(O)ccc(F)c1)c1ncnc2[nH]cnc12	0

171	C18164784	c1(-c2cnccc2)[nH]c(-c2ccncc2)nn1	0
172	C59457502	S(C(F)(F)C(=O)[O-])c1[nH]c2c(n1)ccc([N+](=O)[O-])c2	0
173	C06493998	O=C1C(c2ccccc2)=CC(C#N)=C([O-])N1	0
174	C06413535	O=C([O-])[C@@]1(C)C(C)(C)[C@@H](c2[nH]c3c(n2)cc(C)cc3)CC1	0
175	C50213781	O=C([O-])c1c(C(C)(C)C)n(CCO)nn1	0
176	C00120763	S=C1N(c2cc(F)ccc2)C(O)=N[N-]1	0
177	C13126405	O(C)c1cc(O)c(-c2n[nH]c(-c3ccccc3)c2)cc1	0
178	C03160614	O=C1/C(=C/c2c(O)cc(O)cc2)/SC(=O)[N-]1	0
179	C13678569	P(=O)([O-])([O-])C(P(=O)([O-])[O-])(O)Cc1cc(C2=CNC(=O)C=C2)ccc1	0
180	C51394017	S(C[C@H]1O[C@H](C(=O)[O-])CC1)c1n[nH]cn1	0
181	C69899369	O=C([O-])[C@H](C(C)C)[C@@]1(O)C[C@@H](C)OCC1	0
182	C17878987	O=C1N(Cc2sccc2)c2c([C@@]1(O)[C@@H]1C(=O)N=C(N)S1)cccc2	0
183	C06021875	S=C1SC(NC(=O)C2CC2)=NN1	0
184	C40258948	Clc1ccc([C@@H](O)c2cc3OC(=O)Nc3cc2)cc1	0
185	C08927444	Clc1c(Nc2nc(O)c(C)nn2)cccc1	0
186	C41601460	Oc1c(Cc2sccc2)nnc(NCC)n1	0
187	C37190308	S(CC=1OC=C(O)C(=O)C=1)c1c(N)cccn1	0
188	C08830348	O=C1S/C(=C/c2ccc(O)cc2)/C(N)=N1	0
189	C50007182	O=C(Nc1nc(-c2sc(C)cc2)[nH]n1)Cc1c(C)cccc1	0
190	C71289372	Clc1cc2NC(=S)N(N)C(=O)c2cc1	0
191	C50290339	O=C([O-])C1(C(=O)NC2CCC(O)CC2)CCC1	0
192	C50290341	O=C([O-])C1(C(=O)NC2CCC(O)CC2)CC1	0
193	C19874316	Oc1c(-c2sc(N)nn2)cc(C)cc1	0
194	C19874341	Clc1c(O)ccc(-c2sc(N)nn2)c1	0
195	C13647098	Brc1c(O)c(/C=N\NC(=O)C23C[C@@H]4CC(C2)C[C@H](C3)CC4)cc([N+](=O)[O-])c1	0
196	C65355706	Fc1cc(O)cc(C=2NC=CC(=O)C=2)c1	0
197	C65589724	Clc1c(O)ccc(/C=C/c2sc(C(=O)NC)cn2)c1	0
198	C37070726	Clc1cc(CNc2c(OC)nccc2)c(O)cc1	0
199	C19836520	Clc1c(O)ccc(C(=O)NCC#C)c1	0
200	C19838389	O=C(NCCCC)c1ccc(O)cc1	0
201	C11565189	O=C1C=C(N[C@@H](CO)CC)CCC1	0
202	C19885521	Clc1cc(C[N+H2]CC(=O)OC)c(O)cc1	0
203	C49512110	Clc1c([C@H](NC(=O)c2c(O)cccn2)C)cccc1	0
204	C32575643	S(=O)(=O)(N=NCc1[nH]ccc1)c1cc2c(cc1)cccc2	0
205	C44490973	Brc1cc(O)c(C(=O)N(CC(=O)NC)CC)cc1	0
206	C54370013	O=C(N1[C@H](C)C[N+H2]CC1)c1c(O)cc(OC)cc1	0
207	C63749024	O=C(N)CN(C(=O)c1c(O)cc(C(C)C)cc1)C	0
208	C61684500	S(=O)(=O)(/N=C/1\NOC=C\1)c1ccc(C#CCCO)cc1	0
209	C41652487	O=C(C)c1cc(OCC)c(O)c(N)c1	0
210	C56701362	S=C(N=NCc1c(O)cccc1)NC	0
211	C19205478	Oc1c(-c2[n+]([O-])c(CCCC)c(-c3ccccc3)[nH]2)cccc1	0
212	C35522837	S(C(C)C)c1nc(-c2sccc2)[nH]n1	0
213	C12921808	O=C(Nc1[nH]c(-c2ccc(C)cc2)nn1)C1CC1	0

214	C28681289	Fc1ccc(-c2[nH]c(NC(=O)CCC)nn2)cc1	0
215	C20390511	Oc1c([C@H]2NN=C(c3oc(C)cc3)C2)ccc(C)c1	0
216	C38007139	S(=O)(=O)(NCc1c[nH]nc1)N1CCC(C)CC1	0
217	C65056746	O=[N+]([O-])c1c2[nH]c(C(=O)Nc3[nH]c(-c4sccc4)nn3)cc2ccc1	0
218	C70713045	O=C([O-])[C@@](O)(COC1CCCCC1)C	0
219	C70713125	Clc1c(C)cc(OC[C@@](O)(C(=O)[O-])C)cc1	0
220	C15015423	S(C)c1nc(O)c(-c2c(N)cccc2)nn1	0
221	C66323443	Brc1[nH]c2c(Cl)nc(N)nc2c1	0
222	C35686179	Brc1c(Br)c(NS(=O)(=O)CC)cc(Br)c1O	0
223	C05444066	Brc1ccc(CSc2[nH]c(NC(=O)C)nn2)cc1	0
224	C39189859	O=NC1=C([O-])CCCC1	0
225	C21596355	S=C(NC(=O)C(C)C)Nc1c(O)cccn1	0
226	C70713271	O=C([O-])[C@@](O)(COc1cnccc1)C	0
227	C71121726	FC(F)(F)c1[nH]c(NC(=O)[C@@H](OC2CCCC2)C)nn1	0
228	C69559766	Clc1cc(Cl)cc(O[C@@H](C(=O)Nc2[nH]cnn2)C)c1	0
229	C65336480	SC=1N=N[C@H](C)C=1	0
230	C08251247	O=C1SCC(=O)C1	0
231	C32627807	[O-]/[N+H]=C/1\[C@H]2[N+H](CC\1)CCC2	0
232	C49398945	FC(F)(F)c1nc(NC(=O)[C@H]2[C@@H](c3occc3)C2)[nH]n1	0
233	C05226028	S=C1SC=NN1	0
234	C71166685	Fc1c(F)cc(-c2[nH]c3c(n2)ccnc3)c(N)c1	0
235	C42013436	FC(F)(F)Cc1[nH]c(-c2ccccc2)nn1	0
236	C04311295	Brc1c([N+](=O)[O-])nc(C)[nH]1	0
237	C26423248	S=C1NC=Nc2n(C)nnc12	0
238	C04730747	S=C1NC=2C(=O)N(C)C(=O)NC=2N1	0
239	C57307439	FC(F)(F)c1nc(NC(=O)[C@H](OC)C)[nH]n1	0
240	C15924556	O=C(C)c1c(O)c([C@@H](CC)C)[nH]c1O	0
241	C06796039	S=C(N)N1C(=O)C(N=O)C(C)=N1	0
242	C01530958	ClC(Cl)(Cl)[C@H](P(=O)(OC)OC)O	0
243	C37128358	Clc1cc(CNc2ccc(-n3nccn3)cc2)c(O)cc1	0
244	C66054661	Brc1ccc(-c2c(O)c(C(=O)OCC)n[nH]2)cc1	0
245	C20089055	O=C1NN=C(O)c2c1c(NC[C@@H](CC)C)ccc2	0
246	C68880138	FC(F)(F)[C@H](O)COc1ccc(CC#N)cc1	0
247	C35201112	Clc1c(Cl)cc(C(=O)NCCCO)[nH]1	0
248	C04244998	N#C/N=C/Nc1ccc(-c2ccc(N/C=N/C#N)cc2)cc1	0
249	C35279647	O=C([O-])[C@]12C(C)(C)[C@](C)(/C(=N\N)/C1)CC2	0
250	C16857803	S=C1NC(=O)C(N=O)C(=O)N1	0
251	C05046420	ClC[C@H](O)COP(=O)([O-])[O-]	0
252	C55161009	O=C1C(O)=CCS1	0
253	C19690527	O=[N+]([O-])c1c(O)cccc1O	0
254	C43683180	S(Cc1sc(C#CCO)cc1)c1n[nH]cn1	0
255	C59649067	Clc1c(C=O)cn[nH]1	0
256	C49181670	O=C1O[C@@](O)(C)C=2O[C@H](/C=C/C)[C@@H](O)[C@H](O)C1=2	0

257	C14827650	O=C1N2C([C@@H](O)CC2)=Nc2c1cc(O)cc2	0
258	C58007344	OCc1oc2c(n1)cc(O)cc2	0
259	C05117309	Oc1c(-c2[nH]c3c(n2)cncc3)cccc1	0
260	C40488325	Oc1c(-c2[nH]c(CC)nn2)cccc1	0
261	C37491034	S([C@@H]1[C@H](O)CCCC1)c1n[nH]cn1	0
262	C50330892	O[C@H]1c2sc(-c3n[nH]cn3)nc2CCC1	0
263	C19886021	Brc1c(O)ccc([C@@H](c2c(C)[nH]nc2O)c2c(O)[nH]nc2C)c1	0
264	C44713547	O=C1NC(C#N)=CC=C1	0
265	C37191919	S(CC=1OC=C(O)C(=O)C=1)c1cnc(N)cc1	0
266	C49175419	O=C(Nc1[nH]cnn1)c1sc(CC)nc1	0
267	C58386988	O=C(Nc1[nH]cnn1)C(C)n1nccc1	0
268	C54931059	Oc1cc2oc3c(nnc(NC)c3)c2cc1	0
269	C54930973	Oc1cc2oc3c(nnc(N)c3)c2cc1	0
270	C26252164	S(C(C)(C)C)CC(=O)Nc1[nH]c(C(F)(F)F)nn1	0
271	C37336027	Fc1cc(C(=O)C[N+H]([C@H](CC)C)C)c(O)cc1	0
272	C04641258	O=[N+]([O-])C[C@@H](c1c(-c2c(O)cccc2)[nH]nc1O)c1ccc(OC)cc1	0
273	C35539829	S(CCC)c1[nH]c(N=NCc2c(O)ccc([N+](=O)[O-])c2)nn1	0
274	C71023433	S=C1N([C@@H]2COCCC2)N=N[N-]1	0
275	C36047656	Clc1nc(C(C)C)[nH]n1	0
276	C40166094	ClC/C(/O)=N\N1C(=O)C2(NC1=O)CCCC2	0
277	C21985804	BrCC=1NC(=O)C([N+](=O)[O-])=CC=1	0
278	C41894378	O=C(NCC#C)c1cc2[nH]nnc2cc1	0
279	C67348972	Brc1cnc(Sc2nc(Cc3sccc3)[nH]n2)nc1	0
280	C22016501	O=[N+]([O-])c1c(NC)ccc(/C=C/2\C(=O)c3c(O\2)cc(O)cc3)c1	0
281	C20566198	S(=O)(=O)(N1CCCCCC1)c1cc(N)c(O)cc1	0
282	C26507275	O=C1N/C(=N\N=C/c2cc(O)c(O)cc2)/SC1	0
283	C49277952	O=C(NC[C@@H]1CC=CCC1)c1nn[nH]c1	0
284	C48941524	O=C(N[C@H](C(C)(C)C)c1sccc1)c1nn[nH]c1	0
285	C70227460	O=C(N[C@H](CC)C)c1nn[nH]c1	0
286	C59382120	ClC=1C(=O)/C(=C\NN(CC)c2n[nH]cn2)/C=C(Cl)C=1	0
287	C59391068	FC(F)(F)c1nn(-c2nc(C)[nH]n2)c(C)c1	0
288	C66339779	O=C1NC=Nc2sc(O)cc12	0
289	C13523790	O=C1C(O)=CCC(=O)N1	0
290	C19921476	Oc1c(CNc2c(C#N)cccc2)ccc(O)c1	0
291	C05925899	Oc1n(-c2ccc(N)cc2)nc(C)c1	0
292	C70819542	Cc1noc(-c2[nH]c3c(n2)cncc3)c1	0
293	C19265400	O=C1N(Cc2c(N)cccc2)N=C(O)C=C1	0
294	C13597953	S=C1N2c3c(NC(=O)C2=N[N-]1)cccc3	0
295	C49569665	S=C1N([C@@H]2[C@@H]3[N+H](CC2)CCC3)N=N[N-]1	0
296	C57544366	S(=O)(=O)(N[C@@H](C#N)C(C)C)c1c(C)[nH]nc1C	0
297	C06863423	O=C(Nc1[nH]cnn1)c1cnccc1	0
298	C36164606	O=C(Nc1scnn1)c1c[nH]nc1	0
299	C70801477	Nc1cc(-c2[nH]c3c(n2)ccnc3)cnc1	0

300	C04723991	Brc1cc(-c2[nH]c(SCC(=O)N3CCCC3)nn2)c(O)cc1	0
301	C68574043	BrC(C(F)(F)F)(C(F)(F)F)C(=O)Nc1[nH]cnn1	0
302	C26509211	S=C1N(N)C(COc2c(C)cccc2)=N[N-]1	0
303	C53993787	FC(F)(F)[C@@H]1NC(=O)/C(=C(/O)\[O-])/CC1	0
304	C50290939	O=C([O-])C1(C(=O)Nc2c[nH]nc2)CCC1	0
305	C19702731	Oc1c(C=2Sc3n(c(C)nn3)N=2)ccc(N)c1	0
306	C04831683	BrC=1[C@H](C)Nc2n(C=1O)nc(SC)n2	0
307	C32500915	S(=O)(=O)(N)c1c(C)oc(CO)c1	0
308	C64354793	O=[N+]([O-])c1c(C)[nH]nc1C(=O)NOC	0
309	C43705044	O=C([O-])[C@H]1OCC[N+H]([C@H]2[C@H](O)CCCC2)C1	0
310	C62748414	Fc1cc(O)c([C@H]([N+H2][C@@H]2C(=O)NCCC2)C)cc1	0
311	C06444995	O=[N+]([O-])c1c(-c2ccc(N)cc2)[nH]cn1	0
312	C04552279	S(C)c1[nH]c2C(=O)NC(=O)Nc2n1	0
313	C49474272	S(C)c1nn2C(=N)CC(=O)Nc2n1	0
314	C49474284	BrC1C(=N)n2nc(C)nc2NC1=O	0
315	C40166096	S=C1N(C)C(=O)c2c(n[nH]c2)N1	0
316	C14983852	Fc1cc(C(=O)[O-])c2c(C=O)n[nH]c2c1	0
317	C52813773	S(c1c(C)cc(N)cn1)c1n[nH]cn1	0
318	C27028316	S=C1N(NCc2ccc(O)cc2)C(C)=N[N-]1	0
319	C07601230	S=C1N(NCc2c(C)cccc2)C=N[N-]1	0
320	C01086073	O=[N+]([O-])c1c2NC(=O)Nc2c[n+H]c1	0
321	C40887204	Nc1ccc(-c2nc(-c3[nH]cnn3)sc2)cc1	0
322	C48628234	S=C1NC([C@H]2COCC2)=N[N-]1	0
323	C71777422	S=C1N[C@H](c2occc2)C(C#N)=C(O)N1	0
324	C13303738	O=NC=1C(OC)(OC)CCCC=1NO	0
325	C71462807	O=C(N1CC[N+H2]CC1)c1c(O)c(C)ccc1C	0
326	C19913879	O=Cc1cc(C[N+H]2CCOCC2)c(O)cc1	0
327	C71773815	O=C1C(O)=C(C[N+H]2CCOCC2)OC=C1	0
328	C06557481	s1nc2c3[nH]nnc3ccc2n1	0
329	C70801340	[N+H2](Cc1[nH]c2c(n1)cncc2)C	0
330	C08626636	Clc1nnc(O)c2[nH]cnc12	0
331	C22135293	Oc1c(C[N+H]2CCOCC2)ncnc1C	0
332	C71618367	O[C@H](CO)[C@]1(O)[C@H](c2ccc(C)cc2)[C@]2(O)[C@@H](O)[C@H](c3ccc(C)cc3)[C@@]12O	0
333	C40906154	O(C)c1cc(O)c(CNc2c(C)[nH]nc2C)cc1	0
334	C34445516	ClC1(Cl)[C@@H](O)C(Cl)=C[C@@]1(O)C(=O)[O-]	0
335	C57218752	O=C1C(=O)c2c3[nH]ncc3cc(C)c2N1	0
336	C45814453	O=C1C(=O)Nc2c(N1)cc(O)cc2	0
337	C28760463	S=C1N(CC2=CNC(=O)C=C2)C=CN1	0
338	C04095849	S=C1NC=Nc2[nH]cnc12	0
339	C18127213	S=C1NC(=O)c2[nH]cnc2N1	0
340	C44264492	Nc1cn(Cc2[nH]c3ncccc3n2)nc1	0
341	C19933063	S=C1NC(CCC(=O)[O-])=N[N-]1	0
342	C68573638	FC(F)(F)C(/N=C(/[O-])\Nc1[nH]cnn1)(C(F)(F)F)C(F)(F)F	0

343	C32501662	O=C([O-])Cc1[nH]c(-c2c(O)cccc2)nn1	0
344	C16382917	S([C@@H]1/C(=[N+H]/[O-])/CCCCCC1)c1[nH]cnn1	0
345	C43460814	Brc1cnc2[nH]c(C[C@@](C(=O)[O-])(C(C)C)C)[n+H]c2c1	0
346	C06519447	S(CC(=O)N)c1nc(CC)[nH]n1	0
347	C49474200	O=C1N2[N+H]=CNN=C2N=C(C)C1	0
348	C17173835	O=C([O-])/C=C/C(=O)N[O-]	0
349	C70602579	O=C(OC1CCCCCC1)c1nc(N)[nH]n1	0
350	C37921092	Clc1c(Cl)cc(C(=O)NCc2[nH]cnn2)[nH]1	0
351	C40290664	S(c1c(C[N+H3])c(C)c(C)nn1)c1[nH]cnn1	0
352	C53129360	O=C([O-])C([N+H2][C@@]1(CO)C[C@H](C)CCC1)(C)C	0
353	C45655737	N([C@@H](C)c1nnn[n-]1)[C@@H]1C[N+H](C(C)C)CC1	0
354	C44926888	Oc1cc2c([C@@H]([N+H2][C@H](C)c3nnn[n-]3)CCC2)cc1	0
355	C70841874	O=C([O-])[C@](NC(=O)c1nn[nH]c1)(C)C1CC1	0
356	C54931842	S(c1ncnc2[nH]cnc12)C1C[N+H2]C1	0
357	C50845072	Nc1nc(-c2n[nH]cn2)nc(C)c1	0
358	C70251249	O=C(Nc1ncnc2nc[nH]c12)COC	0
359	C39899630	Cl[C@@H](C(=O)Nc1ncnc2[nH]cnc12)C	0
360	C43569657	S(c1n[nH]cn1)c1[n+H]ccc(N)c1	0
361	C61712998	O=C([O-])CCNC(=O)N1C[C@@H](O)CC1	0
362	C16383208	Brc1c(O)c(CN=Nc2nn[n-]n2)cc(Cl)c1	0
363	C17533368	O=C([O-])[C@](O)(C[N+H](CC)C)C	0
364	C12841401	P(=O)(Oc1c(CC)c(CO)c[n+H]c1C)([O-])[O-]	0
365	C08769510	OCCNc1nc(O)c(C)nn1	0
366	C69948985	S(=O)(=O)(/N=C/1\NOC=C\1)c1nc[nH]c1	0
367	C45220738	S(C[C@@]([N+H3])(C#N)c1ccccc1)c1nc(C)[nH]n1	0
368	C50330349	[N+H2](CCC)[C@@H]1c2sc(-c3[nH]cnn3)nc2CCC1	0
369	C61844291	C([N+H]1Cc2sc(-c3[nH]cnn3)nc2CC1)c1ccccc1	0
370	C61171580	O=C([O-])C([N+H](C)[C@@H]1[C@@H](O)CS(=O)(=O)C1)(C)C	0
371	C69949399	S(=O)(=O)(N[C@H](C#N)C)c1nc(C)[nH]c1	0
372	C13479816	O=C1N(C[N+H]2CCC(C)CC2)N=C(O)C=C1	0
373	C12364126	O=C(C)N1[C@@H](O)[C@@H](O)N(C(=O)C)C1=O	0
374	C02383087	O=CC(O)C=O	0
375	C17957808	S(Sc1[nH]ncn1)c1[nH]cnn1	0
376	C02245250	S=C([O-])CC=1SC(=S)NN=1	0
377	C01239139	S=C1C(C)=N[N-]C(=S)N1	0
378	C01599195	O=C1[N-]C(=O)c2nsnc2[N-]1	0
379	C21300171	[S-]c1c(C(=O)[O-])c([O-])ns1	0
380	103	S(=O)(=O)(Nc1ccc(S(=O)(=O)N)cc1)c1c(C(=O)[O-])scc1	1
381	309	O=C([O-])c1c(NC(=O)c2c(C)cco2)cccc1	1
382	307	O=C([O-])CN1C(=O)[C@H](c2ccccc2)OC1=O	1
383	205	O=C([O-])C(Cc1ccccc1)N1C(=O)c2c(C1=O)cccc2	1
384	101	S(CCC(=O)[O-])c1sc2c(n1)cccc2	1
385	322	O=C(Nc1c(C(=O)[O-])ccs1)C	1

386	306	S(=O)(=O)(N)c1c(C(=O)[O-])cccc1	1
387	212	S(CCC(C(=O)[O-])N1C(=O)c2c(C1=O)ccc(C(=O)[O-])c2)C	1
388	202	O=C([O-])C(Cc1ccccc1)N1C(=O)c2c(C1=O)ccc(C(=O)[O-])c2	1
389	211	O=C([O-])[C@@H](Cc1c2c(ccc1)cccc2)N1C(=O)c2c(C1=O)ccc(C(=O)[O-])c2	1
390	208	O=C([O-])C[C@@H](Cc1c2c(ccc1)cccc2)N1C(=O)c2c(C1=O)ccc(C(=O)[O-])c2	1
391	102	S(=O)(=O)([O-])CCCSc1sc2c(n1)cccc2	1
392	206	O=C([O-])c1cc2C(=O)N(CCc3ccc(O)cc3)C(=O)c2cc1	1
393	304	O=C([O-])[C@@H]([C@@H](O)C)N1C(=O)c2c(C1=O)cccc2	1
394	308	O=C([O-])[C@@H]1CN(c2c(O)cccc2)C(=O)C1	1
395	312	O=C([O-])c1[nH]c(-c2ccccc2)nc1	1
396	317	Clc1ccc(-c2c(C(=O)[O-])c(N)on2)cc1	1
397	320	Sc1[nH]c([C@@H]2[C@H](C(=O)[O-])CCCC2)nn1	1
398	303	O=C1N(c2nnn[n-]2)NC(C)=C1CC	1
399	319	P(=O)([O-])([O-])c1cc2sc(C)[n+H]c2cc1	1

Table S3. Compounds used in the Rac1 VS. Inactives are labelled as “0”, actives as “1”

Lig. #	mol	active
1	O=C(NN=C1C(=O)Nc2c1cccc2)c1c(C)nc(-c2ccccc2)cc1	0
2	Brc1cc2C(=NNC(=O)c3c(C)nc(-c4ccc(Cl)cc4)cc3)C(=O)Nc2cc1	0
3	O=C(C=Cc1cc2OCOc2cc1)c1c(C)nc(-c2ccccc2)cc1	0
4	O=C(NN=C1C(=O)Nc2c1cccc2)c1c(C)nc(-c2ccc(C)cc2)cc1	0
5	Clc1ccc(-c2ncc(C(=O)NN=C3C(=O)Nc4c3cccc4)cc2)cc1	0
6	S(=O)(=O)(N1CCCCC1)c1ccc(NC(=O)c2[nH]nc(-c3c(O)c(C)ccc3)c2)cc1	0
7	S(=O)(=O)(N1C[C@@H](C)CCC1)c1ccc(NC(=O)c2[nH]nc(-c3c(O)ccc(C)c3)c2)cc1	0
8	Brc1ccc(C2N(c3ccc(S(=O)(=O)N)cc3)N=C(c3cc(O)ccc3)C2)cc1	0
9	S(=O)(=O)(N)c1ccc(N2C(c3ccc(O)cc3)CC(c3sccc3)=N2)cc1	0
10	Clc1ccc(-c2nc(C)c(C(=O)NN=Cc3cc4OCOc4cc3)cc2)cc1	0
11	S(=O)(=O)(N)c1ccc(N2C(c3cc(C)ccc3)CC(c3ccccc3)=N2)cc1	0
12	S(=O)(=O)(N)c1ccc(N2C(c3ccc(OC)cc3)CC(c3ccccc3)=N2)cc1	0
13	S(=O)(=O)(N)c1ccc(N2C(c3ccc(O)cc3)CC(c3cscc3)=N2)cc1	0
14	S(=O)(=O)(Cc1ncccn1)c1ccc(NC(=S)NC(=O)COc2ccccc2)cc1	0
15	[N+][C@H](C(NN=Cc1c2nccnc2ccc1)=C)Cc1ccccc1	0
16	S(=O)(=O)(Nc1ccccc1)c1ccc(NC(=S)NC(=O)COc2ccccc2)cc1	0
17	S(=O)(=O)(NCCCCCC[N+])c1c2c(ccc1)cccc2	0
18	Clc1c2c(c(S(=O)(=O)NCCCCCC[N+])ccc2)ccc1	0
19	Brc1cc(C2N(c3ccc(S(=O)(=O)N)cc3)N=C(c3ccccc3)C2)ccc1	0
20	Brc1cc(Cl)c(OCC(=O)NC(=S)Nc2ccc(S(=O)(=O)Nc3ncccn3)cc2)cc1	0
21	S(=O)(=O)(N)c1ccc(N2C(c3ccc(C)cc3)CC(c3ccccc3)=N2)cc1	0
22	Brc1cc(C2N(c3ccc(S(=O)(=O)N)cc3)N=C(c3ccccc3)C2)ccc1	0
23	S(=O)(=O)(N)c1ccc(N2C(c3ccccc3)CC(c3ccccc3)=N2)cc1	0
24	S(=O)(=O)(N)c1ccc(-n2c(-c3ccc(OC)cc3)cc(-c3ccccc3)n2)cc1	0
25	S(=O)(=O)(N)c1ccc(-n2c(-c3ccc(OC)cc3)cc(-c3cc(OC)ccc3)n2)cc1	0
26	O=C(NN=Cc1cc2nccnc2cc1)C([N+])Cc1c2c([nH]c1)cccc2	0
27	Clc1cc(C2N(c3ccc(S(=O)(=O)N)cc3)N=C(c3ccccc3)C2)ccc1	0
28	S(=O)(=O)(N)c1ccc(N2C(c3ccc(OC)cc3)CC(c3c(OC)cccc3)=N2)cc1	0

29	S(=O)(=O)(N)c1ccc(N2C(c3cc(OC)ccc3)CC(c3ccccc3)=N2)cc1	0
30	O=C(NN=Cc1cc2nccnc2cc1)C([N+])C(C)C	0
31	O=C1Oc2c(cccc2)C=2OC(N)=C(C#N)C(c3ccccc3)C1=2	0
32	S(=O)(=O)(N1CCC(NC(=O)c2[nH]nc(-c3ccc(C)cc3)c2)CC1)c1ccc(OC)cc1	0
33	S(=O)(=O)(N1[C@H](CC)CCCC1)c1ccc(NC(=O)c2[nH]nc(-c3c(O)cccc3)c2)cc1	0
34	Clc1ccc(C2N(c3ccc(S(=O)(=O)N)cc3)N=C(c3ccccc3)C2)cc1	0
35	O=C([O-])[C@@H](NC(=O)c1cc2nccnc2cc1)Cc1ccccc1	0
36	S(=O)(=O)(N1CCCC1)c1c(OC)ccc(C(=O)Nc2c3c(c(N4CCCCC4)cc2)ccnc3)c1	0
37	S(=O)(=O)(N1CCCCCC1)c1cc(NC(=O)c2[nH]nc(-c3ccccc3)c2)c(OC)cc1	0
38	O=C(NCCCc1[nH]c2c(n1)cc1c(c2)cccc1)c1cc2nccnc2cc1	0
39	S(=O)(=O)(Nc1ccc(NC(=O)c2ccc(OCc3c(C)onc3C)cc2)cc1)c1ccc(F)cc1	0
40	O=C1Oc2c(cccc2)C2OC(N)=C(C#N)C(c3ccc(O)cc3)C12	0
41	O=C(NN=Cc1cc2c(cc(O)cc2)cc1)Cc1c2c([nH]c1)cccc2	0
42	O=[N+]([O-])c1cc([C@H]2C(C#N)=C(N)OC=3c4c(OC(=O)C2=3)cccc4)ccc1	0
43	N(C(=C)CCCc1c2c([nH]c1)cccc2)c1cc2c(nccc2)cc1	0
44	Brc1ccc(C2N(c3ccc(S(=O)(=O)N)cc3)N=C(c3ccccc3)C2)cc1	0
45	N(CCC[N+]1CCOCC1)c1nc(Nc2cc3scnc3cc2)cc(C)n1	0
46	S(=O)(=O)(N1CCCCCC1)c1ccc(NC(=O)c2[nH]nc(-c3c(O)c(C)c(C)cc3)c2)cc1	0
47	S(=O)(=O)(N)c1ccc(N2C(c3ccc(OC)cc3)CC(C)=N2)cc1	0
48	O=C(N(CCO)C)[C@H]([N+])Cc1c2c([nH]c1)cccc2	0
49	S(=O)(=O)(N1[C@H](C)CCCC1)c1ccc(NC(=O)c2[nH]nc(-c3c(O)cccc3)c2)cc1	0
50	Clc1nc(Nc2cc3ncsc3cc2)cc(C)n1	0
51	[N+](CCCC(Nc1nc(Nc2ccccc2)cc(C)n1)C)(CC)CC	0
52	Clc1cc(NC(=O)CSC=2N(C(=O)NN=2)C2CC2)c([N+](=O)[O-])cc1	0
53	N(CCCC(CC)CC)c1nc(Cc2cc3c([nH]nc3)cc2)ccn1	0
54	S(=O)(=O)(NC)c1c(C)ccc(NC(=O)c2cc(OCc3c(C)onc3C)ccc2)c1	0
55	S(=O)(=O)(N1CCC(C)CC1)c1ccc(NC(=O)c2[nH]nc(-c3c(O)ccc(C)c3)c2)cc1	0
56	S(=O)(=O)(N)c1ccc(N2C(c3cc(C)ccc3)CC(c3ccccc3)=N2)cc1	0
57	S(=O)(=O)(N)c1ccc(N2C(c3ccc(OC)cc3)CC(C(C)(C)C)=N2)cc1	0
58	Clc1nc(Nc2cc3[nH]c(C(F)(F)F)nc3cc2)ccn1	0
59	Clc1nc(Nc2cc3c([nH]nc3)cc2)ccn1	0
60	S(=O)(=O)(N1CCCCC1)c1c(OC)ccc(NC(=O)c2ccc(-n3c(C)cc(C)n3)cc2)c1	0
61	FC(F)(F)c1[nH]c2c(n1)cc(Nc1nc(NCCC[N+](CC)CC)ncc1)cc2	0
62	FC(F)(F)c1[nH]c2c(n1)cc(Nc1nc(NC(CCC[N+](CC)C)C)ncc1)cc2	0
63	S(=O)(=O)(N)c1ccc(N2C(c3ccc(OC)cc3)CC(c3ccc(OC)cc3)=N2)cc1	0
64	O=[N+]([O-])c1ccc(C2[C@@H](C#N)C([N+])OC3c4c(OCC23)cccc4)cc1	0
65	S(=O)(=O)(N1C[C@@H](C)CCC1)c1ccc(NC(=O)c2[nH]nc(-c3c(O)cccc3)c2)cc1	0
66	N(c1nc(NC2CCCC2)ncc1)c1ccccc1	0
67	S(=O)(=O)(N1C[C@H](C)CCC1)c1ccc(NC(=O)c2[nH]nc(-c3c(O)ccc(C)c3)c2)cc1	0
68	S(=O)(=O)(N)c1ccc(N2C(c3ccc(C)cc3)CC(c3ccccc3)=N2)cc1	0
69	S(=O)(=O)(N)c1ccc(N2C(c3ccc(C)cc3)CC(c3ccccc3)=N2)cc1	0
70	Clc1cc(c(O)cc1)-c1n[nH]c(C(=O)Nc2ccc(S(=O)(=O)N3CCCCC3)cc2)c1	0
71	O=C(N(Cc1ccccc1)C)C([N+])Cc1c2c([nH]c1)cccc2	0
72	S(=O)(=O)(N)c1ccc(N2C(c3cc(F)ccc3)CC(c3ccccc3)=N2)cc1	0
73	S(=O)(=O)(N1C[C@H](C)CCC1)c1ccc(NC(=O)c2[nH]nc(-c3c(O)c(C)c(C)cc3)c2)cc1	0
74	S(=O)(=O)(N)c1ccc(N2C(c3c(OC)cccc3)CC(c3ccccc3)=N2)cc1	0
75	S(=O)(=O)(Nc1cc(C(F)(F)F)ccc1)c1ccc(NC(=O)c2cc(OCc3c(C)onc3C)ccc2)cc1	0

76	S(=O)(=O)(N1CCCCC1)c1ccc(NC(=O)c2[nH]nc(-c3c(O)cc(C)cc3)c2)cc1	0
77	S(=O)(=O)(N1CCCCCC1)c1ccc(NC(=O)c2[nH]nc(-c3c(O)ccc(C)c3)c2)cc1	0
78	S(=O)(=O)(N1C[C@@H](C)CCC1)c1ccc(NC(=O)c2[nH]nc(-c3c(O)c(C)c(C)cc3)c2)cc1	0
79	[N+](CCCC(Nc1nc(Nc2cc3c(nccc3)cc2)ccn1)C)(CC)CC	0
80	S(=O)(=O)(N1CCC(C)CC1)c1ccc(NC(=O)c2[nH]nc(-c3c(O)cc(C)c(C)c3)c2)cc1	0
81	Brc1ccc(C2N(c3ccc(S(=O)(=O)N)cc3)N=C(c3ccc(OC)cc3)C2)cc1	0
82	S(=O)(=O)(N1[C@@H](C)CCCC1)c1ccc(NC(=O)c2[nH]nc(-c3c(O)cccc3)c2)cc1	0
83	S(=O)(=O)(N1C[C@@H](C)CCC1)c1ccc(NC(=O)c2[nH]nc(-c3c(O)c(C)cc(C)c3)c2)cc1	0
84	O=C1Oc2c(cccc2)C=2OC(N)=C(C#N)C(c3ccc(C#N)cc3)C1=2	0
85	S(=O)(=O)(N1C[C@H](C)CCC1)c1ccc(NC(=O)c2[nH]nc(-c3c(O)cc(C)cc3)c2)cc1	0
86	S(=O)(=O)(N1CCC(C)CC1)c1ccc(NC(=O)c2[nH]nc(-c3c(O)c(C)cc(C)c3)c2)cc1	0
87	S(=O)(=O)(N)c1ccc(N2C(c3cc(OC)c(OC)cc3)CC(c3ccccc3)=N2)cc1	0
88	S(=O)(=O)(N)c1ccc(N2C(c3ccc(OC)cc3)CC(c3cc(OC)ccc3)=N2)cc1	0
89	O=C(NN=Cc1c2c([nH]c1)cccc2)c1c2c(nc(C)c1)cccc2	0
90	S(=O)(=O)(N1CCCCC1)c1ccc(NC(=O)c2[nH]nc(-c3c(O)c(C)cc(C)c3)c2)cc1	0
91	Clc1cc(c(O)cc1)-c1n[nH]c(C(=O)Nc2ccc(S(=O)(=O)N3CCC(C)CC3)cc2)c1	0
92	S(=O)(=O)(N1CCCCC1)c1ccc(NC(=O)c2[nH]nc(-c3c(O)c(C)c(C)cc3)c2)cc1	0
93	S(=O)(=O)(N1CCCCCC1)c1ccc(NC(=O)c2[nH]nc(-c3c(O)cccc3)c2)cc1	0
94	S(=O)(=O)(N1CCCCC1)c1ccc(NC(=O)c2[nH]nc(-c3c(O)cc(C)c(C)c3)c2)cc1	0
95	S(=O)(=O)(N1C[C@H](C)CCC1)c1ccc(NC(=O)c2[nH]nc(-c3c(O)c(C)cc(C)c3)c2)cc1	0
96	S(=O)(=O)(N1CCCCC1)c1ccc(NC(=O)c2[nH]nc(-c3c(O)ccc(C)c3)c2)cc1	0
97	S(=O)(=O)(N1C[C@H](C)CCC1)c1ccc(NC(=O)c2[nH]nc(-c3c(O)cc(C)c(C)c3)c2)cc1	0
98	[N+](CCCNc1nc(Nc2cc3scnc3cc2)cc(C)n1)(C)C	0
99	S(=O)(=O)(N1CCCCCC1)c1ccc(NC(=O)c2[nH]nc(-c3c(O)cc(C)cc3)c2)cc1	0
100	S(=O)(=O)(N1C[C@@H](C)CCC1)c1ccc(NC(=O)c2[nH]nc(-c3c(O)cc(C)c(C)c3)c2)cc1	0
101	Fc1c([N+](=O)[O-])cc(NCC(=O)Nc2ccc(N3CCOCC3)cc2)cc1	0
102	S(=O)(=O)(N1[C@@H](CC)CCCC1)c1ccc(NC(=O)c2[nH]nc(-c3c(O)cccc3)c2)cc1	0
103	S(=O)(=O)(N1CCCCC1)c1ccc(NC(=O)c2[nH]nc(-c3c(O)cccc3)c2)cc1	0
104	Clc1ccc(-c2n[nH]c(C(=O)NC3CCN(S(=O)(=O)c4ccc(OC)cc4)CC3)c2)cc1	0
105	S(=O)(=O)(N1CCC(C)CC1)c1ccc(NC(=O)c2[nH]nc(-c3c(O)c(C)c(C)cc3)c2)cc1	0
106	S(=O)(=O)(Nc1c(F)cccc1)c1cc(NC(=O)c2ccc(OCc3c(C)onc3C)cc2)ccc1	0
107	S(=O)(=O)(N)c1c(C)ccc(NC(=O)c2cc(OCc3c(C)onc3C)ccc2)c1	1
108	S(=O)(=O)(Nc1ccc(OC)cc1)c1c(N2CCCC2)ccc(NC(=O)c2n[nH]c3c2cccc3)c1	1
109	S(=O)(=O)(N1CCCCC1)c1c(OC)ccc(NC(=O)c2n[nH]c3c2cccc3)c1	1
110	S(=O)(=O)(NC)c1c(C)ccc(NC(=O)Cc2ccc(OCc3c(C)onc3C)cc2)c1	1
111	S(=O)(=O)(N1C[C@H](C)CCC1)c1ccc(NC(=O)c2[nH]nc(-c3c(O)cccc3)c2)cc1	1
112	S(=O)(=O)(N1CCCCCC1)c1ccc(NC(=O)c2[nH]nc(-c3c(O)cc(C)c(C)c3)c2)cc1	1
113	S(=O)(=O)(N1CCC(C)CC1)c1ccc(NC(=O)c2[nH]nc(-c3c(O)cccc3)c2)cc1	1
114	S(=O)(=O)(N1CCC(C)CC1)c1ccc(NC(=O)c2[nH]nc(-c3c(O)c(C)c(C)cc3)c2)cc1	1
115	S(=O)(=O)(N1CCCCC1)c1ccc(NC(=O)c2[nH]nc(-c3c(O)c(C)c(C)cc3)c2)cc1	1
116	S(=O)(=O)(N1CCCCC1)c1ccc(NC(=O)c2[nH]nc(-c3c(O)cccc3)c2)cc1	1

Table S4. Predicted binding energies (kcal/mol) obtained from the analysis of the 1st ns of MD simulations run on GPU cards for the penicillopepsin system (RUN 1).

		**Nwat = 0**		**Nwat = 10**		**Nwat = 20**		**Nwat = 30**		**Nwat = 40**		**Nwat = 50**	
**Cmp**	**ΔG exp**	**ΔG_{bind}**	**σ**	**ΔG_{bind}**	**σ**	**ΔG_{bind}**	**σ**	**ΔG_{bind}**	**σ**	**ΔG_{bind}**	**σ**	**ΔG_{bind}**	**σ**
1APT	-12.83	-52.6	6.2	-73.4	7.1	-84.3	7.6	-92.5	6.7	-97.3	6.5	-98.9	6.3
1APU	-10.51	-32.3	3.7	-43.0	5.4	-51.2	6.2	-58.2	5.7	-61.6	5.5	-63.3	5.5
1APV	-12.27	-42.5	3.5	-50.0	5.7	-56.0	6.3	-64.4	6.5	-70.9	6.5	-74.0	6.5
1APW	-10.91	-30.0	4.3	-38.4	6.5	-46.7	6.5	-57.1	6.5	-64.4	7.1	-68.2	7.3
2WEA	-8.37	-36.7	7.1	-21.9	7.4	-30.0	9.2	-40.7	9.0	-47.5	8.5	-49.9	8.8
2WEB	-7.03	-12.5	5.4	-16.0	7.1	-25.1	7.1	-32.8	7.2	-35.4	7.6	-36.0	7.9
2WEC	-6.80	-39.2	6.0	-28.2	8.7	-34.2	9.7	-44.1	10.5	-52.4	11.0	-57.2	11.1

Table S5. Predicted binding energies (kcal/mol) obtained from the analysis of the 1st ns of MD simulations run on GPU cards for the penicillopepsin system (RUN 2).

		**Nwat = 0**		**Nwat = 10**		**Nwat = 20**		**Nwat = 30**		**Nwat = 40**		**Nwat = 50**	
**Cmp**	**ΔG exp**	**ΔG_{bind}**	**σ**	**ΔG_{bind}**	**σ**	**ΔG_{bind}**	**σ**	**ΔG_{bind}**	**σ**	**ΔG_{bind}**	**σ**	**ΔG_{bind}**	**σ**
1APT	-12.83	-55.7	5.3	-76.5	6.4	-87.0	6.9	-95.2	6.9	-100.1	7.4	-102.0	7.6
1APU	-10.51	-41.3	3.3	-47.8	6.0	-56.2	6.4	-63.5	7.0	-67.2	8.2	-68.1	8.8
1APV	-12.27	-40.8	3.8	-48.8	7.1	-54.8	8.1	-62.1	6.8	-67.3	6.9	-70.8	7.3
1APW	-10.91	-24.4	5.9	-31.1	6.8	-37.7	6.9	-47.5	7.3	-55.2	7.1	-59.7	7.2
2WEA	-8.37	-40.7	6.5	-20.4	7.3	-23.8	6.9	-33.4	7.0	-40.4	7.6	-42.6	7.5
2WEB	-7.03	-10.7	5.1	-14.2	7.4	-22.3	8.5	-29.4	8.4	-32.3	8.1	-32.6	8.4
2WEC	-6.80	-38.6	6.7	-28.5	8.0	-32.1	8.0	-40.4	8.1	-48.0	8.5	-51.8	9.1

Table S6. Predicted binding energies (kcal/mol) obtained from the analysis of the 1st ns of MD simulations run on GPU cards for the penicillopepsin system (RUN 3).

		**Nwat = 0**		**Nwat = 10**		**Nwat = 20**		**Nwat = 30**		**Nwat = 40**		**Nwat = 50**	
**Cmp**	**ΔG exp**	**ΔG_{bind}**	**σ**	**ΔG_{bind}**	**σ**	**ΔG_{bind}**	**σ**	**ΔG_{bind}**	**σ**	**ΔG_{bind}**	**σ**	**ΔG_{bind}**	**σ**
1APT	-12.83	-52.3	5.2	-71.9	6.8	-80.1	7.3	-88.1	6.8	-92.9	6.9	-94.8	6.9
1APU	-10.51	-31.5	3.9	-36.2	6.0	-41.6	6.7	-48.0	7.0	-51.1	7.4	-51.6	7.7
1APV	-12.27	-37.4	4.6	-50.4	6.4	-57.9	6.5	-66.6	7.6	-73.8	8.4	-78.2	7.8
1APW	-10.91	-21.2	5.1	-36.6	7.6	-46.2	8.4	-56.9	7.8	-65.7	8.1	-70.3	8.5
2WEA	-8.37	-39.5	6.1	-24.5	7.2	-31.7	7.5	-42.3	7.6	-50.0	8.6	-53.6	9.5
2WEB	-7.03	-17.8	6.4	-21.8	6.7	-30.2	6.8	-35.8	7.1	-37.2	7.1	-37.1	7.1
2WEC	-6.80	-38.8	5.9	-27.1	7.8	-29.6	8.3	-37.5	8.7	-45.5	8.7	-50.7	9.2

Table S7. Predicted binding energies (kcal/mol) obtained from the analysis of the 1st ns of MD simulations run on HPC for the penicillopepsin system (RUN 1).

		**Nwat = 0**		**Nwat = 10**		**Nwat = 20**		**Nwat = 30**		**Nwat = 40**		**Nwat = 50**	
**Cmp**	**ΔG exp**	**ΔG_{bind}**	**Σ**	**ΔG_{bind}**	**σ**	**ΔG_{bind}**	**σ**	**ΔG_{bind}**	**σ**	**ΔG_{bind}**	**σ**	**ΔG_{bind}**	**σ**
1APT	-12.83	-46.7	7.0	-66.1	9.1	-77.7	8.3	-87.4	7.9	-92.7	8.1	-94.9	8.1
1APU	-10.51	-33.7	6.3	-50.5	6.3	-57.6	6.4	-64.8	7.2	-70.3	9.0	-72.4	10.5
1APV	-12.27	-42.7	4.4	-48.0	5.3	-54.6	5.6	-62.6	6.0	-69.4	6.0	-73.9	5.7
1APW	-10.91	-26.4	4.6	-35.0	6.0	-43.6	6.0	-53.6	5.7	-60.4	6.0	-64.0	6.0
2WEA	-8.37	-28.3	8.0	-11.2	9.5	-17.2	9.6	-27.5	10.1	-37.7	9.2	-43.5	8.6
2WEB	-7.03	-13.0	4.6	-14.2	7.6	-23.8	7.3	-32.6	7.6	-37.2	8.0	-38.3	8.5
2WEC	-6.80	-47.3	5.6	-38.5	6.6	-42.3	6.9	-46.9	6.9	-50.3	7.0	-51.4	7.5

Table S8. Predicted binding energies (kcal/mol) obtained from the analysis of the 1st ns of MD simulations run on HPC for the penicillopepsin system (RUN 2).

		**Nwat = 0**		**Nwat = 10**		**Nwat = 20**		**Nwat = 30**		**Nwat = 40**		**Nwat = 50**	
**Cmp**	**ΔG exp**	**ΔG_{bind}**	**σ**	**ΔG_{bind}**	**σ**	**ΔG_{bind}**	**σ**	**ΔG_{bind}**	**σ**	**ΔG_{bind}**	**σ**	**ΔG_{bind}**	**σ**
1APT	-12.83	-48.3	5.0	-66.6	7.1	-73.3	8.2	-80.5	7.9	-86.0	7.9	-88.3	7.7
1APU	-10.51	-28.9	3.5	-43.0	5.9	-50.0	6.3	-55.2	6.5	-58.7	6.9	-59.7	6.7
1APV	-12.27	-35.2	8.1	-52.0	6.5	-59.0	6.8	-67.1	7.0	-74.8	7.2	-79.4	7.6
1APW	-10.91	-26.8	4.2	-37.5	7.0	-42.3	7.2	-48.8	7.6	-54.2	8.5	-55.6	9.6
2WEA	-8.37	-33.2	5.9	-20.2	5.9	-27.7	6.7	-37.7	7.2	-44.3	7.1	-46.0	7.2
2WEB	-7.03	-12.1	5.2	-16.6	5.8	-26.8	6.6	-33.1	6.8	-34.7	6.6	-35.0	6.6
2WEC	-6.80	-29.1	7.5	-16.4	8.5	-20.8	9.4	-30.4	9.4	-39.1	10.1	-45.1	10.8

Table S9. Predicted binding energies (kcal/mol) obtained from the analysis of the 1st ns of MD simulations run on HPC for the penicillopepsin system (RUN 3).

		**Nwat = 0**		**Nwat = 10**		**Nwat = 20**		**Nwat = 30**		**Nwat = 40**		**Nwat = 50**	
**Cmp**	**ΔG exp**	**ΔG_{bind}**	**σ**	**ΔG_{bind}**	**σ**	**ΔG_{bind}**	**σ**	**ΔG_{bind}**	**σ**	**ΔG_{bind}**	**σ**	**ΔG_{bind}**	**σ**
1APT	-12.83	-50.8	4.9	-67.5	6.4	-75.7	7.2	-83.3	6.5	-88.2	6.3	-90.2	6.4
1APU	-10.51	-34.6	2.9	-50.5	6.1	-57.7	6.5	-64.2	7.5	-68.2	8.5	-69.4	9.1
1APV	-12.27	-37.8	5.8	-51.1	9.7	-57.7	8.8	-65.9	6.6	-72.6	7.0	-76.4	8.0
1APW	-10.91	-30.7	4.1	-43.5	6.6	-52.1	7.0	-62.7	8.0	-70.1	8.0	-74.1	8.1
2WEA	-8.37	-39.2	5.5	-19.1	7.7	-24.3	8.6	-34.1	8.6	-40.2	8.8	-42.1	9.3
2WEB	-7.03	-12.0	4.7	-14.9	6.0	-24.2	6.4	-31.1	6.9	-33.3	6.8	-33.6	6.8
2WEC	-6.80	-47.1	9.6	-39.9	10.3	-43.2	10.9	-50.2	10.3	-56.8	10.9	-59.9	10.3

Table S10. Predicted binding energies (kcal/mol) obtained from the analysis of the 4th ns of MD simulations run on GPU cards for the penicillopepsin system (RUN 1).

		Nwat = 0		Nwat = 10		Nwat = 20		Nwat = 30		Nwat = 40		Nwat = 50	
**Cmp**	**ΔG exp**	**ΔG_{bind}**	**σ**	**ΔG_{bind}**	**σ**	**ΔG_{bind}**	**σ**	**ΔG_{bind}**	**σ**	**ΔG_{bind}**	**σ**	**ΔG_{bind}**	**σ**
1APT	-12.83	-48.8	5.0	-66.7	7.8	-78.8	8.3	-87.1	7.4	-91.8	7.1	-93.6	6.8
1APU	-10.51	-32.0	3.5	-43.7	5.5	-51.8	5.4	-59.5	6.6	-64.8	6.4	-66.7	6.3
1APV	-12.27	-43.1	3.6	-50.1	4.8	-54.7	5.3	-62.9	6.9	-70.3	7.3	-75.0	7.3
1APW	-10.91	-22.8	4.7	-36.9	6.9	-39.3	7.3	-45.0	7.3	-51.4	7.5	-56.3	7.2
2WEA	-8.37	-42.4	4.6	-31.4	8.5	-39.0	8.2	-45.9	8.3	-48.6	8.5	-49.3	9.0
2WEB	-7.03	-18.8	5.1	-15.2	5.9	-24.5	6.3	-33.6	6.2	-36.9	6.2	-38.3	6.3
2WEC	-6.80	-41.7	6.0	-28.3	8.4	-34.7	8.8	-44.6	8.2	-51.7	7.6	-55.8	7.6

Table S11. Predicted binding energies (kcal/mol) obtained from the analysis of the 4th ns of MD simulations run on GPU cards for the penicillopepsin system (RUN 2).

		Nwat = 0		Nwat = 10		Nwat = 20		Nwat = 30		Nwat = 40		Nwat = 50	
**Cmp**	**ΔG exp**	**ΔG_{bind}**	**σ**	**ΔG_{bind}**	**σ**	**ΔG_{bind}**	**σ**	**ΔG_{bind}**	**σ**	**ΔG_{bind}**	**σ**	**ΔG_{bind}**	**σ**
1APT	-12.83	-42.8	4.2	-70.8	6.8	-80.5	7.1	-91.5	7.5	-100.1	7.5	-105.4	7.5
1APU	-10.51	-31.9	3.1	-40.9	5.2	-47.4	6.2	-58.9	6.8	-69.8	6.4	-77.5	6.7
1APV	-12.27	-39.3	4.5	-46.9	6.2	-49.9	6.8	-56.1	5.8	-61.3	7.0	-64.2	6.5
1APW	-10.91	-22.8	3.0	-41.6	5.8	-46.8	7.0	-55.3	6.5	-64.2	7.1	-70.2	7.7
2WEA	-8.37	-31.3	5.7	-17.7	8.3	-20.1	7.6	-30.6	8.1	-38.0	7.8	-40.8	8.2
2WEB	-7.03	-13.4	4.8	-9.1	6.2	-17.9	6.9	-27.2	7.6	-31.1	7.6	-32.3	7.6
2WEC	-6.80	-41.2	7.9	-26.0	8.9	-30.3	8.9	-40.9	8.1	-51.6	8.0	-59.8	8.0

Table S12. Predicted binding energies (kcal/mol) obtained from the analysis of the 4th ns of MD simulations run on GPU cards for the penicillopepsin system (RUN 3).

		Nwat = 0		Nwat = 10		Nwat = 20		Nwat = 30		Nwat = 40		Nwat = 50	
**Cmp**	**ΔG exp**	**ΔG_{bind}**	**σ**	**ΔG_{bind}**	**σ**	**ΔG_{bind}**	**σ**	**ΔG_{bind}**	**σ**	**ΔG_{bind}**	**σ**	**ΔG_{bind}**	**σ**
1APT	-12.83	-50.5	4.5	-67.5	6.1	-74.7	6.6	-82.4	6.6	-88.5	6.3	-91.8	6.3
1APU	-10.51	-35.2	3.8	-39.5	5.3	-46.3	5.5	-52.8	5.7	-55.4	5.9	-55.5	6.1
1APV	-12.27	-21.3	2.6	-37.7	4.6	-49.1	4.0	-56.5	6.4	-65.5	7.2	-70.3	6.8
1APW	-10.91	-41.1	4.3	-52.4	6.4	-60.8	7.2	-69.4	6.8	-76.2	6.9	-80.0	7.7
2WEA	-8.37	-46.9	5.0	-33.8	6.8	-40.7	7.8	-51.1	7.9	-58.6	8.1	-61.4	7.8
2WEB	-7.03	-14.9	4.4	-14.6	6.4	-24.0	6.9	-33.0	6.8	-38.0	6.4	-39.2	6.7
2WEC	-6.80	-38.8	5.9	-27.1	7.8	-29.6	8.3	-37.5	8.7	-45.5	8.7	-50.7	9.2

Table S13. Predicted binding energies (kcal/mol) obtained from the analysis of the 4th ns of MD simulations run on HPC for the penicillopepsin system (RUN 1).

		**Nwat = 0**		**Nwat = 10**		**Nwat = 20**		**Nwat = 30**		**Nwat = 40**		**Nwat = 50**	
**Cmp**	**ΔG exp**	**ΔG_{bind}**	**σ**	**ΔG_{bind}**	**σ**	**ΔG_{bind}**	**σ**	**ΔG_{bind}**	**σ**	**ΔG_{bind}**	**σ**	**ΔG_{bind}**	**σ**
1APT	-12.83	-54.6	5.1	-70.5	6.9	-78.4	7.6	-87.6	7.4	-94.3	7.4	-97.8	7.5
1APU	-10.51	-26.1	4.0	-44.1	6.8	-51.7	7.2	-61.0	7.3	-70.0	7.7	-75.6	7.8
1APV	-12.27	-37.0	4.2	-51.4	5.5	-56.1	5.6	-64.5	6.2	-72.0	6.5	-77.4	6.7
1APW	-10.91	-22.8	4.6	-32.8	6.2	-41.6	6.5	-52.6	6.7	-59.6	7.1	-63.1	7.7
2WEA	-8.37	-21.5	3.7	-3.1	7.5	-8.2	7.5	-19.4	7.9	-28.3	8.1	-34.0	8.3
2WEB	-7.03	-5.9	5.5	-3.2	6.2	-11.4	6.4	-20.2	6.6	-25.3	6.8	-27.4	6.7
2WEC	-6.80	-45.1	5.3	-38.1	8.4	-43.9	8.6	-52.6	8.4	-58.0	8.9	-59.7	9.1

Table S14. Predicted binding energies (kcal/mol) obtained from the analysis of the 4th ns of MD simulations run on HPC for the penicillopepsin system (RUN 2).

		**Nwat = 0**		**Nwat = 10**		**Nwat = 20**		**Nwat = 30**		**Nwat = 40**		**Nwat = 50**	
**Cmp**	**ΔG exp**	**ΔG_{bind}**	**σ**	**ΔG_{bind}**	**σ**	**ΔG_{bind}**	**σ**	**ΔG_{bind}**	**σ**	**ΔG_{bind}**	**σ**	**ΔG_{bind}**	**σ**
APT	-12.83	-35.5	3.9	-65.6	7.0	-71.8	7.1	-79.7	7.8	-86.2	8.3	-89.7	8.3
APU	-10.51	-28.9	4.8	-42.3	5.4	-47.8	5.5	-54.4	5.5	-60.1	5.7	-63.1	5.6
APV	-12.27	-20.8	3.8	-43.6	5.8	-49.6	6.4	-58.3	6.5	-67.3	7.2	-74.0	6.2
APW	-10.91	-19.4	4.3	-39.2	6.5	-46.0	7.2	-55.4	6.2	-64.2	7.5	-69.6	8.1
WEA	-8.37	-49.3	4.6	-32.1	7.6	-38.8	8.4	-49.1	8.6	-56.3	8.6	-60.7	8.3
WEB	-7.03	-10.5	5.6	-9.8	7.0	-17.9	7.1	-27.2	7.6	-31.6	7.6	-32.5	7.6
WEC	-6.80	-32.6	6.6	-19.6	8.7	-25.7	10.6	-36.3	10.5	-44.6	11.2	-48.3	11.6

Table S15. Predicted binding energies (kcal/mol) obtained from the analysis of the 4th ns of MD simulations run on HPC for the penicillopepsin system (RUN 3).

		**Nwat = 0**		**Nwat = 10**		**Nwat = 20**		**Nwat = 30**		**Nwat = 40**		**Nwat = 50**	
**Cmp**	**ΔG exp**	**ΔG_{bind}**	**σ**	**ΔG_{bind}**	**σ**	**ΔG_{bind}**	**σ**	**ΔG_{bind}**	**σ**	**ΔG_{bind}**	**σ**	**ΔG_{bind}**	**σ**
1APT	-12.83	-32.9	5.6	-53.4	6.8	-64.1	7.7	-74.4	7.2	-81.4	6.3	-85.3	6.2
1APU	-10.51	-26.8	2.9	-50.1	5.6	-58.7	6.0	-69.6	6.6	-78.4	6.8	-83.2	6.8
1APV	-12.27	-22.3	4.2	-46.6	6.7	-51.9	7.6	-59.5	7.3	-67.1	7.3	-73.0	7.0
1APW	-10.91	-24.3	4.4	-41.1	7.3	-50.2	7.3	-60.0	7.0	-68.1	7.0	-73.7	7.0
2WEA	-8.37	-41.9	4.0	-27.8	6.0	-33.8	6.6	-44.2	6.8	-51.8	7.3	-54.5	6.7
2WEB	-7.03	-15.7	5.1	-10.0	6.0	-18.5	7.0	-28.2	6.9	-33.3	6.6	-34.1	6.9
2WEC	-6.80	-28.5	4.7	-17.5	8.4	-19.5	8.4	-31.9	8.6	-45.0	8.7	-55.7	9.3

Table S16. Predicted binding energies (kcal/mol) obtained from the analysis of the 1st ns of MD simulations run on GPU cards for the HIV1-protease system (RUN 1).

		Nwat = 0		Nwat = 10		Nwat = 20		Nwat = 30		Nwat = 40		Nwat = 50		Nwat = 60		Nwat = 70	
**Cmp**	**ln(k$_i$)**	**ΔG$_{bind}$**	**σ**	**ΔG$_{bind}$**	**σ**	**ΔG$_{bind}$**	**σ**	**ΔG$_{bind}$**	**σ**	**ΔG$_{bind}$**	**σ**	**ΔG$_{bind}$**	**σ**	**ΔG$_{bind}$**	**σ**	**ΔG$_{bind}$**	**σ**
3NDW	-2.81	-54.2	4.4	-63.5	5.8	-69.3	6.1	-76.4	6.0	-80.8	6.0	-83.1	6.2	-84.4	6.2	-85.0	6.1
3NDX	-2.81	-55.6	4.5	-65.1	5.9	-71.4	6.4	-78.0	6.6	-81.4	6.3	-83.1	6.3	-84.0	6.3	-84.2	6.3
3NU3	-1.90	-46.6	4.1	-59.7	5.7	-67.6	5.8	-68.9	5.7	-69.1	5.7	-69.1	5.8	-59.7	5.7	-67.6	5.8
3NU4	0.41	-45.9	6.1	-62.4	5.4	-69.1	5.4	-70.0	5.6	-70.1	5.7	-70.1	5.7	-62.4	5.4	-69.1	5.4
3NU5	1.50	-51.3	4.7	-62.1	5.6	-67.5	5.6	-68.4	5.5	-68.4	5.7	-68.2	5.7	-62.1	5.6	-67.5	5.6
3NU6	-0.69	-48.7	6.0	-64.3	5.5	-69.4	6.0	-69.8	5.9	-69.8	6.0	-69.8	5.9	-64.3	5.5	-69.4	6.0
3NU9	-0.11	-51.4	4.4	-62.4	4.9	-67.6	5.0	-68.1	5.1	-67.9	5.2	-67.9	5.1	-62.4	4.9	-67.6	5.0
3NUJ	-0.89	-50.2	5.0	-67.1	6.0	-74.6	5.2	-76.4	5.0	-76.6	5.1	-76.5	5.0	-67.1	6.0	-74.6	5.2
3NUO	-1.83	-59.0	4.7	-72.5	5.3	-76.2	4.9	-76.7	4.9	-76.8	4.9	-77.1	5.0	-72.5	5.3	-76.2	4.9

Table S17. Predicted binding energies (kcal/mol) obtained from the analysis of the 1st ns of MD simulations run on GPU cards for the HIV1-protease system (RUN 2).

		Nwat = 0		Nwat = 10		Nwat = 20		Nwat = 30		Nwat = 40		Nwat = 50		Nwat = 60		Nwat = 70	
**Cmp**	**ln(k$_i$)**	**ΔG$_{bind}$**	**σ**	**ΔG$_{bind}$**	**σ**	**ΔG$_{bind}$**	**σ**	**ΔG$_{bind}$**	**σ**	**ΔG$_{bind}$**	**σ**	**ΔG$_{bind}$**	**σ**	**ΔG$_{bind}$**	**σ**	**ΔG$_{bind}$**	**σ**
3NDW	-2.81	-54.8	4.7	-64.5	6.0	-71.3	6.2	-77.7	6.8	-80.9	6.6	-82.6	6.6	-83.4	6.6	-83.5	6.7
3NDX	-2.81	-52.2	4.1	-61.3	6.1	-66.5	6.2	-73.5	6.0	-78.1	6.0	-80.6	6.1	-81.8	6.1	-82.3	6.0
3NU3	-1.90	-45.3	5.5	-65.1	6.1	-73.5	6.7	-75.1	6.7	-75.0	6.7	-74.8	6.8	-74.8	6.8	-74.6	6.8
3NU4	0.41	-50.3	5.6	-62.3	6.5	-67.2	6.3	-67.9	6.3	-67.9	6.3	-67.8	6.2	-67.8	6.2	-67.7	6.2
3NU5	1.50	-52.0	4.2	-62.9	4.8	-67.8	4.7	-68.4	4.8	-68.4	4.8	-68.3	4.9	-68.2	4.9	-68.1	5.0
3NU6	-0.69	-51.2	4.6	-64.3	5.9	-70.3	6.7	-71.1	6.7	-71.1	6.6	-70.9	6.6	-70.8	6.6	-70.6	6.7
3NU9	-0.11	-47.5	6.2	-64.6	6.4	-69.4	6.7	-69.5	6.4	-69.5	6.4	-69.5	6.5	-69.3	6.5	-69.2	6.6
3NUJ	-0.89	-52.5	4.9	-68.9	6.8	-76.1	5.7	-78.0	5.7	-78.3	5.8	-78.2	5.7	-78.1	5.7	-78.0	5.7
3NUO	-1.83	-56.3	4.4	-71.2	5.2	-75.2	5.4	-75.6	5.5	-75.8	5.6	-76.0	5.7	-76.2	5.7	-76.2	5.7

Table S18. Predicted binding energies (kcal/mol) obtained from the analysis of the 1st ns of MD simulations run on GPU cards for the HIV1-protease system (RUN 3).

		Nwat = 0		Nwat = 10		Nwat = 20		Nwat = 30		Nwat = 40		Nwat = 50		Nwat = 60		Nwat = 70	
**Cmp**	**ln(k$_i$)**	**ΔG$_{bind}$**	**σ**	**ΔG$_{bind}$**	**σ**	**ΔG$_{bind}$**	**σ**	**ΔG$_{bind}$**	**σ**	**ΔG$_{bind}$**	**σ**	**ΔG$_{bind}$**	**σ**	**ΔG$_{bind}$**	**σ**	**ΔG$_{bind}$**	**σ**
3NDW	-2.81	-50.5	3.8	-62.8	5.5	-68.8	5.8	-74.8	6.1	-77.6	6.4	-79.1	6.5	-79.8	6.5	-80.0	6.4
3NDX	-2.81	-52.7	5.0	-63.9	5.7	-69.4	6.1	-76.2	6.3	-80.4	6.2	-82.4	6.1	-83.5	6.0	-84.0	6.0
3NU3	-1.90	-53.0	5.0	-68.6	5.7	-72.4	6.0	-73.0	5.9	-73.3	6.1	-73.4	6.1	-73.4	6.1	-73.4	6.1
3NU4	0.41	-49.7	5.1	-62.1	5.8	-67.1	5.9	-67.7	5.9	-67.6	6.0	-67.7	6.0	-67.6	6.0	-67.5	6.0
3NU5	1.50	-52.9	4.6	-62.9	5.9	-68.7	5.9	-69.5	6.0	-69.5	6.1	-69.4	6.2	-69.2	6.2	-69.0	6.2
3NU6	-0.69	-52.8	3.9	-64.4	5.0	-69.3	5.1	-70.3	5.1	-70.2	5.0	-70.1	5.0	-70.0	5.0	-69.9	5.0
3NU9	-0.11	-52.1	4.8	-64.7	6.2	-69.5	6.1	-70.0	6.1	-70.0	6.1	-70.0	6.1	-69.9	6.1	-69.9	6.1
3NUJ	-0.89	-53.7	5.3	-66.2	6.0	-73.6	5.1	-75.6	4.9	-75.8	4.8	-75.7	4.8	-75.5	4.8	-75.3	4.9
3NUO	-1.83	-57.4	4.8	-69.4	6.1	-74.0	5.9	-74.4	5.9	-74.2	5.8	-74.4	5.8	-74.4	5.8	-74.3	5.8

Table S19. Predicted binding energies (kcal/mol) obtained from the analysis of the 1st ns of MD simulations run on HPC for the HIV1-protease system (RUN 1).

		Nwat = 0		Nwat = 10		Nwat = 20		Nwat = 30		Nwat = 40		Nwat = 50		Nwat = 60		Nwat = 70	
**Cmp**	**ln(k$_i$)**	**ΔG$_{bind}$**	**σ**	**ΔG$_{bind}$**	**σ**	**ΔG$_{bind}$**	**σ**	**ΔG$_{bind}$**	**σ**	**ΔG$_{bind}$**	**σ**	**ΔG$_{bind}$**	**σ**	**ΔG$_{bind}$**	**σ**	**ΔG$_{bind}$**	**σ**
3NDW	-2.81	-59.6	5.0	-66.5	4.8	-73.2	5.4	-77.2	5.5	-79.3	5.7	-80.3	5.7	-80.7	5.8	-80.7	5.8
3NDX	-2.81	-49.3	3.4	-60.0	5.3	-68.9	5.7	-76.9	5.4	-82.6	5.4	-84.7	5.6	-85.6	5.7	-86.0	5.6
3NU3	-1.90	-52.7	5.1	-67.8	5.7	-72.5	5.3	-72.9	5.1	-73.0	5.1	-73.1	5.2	-73.1	5.2	-73.0	5.2
3NU4	0.41	-49.0	4.7	-66.5	5.7	-72.2	5.9	-72.8	6.0	-72.8	6.0	-72.7	6.0	-72.6	6.0	-72.5	6.0
3NU5	1.50	-51.8	4.5	-62.9	5.2	-68.3	4.7	-69.3	4.8	-69.5	4.9	-69.5	4.9	-69.5	4.9	-69.3	5.0
3NU6	-0.69	-41.7	4.0	-61.6	5.0	-68.8	5.0	-69.4	5.1	-69.3	5.1	-69.2	5.2	-69.1	5.2	-68.9	5.2
3NU9	-0.11	-40.4	4.7	-58.5	5.9	-64.6	6.1	-65.4	6.1	-65.5	5.9	-65.6	6.0	-65.7	5.9	-65.6	5.9
3NUJ	-0.89	-54.0	3.7	-69.1	5.1	-74.7	5.0	-75.7	4.9	-75.7	4.9	-75.4	4.9	-75.3	5.0	-75.1	5.0
3NUO	-1.83	-54.7	4.2	-68.9	5.0	-74.3	4.9	-75.0	5.0	-75.0	5.1	-75.0	4.9	-75.1	5.0	-75.0	5.1

Table S20. Predicted binding energies (kcal/mol) obtained from the analysis of the 1st ns of MD simulations run on HPC for the HIV1-protease system (RUN 2).

		Nwat = 0		Nwat = 10		Nwat = 20		Nwat = 30		Nwat = 40		Nwat = 50		Nwat = 60		Nwat = 70	
**Cmp**	**ln(k_i)**	**ΔG_{bind}**	**σ**	**ΔG_{bind}**	**σ**	**ΔG_{bind}**	**σ**	**ΔG_{bind}**	**σ**	**ΔG_{bind}**	**σ**	**ΔG_{bind}**	**σ**	**ΔG_{bind}**	**σ**	**ΔG_{bind}**	**σ**
3NDW	-2.81	-47.3	3.6	-66.5	5.8	-74.3	6.1	-80.4	5.9	-83.5	5.7	-84.9	5.5	-85.5	5.6	-85.6	5.6
3NDX	-2.81	-44.6	3.3	-58.7	7.1	-63.4	6.5	-70.2	6.2	-76.9	6.4	-81.3	6.1	-83.5	6.0	-84.7	6.0
3NU3	-1.90	-49.6	3.8	-69.1	5.3	-75.1	5.2	-76.1	5.3	-76.3	5.4	-76.3	5.4	-76.1	5.4	-76.0	5.4
3NU4	0.41	-49.8	4.7	-66.5	5.6	-71.0	5.7	-71.2	5.6	-71.0	5.7	-70.8	5.7	-70.7	5.7	-70.6	5.7
3NU5	1.50	-52.0	4.7	-62.8	5.4	-67.9	5.5	-68.6	5.3	-68.7	5.3	-68.8	5.3	-68.8	5.3	-68.8	5.3
3NU6	-0.69	-46.0	6.5	-60.2	5.8	-65.9	5.6	-67.0	5.4	-66.9	5.3	-66.7	5.3	-66.6	5.3	-66.5	5.3
3NU9	-0.11	-51.6	4.6	-63.5	5.2	-68.1	4.7	-68.5	4.8	-68.7	4.8	-68.6	4.9	-68.6	4.9	-68.5	5.0
3NUJ	-0.89	-59.1	4.2	-70.2	5.4	-75.7	5.5	-76.9	5.4	-76.9	5.4	-76.8	5.3	-76.7	5.4	-76.6	5.4
3NUO	-1.83	-56.8	5.1	-71.1	6.6	-77.5	6.5	-78.2	6.4	-78.2	6.4	-78.3	6.3	-78.3	6.3	-78.2	6.3

Table S21. Predicted binding energies (kcal/mol) obtained from the analysis of the 1st ns of MD simulations run on HPC for the HIV1-protease system (RUN 3).

		Nwat = 0		Nwat = 10		Nwat = 20		Nwat = 30		Nwat = 40		Nwat = 50		Nwat = 60		Nwat = 70	
**Cmp**	**ln(k_i)**	**ΔG_{bind}**	**σ**	**ΔG_{bind}**	**σ**	**ΔG_{bind}**	**σ**	**ΔG_{bind}**	**σ**	**ΔG_{bind}**	**σ**	**ΔG_{bind}**	**σ**	**ΔG_{bind}**	**σ**	**ΔG_{bind}**	**σ**
3NDW	-2.81	-52.3	4.7	-67.1	5.3	-73.8	5.3	-79.3	5.5	-82.0	5.4	-83.3	5.5	-84.1	5.5	-84.3	5.5
3NDX	-2.81	-48.2	3.6	-61.8	5.9	-68.5	5.9	-76.8	6.2	-82.3	5.8	-84.9	5.8	-86.2	5.6	-86.7	5.7
3NU3	-1.90	-49.2	4.5	-70.4	5.9	-76.0	6.0	-77.0	6.2	-77.3	6.2	-77.3	6.2	-77.3	6.2	-77.2	6.2
3NU4	0.41	-42.5	4.1	-61.4	6.1	-70.0	6.8	-71.8	6.7	-71.6	6.6	-71.5	6.7	-71.3	6.6	-71.0	6.6
3NU5	1.50	-53.9	4.6	-64.3	5.4	-69.7	5.2	-70.8	5.1	-70.8	5.2	-70.7	5.3	-70.6	5.3	-70.5	5.3
3NU6	-0.69	-43.7	4.3	-61.2	6.1	-71.5	5.7	-76.5	5.8	-77.6	6.1	-77.7	5.9	-77.5	5.8	-77.1	5.8
3NU9	-0.11	-47.9	4.3	-61.5	6.6	-67.9	6.2	-69.0	5.7	-69.1	5.7	-69.1	5.7	-69.2	5.8	-69.0	5.9
3NUJ	-0.89	-52.3	3.7	-70.1	5.4	-76.5	5.1	-77.2	4.9	-77.3	4.9	-77.3	4.9	-77.2	5.0	-77.2	5.0
3NUO	-1.83	-56.0	4.3	-68.2	5.3	-73.2	5.2	-74.1	5.3	-74.1	5.3	-74.2	5.3	-74.2	5.4	-74.1	5.5

Table S22. Predicted binding energies (kcal/mol) obtained from the analysis of the 4th ns of MD simulations run on GPU for the HIV1-protease system (RUN 1).

		Nwat = 0		Nwat = 10		Nwat = 20		Nwat = 30		Nwat = 40		Nwat = 50		Nwat = 60		Nwat = 70	
**Cmp**	**ln(k_i)**	**ΔG_{bind}**	**σ**	**ΔG_{bind}**	**σ**	**ΔG_{bind}**	**σ**	**ΔG_{bind}**	**σ**	**ΔG_{bind}**	**σ**	**ΔG_{bind}**	**σ**	**ΔG_{bind}**	**σ**	**ΔG_{bind}**	**σ**
3NDW	-2.81	-61.1	3.9	-70.6	5.3	-77.8	5.1	-84.3	5.2	-87.0	5.2	-88.5	5.5	-89.1	5.6	-89.3	5.6
3NDX	-2.81	-54.1	3.9	-70.9	5.6	-76.4	5.7	-83.6	5.9	-88.1	6.0	-89.9	5.9	-90.9	6.0	-91.5	6.1
3NU3	-1.90	-40.2	3.9	-65.0	6.2	-76.3	6.1	-78.9	6.0	-79.6	6.1	-79.9	6.2	-79.9	6.2	-79.8	6.3
3NU4	0.41	-41.9	3.6	-60.8	5.2	-71.5	5.7	-74.6	5.4	-75.4	5.2	-75.9	5.2	-75.9	5.2	-75.9	5.2
3NU5	1.50	-52.6	4.3	-65.0	4.9	-69.1	4.8	-69.5	4.9	-69.7	5.0	-69.9	4.9	-69.9	5.0	-69.9	5.0
3NU6	-0.69	-33.5	3.1	-44.8	5.1	-55.0	4.9	-63.6	4.8	-67.1	4.8	-68.0	4.6	-67.6	4.5	-67.0	4.5
3NU9	-0.11	-48.4	3.8	-68.7	5.2	-73.8	5.0	-74.0	5.2	-73.8	5.2	-73.8	5.2	-73.9	5.3	-73.9	5.3
3NUJ	-0.89	-49.7	3.6	-68.1	5.3	-76.4	5.0	-78.3	5.3	-78.4	5.4	-78.3	5.4	-78.2	5.4	-78.1	5.4
3NUO	-1.83	-57.3	3.7	-71.7	4.9	-76.3	5.1	-76.9	4.9	-76.9	4.9	-77.0	4.9	-77.1	4.9	-77.1	4.9

Table S23. Predicted binding energies (kcal/mol) obtained from the analysis of the 4th ns of MD simulations run on GPU for the HIV1-protease system (RUN 2).

		Nwat = 0		Nwat = 10		Nwat = 20		Nwat = 30		Nwat = 40		Nwat = 50		Nwat = 60		Nwat = 70	
**Cmp**	**ln(k_i)**	**ΔG_{bind}**	**σ**	**ΔG_{bind}**	**σ**	**ΔG_{bind}**	**σ**	**ΔG_{bind}**	**σ**	**ΔG_{bind}**	**σ**	**ΔG_{bind}**	**σ**	**ΔG_{bind}**	**σ**	**ΔG_{bind}**	**σ**
3NDW	-2.81	-47.3	3.0	-64.0	5.1	-74.2	6.2	-83.1	6.3	-88.0	6.0	-89.9	6.0	-90.9	5.9	-91.3	5.9
3NDX	-2.81	-44.6	3.6	-56.8	5.5	-63.4	5.8	-70.4	6.1	-75.8	6.2	-78.1	6.2	-78.8	6.2	-79.4	6.3
3NU3	-1.90	-48.7	3.9	-71.4	5.4	-78.8	5.6	-79.7	5.5	-80.0	5.4	-80.1	5.4	-80.2	5.4	-80.2	5.4
3NU4	0.41	-40.9	3.6	-62.3	5.9	-72.2	6.0	-74.2	5.9	-74.9	6.2	-74.9	6.2	-74.7	6.3	-74.5	6.3
3NU5	1.50	-55.5	3.6	-66.6	4.1	-71.1	4.2	-71.7	4.3	-71.8	4.3	-71.8	4.2	-71.8	4.3	-71.7	4.3
3NU6	-0.69	-47.8	4.1	-64.5	5.4	-71.7	5.6	-72.5	5.5	-72.6	5.5	-72.4	5.5	-72.3	5.6	-72.2	5.5
3NU9	-0.11	-46.0	4.7	-64.9	5.6	-70.3	5.4	-70.7	5.5	-70.6	5.5	-70.5	5.5	-70.4	5.7	-70.4	5.7
3NUJ	-0.89	-50.6	3.2	-65.8	5.0	-76.4	4.9	-78.7	5.1	-78.9	5.1	-78.8	5.1	-78.6	5.1	-78.5	5.0
3NUO	-1.83	-55.0	3.9	-77.3	5.4	-84.8	5.4	-85.4	5.3	-85.2	5.4	-85.3	5.3	-85.4	5.4	-85.5	5.4

Table S24. Predicted binding energies (kcal/mol) obtained from the analysis of the 4th ns of MD simulations run on GPU for the HIV1-protease system (RUN 3).

		Nwat = 0		Nwat = 10		Nwat = 20		Nwat = 30		Nwat = 40		Nwat = 50		Nwat = 60		Nwat = 70	
**Cmp**	**$ln(k_i)$**	**ΔG_{bind}**	**σ**	**ΔG_{bind}**	**σ**	**ΔG_{bind}**	**σ**	**ΔG_{bind}**	**σ**	**ΔG_{bind}**	**σ**	**ΔG_{bind}**	**σ**	**ΔG_{bind}**	**σ**	**ΔG_{bind}**	**σ**
3NDW	-2.81	-48.2	3.9	-68.0	5.4	-75.6	5.5	-82.3	5.6	-85.7	5.6	-87.1	5.6	-87.7	5.7	-87.9	-87.7
3NDX	-2.81	-49.5	4.2	-62.5	5.4	-68.4	6.2	-76.1	5.8	-82.0	5.6	-84.8	5.3	-86.2	5.3	-87.0	-86.2
3NU3	-1.90	-37.7	5.1	-56.4	5.4	-67.8	5.6	-70.9	5.8	-71.2	5.7	-71.0	5.5	-70.8	5.6	-70.7	-70.8
3NU4	0.41	-43.1	5.1	-64.2	7.4	-75.5	7.2	-79.4	6.1	-80.0	5.9	-80.0	5.9	-79.9	5.9	-79.6	-79.9
3NU5	1.50	-50.5	4.7	-61.9	6.1	-67.3	5.7	-67.9	5.6	-67.8	5.7	-67.7	5.8	-67.7	5.9	-67.8	-67.7
3NU6	-0.69	-54.8	4.7	-68.1	5.8	-72.9	5.8	-73.4	5.7	-73.2	5.6	-73.1	5.6	-73.2	5.6	-73.1	-73.2
3NU9	-0.11	-44.5	5.3	-62.0	6.2	-72.1	5.3	-75.0	5.3	-74.8	5.2	-74.4	5.1	-74.1	5.1	-73.8	-74.1
3NUJ	-0.89	-53.8	3.9	-66.2	5.6	-75.9	5.4	-79.2	5.8	-79.6	5.7	-79.5	5.9	-79.2	5.8	-79.0	-79.2
3NUO	-1.83	-51.2	4.4	-70.1	6.5	-76.8	6.0	-77.9	6.1	-77.8	6.0	-77.7	6.1	-77.7	6.1	-77.7	-77.7

Table S25. Predicted binding energies (kcal/mol) obtained from the analysis of the 4th ns of MD simulations run on HPC for the HIV1-protease system (RUN 1).

		Nwat = 0		Nwat = 10		Nwat = 20		Nwat = 30		Nwat = 40		Nwat = 50		Nwat = 60		Nwat = 70	
**Cmp**	**$ln(k_i)$**	**ΔG_{bind}**	**σ**	**ΔG_{bind}**	**σ**	**ΔG_{bind}**	**σ**	**ΔG_{bind}**	**σ**	**ΔG_{bind}**	**σ**	**ΔG_{bind}**	**σ**	**ΔG_{bind}**	**σ**	**ΔG_{bind}**	**σ**
3NDW	-2.81	-52.9	3.2	-67.1	5.7	-76.0	5.3	-82.5	5.1	-85.7	5.1	-87.4	5.0	-88.4	5.1	-88.7	5.0
3NDX	-2.81	-43.0	3.2	-61.2	7.2	-68.9	7.5	-78.0	7.2	-84.3	6.6	-86.6	6.2	-87.6	6.1	-88.1	6.1
3NU3	-1.90	-48.3	3.6	-70.3	5.9	-78.6	5.9	-80.9	6.0	-81.1	6.0	-81.0	5.9	-80.9	6.0	-80.8	6.0
3NU4	0.41	-49.9	3.9	-65.7	5.6	-71.4	5.6	-72.1	5.5	-71.9	5.7	-71.7	5.6	-71.7	5.6	-71.5	5.7
3NU5	1.50	-31.3	4.2	-50.5	7.1	-63.0	7.1	-71.3	6.9	-74.2	6.6	-74.2	6.4	-73.4	6.5	-72.7	6.4
3NU6	-0.69	-44.8	4.5	-64.9	6.1	-73.6	5.5	-75.1	5.5	-75.2	5.4	-75.0	5.5	-74.8	5.6	-74.6	5.6
3NU9	-0.11	-37.4	2.8	-53.6	5.5	-64.0	5.8	-71.7	5.8	-74.2	5.7	-74.8	5.5	-74.8	5.5	-74.5	5.3
3NUJ	-0.89	-53.6	3.7	-68.6	5.2	-74.8	5.2	-76.2	5.3	-76.3	5.3	-76.2	5.4	-76.0	5.4	-75.9	5.4
3NUO	-1.83	-56.6	4.3	-71.2	5.7	-76.2	5.4	-76.8	5.3	-76.9	5.3	-77.1	5.3	-77.1	5.4	-77.1	5.5

Table S26. Predicted binding energies (kcal/mol) obtained from the analysis of the 4th ns of MD simulations run on HPC for the HIV1-protease system (RUN 2).

		Nwat = 0		Nwat = 10		Nwat = 20		Nwat = 30		Nwat = 40		Nwat = 50		Nwat = 60		Nwat = 70	
**Cmp**	**ln(k_i)**	**ΔG_{bind}**	**σ**	**ΔG_{bind}**	**σ**	**ΔG_{bind}**	**σ**	**ΔG_{bind}**	**σ**	**ΔG_{bind}**	**σ**	**ΔG_{bind}**	**σ**	**ΔG_{bind}**	**σ**	**ΔG_{bind}**	**σ**
3NDW	-2.81	-48.7	3.6	-68.7	6.0	-76.0	6.0	-81.9	6.0	-84.8	6.1	-86.1	6.2	-86.6	6.1	-86.7	6.1
3NDX	-2.81	-40.5	3.9	-61.0	5.9	-67.5	6.7	-75.3	6.7	-82.1	6.1	-86.0	5.5	-87.7	5.6	-88.6	5.6
3NU3	-1.90	-47.3	4.1	-70.3	5.4	-76.4	5.4	-77.4	5.5	-77.8	5.4	-77.9	5.5	-77.8	5.5	-77.6	5.5
3NU4	0.41	-42.7	5.6	-60.0	6.8	-67.0	5.4	-67.8	5.4	-67.6	5.5	-67.5	5.6	-67.5	5.6	-67.4	5.7
3NU5	1.50	-45.1	4.5	-61.4	6.1	-72.7	5.8	-75.4	5.6	-75.8	5.6	-75.8	5.7	-75.8	5.7	-75.7	5.7
3NU6	-0.69	-46.4	4.8	-63.8	6.1	-69.7	6.1	-70.4	6.2	-70.3	6.3	-70.2	6.3	-70.0	6.3	-69.9	6.3
3NU9	-0.11	-48.4	3.8	-67.4	5.7	-72.7	5.5	-73.0	5.5	-72.7	5.5	-72.8	5.5	-72.9	5.5	-72.8	5.5
3NUJ	-0.89	-58.4	4.2	-69.7	5.8	-77.6	5.8	-79.9	5.9	-80.3	5.9	-80.3	5.8	-80.0	5.8	-79.8	5.9
3NUO	-1.83	-56.5	4.0	-69.4	5.1	-77.7	4.9	-79.3	4.8	-79.5	5.0	-79.4	4.9	-79.3	5.0	-79.2	5.0

Table S27. Predicted binding energies (kcal/mol) obtained from the analysis of the 4th ns of MD simulations run on HPC for the HIV1-protease system (RUN 3).

		Nwat = 0		Nwat = 10		Nwat = 20		Nwat = 30		Nwat = 40		Nwat = 50		Nwat = 60		Nwat = 70	
**Cmp**	**ln(k_i)**	**ΔG_{bind}**	**σ**	**ΔG_{bind}**	**σ**	**ΔG_{bind}**	**σ**	**ΔG_{bind}**	**σ**	**ΔG_{bind}**	**σ**	**ΔG_{bind}**	**σ**	**ΔG_{bind}**	**σ**	**ΔG_{bind}**	**σ**
3NDW	-2.81	-50.2	3.6	-67.6	4.9	-74.1	5.3	-80.6	5.2	-83.3	5.3	-84.9	5.4	-85.5	5.4	-85.8	5.3
3NDX	-2.81	-44.4	2.6	-56.2	5.9	-62.1	5.8	-71.4	6.3	-78.8	6.2	-82.9	6.2	-84.8	6.1	-85.4	6.2
3NU3	-1.90	-42.5	2.9	-62.5	4.6	-71.2	4.4	-72.7	4.4	-74.0	4.4	-74.4	4.4	-74.4	4.4	-74.3	4.5
3NU4	0.41	-38.2	3.1	-57.1	6.0	-70.6	6.3	-77.6	5.8	-79.3	5.9	-79.5	5.8	-79.4	5.8	-79.1	5.7
3NU5	1.50	-53.6	4.6	-64.2	5.3	-69.7	5.6	-70.2	5.6	-70.4	5.7	-70.3	5.6	-70.2	5.5	-70.0	5.5
3NU6	-0.69	-37.9	3.6	-54.6	5.3	-65.4	5.2	-71.4	5.0	-72.6	5.4	-72.4	5.4	-72.1	5.3	-71.7	5.2
3NU9	-0.11	-39.4	3.3	-48.4	5.5	-61.5	6.1	-67.3	6.0	-68.0	5.9	-67.8	5.9	-67.7	5.9	-67.9	5.8
3NUJ	-0.89	-51.6	3.6	-68.0	5.2	-75.7	5.1	-77.4	5.2	-77.6	5.1	-77.7	5.2	-77.5	5.2	-77.4	5.3
3NUO	-1.83	-56.4	5.2	-71.3	5.8	-75.5	5.6	-75.8	5.5	-75.9	5.6	-76.1	5.6	-76.2	5.6	-76.2	5.6

Table S28. Predicted binding energies (kcal/mol) obtained from the analysis of the 1st ns of MD simulations run on GPU for the BCL-X_L system (RUN 1).

		Nwat = 0		Nwat = 10		Nwat = 20		Nwat = 30		Nwat = 40		Nwat = 50	
**Cmp**	**log(IC_{50})**	**ΔG_{bind}**	**σ**	**ΔG_{bind}**	**σ**	**ΔG_{bind}**	**σ**	**ΔG_{bind}**	**σ**	**ΔG_{bind}**	**σ**	**ΔG_{bind}**	**σ**
3Z1B	0.3	-48.0	4.1	-68.7	5.6	-73.9	5.7	-77.3	5.8	-79.3	5.9	-80.6	6.0
3ZC4	-1.6	-48.9	4.4	-68.6	6.7	-73.7	6.6	-77.4	6.4	-79.7	6.5	-81.3	6.7
3ZC5	-2.2	-67.2	3.5	-86.1	5.6	-91.0	5.8	-95.8	6.0	-98.7	6.3	-100.7	6.3
3ZK6	-0.1	-46.5	3.2	-51.5	4.3	-51.8	4.7	-54.2	4.7	-57.3	4.8	-60.1	4.8
3ZLN	-1.6	-52.1	2.5	-72.5	5.0	-78.6	5.1	-82.2	5.4	-84.6	5.5	-86.5	5.6
3ZLO	-2.8	-68.2	4.2	-87.3	6.2	-91.8	6.3	-95.8	6.5	-98.2	6.5	-100.0	6.8
3ZLR	-3.0	-68.2	4.2	-87.3	6.2	-91.8	6.3	-95.8	6.5	-98.2	6.6	-100.0	6.8

Table S29. Predicted binding energies (kcal/mol) obtained from the analysis of the 1st ns of MD simulations run on GPU for the BCL-X_L system (RUN 2).

		Nwat = 0		Nwat = 10		Nwat = 20		Nwat = 30		Nwat = 40		Nwat = 50	
**Cmp**	**log(IC_{50})**	**ΔG_{bind}**	**σ**	**ΔG_{bind}**	**σ**	**ΔG_{bind}**	**σ**	**ΔG_{bind}**	**σ**	**ΔG_{bind}**	**σ**	**ΔG_{bind}**	**σ**
3Z1B	0.3	-42.9	2.9	-57.8	5.0	-66.0	5.6	-72.0	6.3	-74.9	6.7	-76.3	6.9
3ZC4	-1.6	-44.9	3.4	-59.5	4.9	-67.9	5.5	-74.2	6.6	-77.2	7.2	-78.5	7.4
3ZC5	-2.2	-59.1	3.2	-71.8	5.1	-80.9	5.6	-90.2	6.3	-97.5	7.0	-102.0	7.3
3ZK6	-0.1	-39.6	3.6	-39.9	6.0	-45.3	6.5	-55.4	6.7	-65.7	6.3	-73.5	6.3
3ZLN	-1.6	-43.7	2.9	-58.3	5.1	-66.9	5.4	-74.6	6.4	-78.7	6.9	-80.8	7.2
3ZLO	-2.8	-60.0	3.3	-73.3	5.3	-82.6	5.7	-91.5	6.0	-97.1	6.0	-100.2	6.7
3ZLR	-3.0	-59.2	3.7	-86.9	5.4	-98.7	6.3	-109.6	7.2	-117.7	7.8	-122.2	8.1

Table S30. Predicted binding energies (kcal/mol) obtained from the analysis of the 1st ns of MD simulations run on GPU for the BCL-X_L system (RUN 3).

		Nwat = 0		Nwat = 10		Nwat = 20		Nwat = 30		Nwat = 40		Nwat = 50	
**Cmp**	**log(IC_{50})**	**ΔG_{bind}**	**σ**	**ΔG_{bind}**	**σ**	**ΔG_{bind}**	**σ**	**ΔG_{bind}**	**σ**	**ΔG_{bind}**	**σ**	**ΔG_{bind}**	**σ**
3Z1B	0.3	-49.0	2.9	-68.1	5.3	-73.0	5.7	-76.3	6.1	-78.4	6.3	-80.1	6.4
3ZC4	-1.6	-54.2	4.0	-73.5	5.5	-78.9	5.4	-81.8	5.5	-84.0	5.7	-85.6	5.8
3ZC5	-2.2	-69.5	4.0	-88.6	6.1	-93.9	6.3	-97.9	6.4	-100.5	6.3	-102.5	6.4
3ZK6	-0.1	-49.5	3.4	-54.0	5.1	-54.5	5.2	-57.2	5.4	-60.3	5.7	-62.4	5.8
3ZLN	-1.6	-55.6	3.0	-72.9	4.9	-78.0	5.0	-81.2	5.4	-83.3	5.5	-85.1	5.7
3ZLO	-2.8	-65.3	4.1	-84.1	5.7	-88.5	6.0	-92.6	6.0	-95.2	6.0	-97.0	6.2
3ZLR	-3.0	-75.4	3.8	-104.5	6.5	-111.7	6.7	-117.3	6.7	-120.6	6.8	-122.7	7.0

Table S31. Predicted binding energies (kcal/mol) obtained from the analysis of the 1st ns of MD simulations run on HPC for the BCL-X_L system (RUN 1).

		Nwat = 0		Nwat = 10		Nwat = 20		Nwat = 30		Nwat = 40		Nwat = 50	
**Cmp**	**log(IC_{50})**	**ΔG_{bind}**	**σ**	**ΔG_{bind}**	**σ**	**ΔG_{bind}**	**σ**	**ΔG_{bind}**	**σ**	**ΔG_{bind}**	**σ**	**ΔG_{bind}**	**σ**
3Z1B	0.3	-42.5	2.8	-56.2	4.9	-64.6	5.2	-71.6	6.1	-75.2	6.4	-77.1	6.5
3ZC4	-1.6	-44.7	3.0	-58.6	4.2	-67.6	5.2	-75.0	6.4	-78.1	6.6	-79.6	6.9
3ZC5	-2.2	-61.3	3.3	-74.2	5.6	-84.0	5.9	-92.8	6.6	-98.9	7.2	-102.2	7.3
3ZK6	-0.1	-43.7	4.0	-45.6	6.0	-51.0	7.0	-61.2	7.6	-71.1	7.3	-78.3	6.4
3ZLN	-1.6	-44.4	3.0	-59.5	5.5	-68.8	5.7	-75.8	6.1	-79.8	6.4	-81.7	6.7
3ZLO	-2.8	-61.4	3.6	-75.0	6.2	-84.0	6.7	-92.2	7.3	-97.5	7.3	-100.5	7.3
3ZLR	-3.0	-60.7	4.3	-88.6	6.3	-100.4	6.5	-111.6	6.8	-120.0	7.4	-124.5	7.8

Table S32. Predicted binding energies (kcal/mol) obtained from the analysis of the 1st ns of MD simulations run on HPC for the BCL-X_L system (RUN 2).

		Nwat = 0		Nwat = 10		Nwat = 20		Nwat = 30		Nwat = 40		Nwat = 50	
**Cmp**	**log(IC_{50})**	**ΔG_{bind}**	**σ**	**ΔG_{bind}**	**σ**	**ΔG_{bind}**	**σ**	**ΔG_{bind}**	**σ**	**ΔG_{bind}**	**σ**	**ΔG_{bind}**	**σ**
3Z1B	0.3	-42.5	2.8	-56.8	4.5	-65.5	5.4	-71.8	5.9	-75.7	6.5	-77.3	6.8
3ZC4	-1.6	-45.5	3.4	-60.1	4.8	-68.6	5.8	-74.8	6.9	-77.7	7.3	-79.2	7.6
3ZC5	-2.2	-62.0	3.2	-75.8	5.2	-85.9	5.4	-94.5	6.4	-100.1	6.4	-103.0	6.8
3ZK6	-0.1	-37.1	3.6	-37.9	6.1	-41.9	7.3	-52.0	6.9	-62.2	6.6	-70.9	6.4
3ZLN	-1.6	-44.3	3.2	-59.5	5.2	-68.1	5.4	-75.6	5.9	-79.7	6.3	-81.8	6.5
3ZLO	-2.8	-60.8	3.6	-74.7	5.0	-84.2	5.6	-92.5	6.1	-98.4	6.1	-101.1	6.7
3ZLR	-3.0	-60.8	3.3	-89.1	6.0	-102.3	6.3	-114.0	7.0	-123.1	7.4	-127.4	7.6

Table S33. Predicted binding energies (kcal/mol) obtained from the analysis of the 1st ns of MD simulations run on HPC for the BCL-X_L system (RUN 3).

		Nwat = 0		Nwat = 10		Nwat = 20		Nwat = 30		Nwat = 40		Nwat = 50	
**Cmp**	**log(IC_{50})**	**ΔG_{bind}**	**σ**	**ΔG_{bind}**	**σ**	**ΔG_{bind}**	**σ**	**ΔG_{bind}**	**σ**	**ΔG_{bind}**	**σ**	**ΔG_{bind}**	**σ**
3Z1B	0.3	-43.8	2.6	-58.4	5.0	-66.6	5.7	-73.3	6.6	-76.3	6.9	-77.9	7.2
3ZC4	-1.6	-44.5	3.7	-56.4	7.2	-61.6	11.2	-66.3	13.3	-68.7	14.0	-69.8	14.2
3ZC5	-2.2	-61.6	3.7	-74.2	5.8	-83.5	5.8	-92.2	6.7	-97.7	7.2	-100.9	7.4
3ZK6	-0.1	-39.1	3.4	-38.4	6.6	-42.5	7.1	-52.6	7.4	-63.2	7.0	-71.7	7.0
3ZLN	-1.6	-46.6	2.6	-60.5	4.3	-68.8	5.2	-75.5	5.9	-79.4	6.4	-81.3	6.6
3ZLO	-2.8	-60.3	3.3	-72.6	4.9	-82.3	5.3	-90.5	5.8	-96.3	5.8	-99.4	6.6
3ZLR	-3.0	-66.3	4.0	-91.6	6.6	-103.5	7.1	-113.5	7.7	-120.2	8.1	-123.4	8.3

Table S34. Predicted binding energies (kcal/mol) obtained from the analysis of the 4th ns of MD simulations run on GPU for the BCL-X_L system (RUN 1).

		Nwat = 0		Nwat = 10		Nwat = 20		Nwat = 30		Nwat = 40		Nwat = 50	
**Cmp**	**log(IC$_{50}$)**	**ΔG_{bind}**	**σ**	**ΔG_{bind}**	**σ**	**ΔG_{bind}**	**σ**	**ΔG_{bind}**	**σ**	**ΔG_{bind}**	**σ**	**ΔG_{bind}**	**σ**
3Z1B	0.3	-41.3	3.0	-57.3	4.3	-66.4	5.4	-74.1	6.5	-78.0	7.1	-79.7	7.3
3ZC4	-1.6	-39.8	2.5	-55.4	4.7	-64.3	5.5	-72.4	6.5	-77.8	7.2	-80.1	7.5
3ZC5	-2.2	-59.4	3.8	-72.6	5.4	-81.6	5.8	-91.3	6.7	-99.2	7.4	-103.6	7.5
3ZK6	-0.1	-39.6	3.2	-39.5	5.7	-43.3	6.0	-53.1	6.2	-64.3	6.3	-74.0	6.0
3ZLN	-1.6	-39.7	2.5	-55.1	4.8	-65.4	5.1	-73.9	5.6	-79.3	6.0	-82.0	6.2
3ZLO	-2.8	-60.8	2.9	-74.1	5.5	-83.7	5.7	-91.7	6.4	-96.9	6.4	-99.4	6.8
3ZLR	-3.0	-64.9	4.3	-90.0	6.2	-100.8	5.9	-111.7	6.4	-119.4	6.8	-123.2	7.0

Table S35. Predicted binding energies (kcal/mol) obtained from the analysis of the 4th ns of MD simulations run on GPU for the BCL-X_L system (RUN 2).

		Nwat = 0		Nwat = 10		Nwat = 20		Nwat = 30		Nwat = 40		Nwat = 50	
**Cmp**	**log(IC$_{50}$)**	**ΔG_{bind}**	**σ**	**ΔG_{bind}**	**σ**	**ΔG_{bind}**	**σ**	**ΔG_{bind}**	**σ**	**ΔG_{bind}**	**σ**	**ΔG_{bind}**	**σ**
3Z1B	0.3	-41.9	2.9	-55.1	5.0	-63.9	5.3	-70.7	6.1	-74.2	6.5	-75.9	6.8
3ZC4	-1.6	-44.2	3.3	-58.7	4.3	-67.4	5.1	-74.0	6.0	-77.5	6.4	-79.2	6.6
3ZC5	-2.2	-58.1	3.9	-72.7	5.8	-82.2	6.2	-91.8	6.5	-98.8	6.9	-102.4	7.0
3ZK6	-0.1	-41.8	3.5	-43.5	6.0	-49.8	7.0	-60.0	6.9	-69.3	6.6	-76.1	6.3
3ZLN	-1.6	-44.3	2.6	-60.2	4.8	-69.1	5.2	-76.2	6.1	-80.4	6.5	-82.5	6.7
3ZLO	-2.8	-60.8	3.4	-73.9	5.6	-82.7	6.2	-90.5	6.8	-95.5	6.8	-98.4	7.4
3ZLR	-3.0	-64.4	3.8	-89.5	6.8	-100.6	6.4	-110.8	7.1	-118.0	7.3	-121.8	7.6

Table S36. Predicted binding energies (kcal/mol) obtained from the analysis of the 4th ns of MD simulations run on GPU for the BCL-X_L system (RUN 3).

		Nwat = 0		Nwat = 10		Nwat = 20		Nwat = 30		Nwat = 40		Nwat = 50	
**Cmp**	**log(IC$_{50}$)**	**ΔG_{bind}**	**σ**	**ΔG_{bind}**	**σ**	**ΔG_{bind}**	**σ**	**ΔG_{bind}**	**σ**	**ΔG_{bind}**	**σ**	**ΔG_{bind}**	**σ**
3Z1B	0.3	-48.5	3.0	-66.6	5.7	-71.2	6.1	-74.2	6.4	-76.4	6.5	-77.9	6.6
3ZC4	-1.6	-53.4	3.7	-73.3	5.3	-78.5	5.4	-81.6	5.4	-83.9	5.6	-85.5	5.8
3ZC5	-2.2	-71.2	3.4	-91.4	5.7	-97.6	5.7	-101.8	5.9	-104.3	6.1	-106.2	6.0
3ZK6	-0.1	-50.8	3.4	-56.1	4.6	-57.3	4.8	-60.3	5.0	-63.1	5.0	-65.1	5.0
3ZLN	-1.6	-55.1	3.3	-73.7	5.9	-78.5	5.9	-81.6	6.1	-83.9	6.4	-85.8	6.4
3ZLO	-2.8	-65.8	4.2	-84.3	6.8	-88.3	6.4	-92.3	6.4	-94.8	6.4	-96.7	6.5
3ZLR	-3.0	-75.5	3.4	-105.9	5.9	-112.7	6.1	-118.3	6.3	-121.3	6.5	-123.4	6.8

Table S37. Predicted binding energies (kcal/mol) obtained from the analysis of the 4th ns of MD simulations run on HPC for the BCL-X_L system (RUN 1).

		**Nwat = 0**		**Nwat = 10**		**Nwat = 20**		**Nwat = 30**		**Nwat = 40**		**Nwat = 50**	
**Cmp**	**log(IC_{50})**	**ΔG_{bind}**	**σ**	**ΔG_{bind}**	**σ**	**ΔG_{bind}**	**σ**	**ΔG_{bind}**	**σ**	**ΔG_{bind}**	**σ**	**ΔG_{bind}**	**σ**
3Z1B	0.3	-42.1	3.2	-54.4	4.9	-62.9	5.4	-69.3	5.9	-73.0	6.5	-74.8	6.9
3ZC4	-1.6	-40.1	2.6	-55.7	5.1	-64.8	5.5	-73.3	6.4	-78.5	7.0	-80.6	7.4
3ZC5	-2.2	-60.7	3.0	-74.1	4.9	-83.7	5.7	-92.6	6.2	-98.6	6.3	-102.2	6.6
3ZK6	-0.1	-42.5	3.9	-44.0	6.3	-49.2	6.8	-58.7	7.0	-69.0	6.7	-76.7	6.6
3ZLN	-1.6	-45.3	3.0	-59.6	4.6	-67.9	5.1	-74.9	6.0	-79.1	6.4	-81.1	6.8
3ZLO	-2.8	-61.6	3.5	-74.8	5.5	-83.5	5.9	-91.8	6.7	-97.0	7.2	-100.0	7.2
3ZLR	-3.0	-59.8	3.2	-88.1	5.7	-100.5	5.6	-112.0	6.3	-119.8	6.4	-124.1	6.9

Table S38. Predicted binding energies (kcal/mol) obtained from the analysis of the 4th ns of MD simulations run on HPC for the BCL-X_L system (RUN 2).

		**Nwat = 0**		**Nwat = 10**		**Nwat = 20**		**Nwat = 30**		**Nwat = 40**		**Nwat = 50**	
**Cmp**	**log(IC_{50})**	**ΔG_{bind}**	**σ**	**ΔG_{bind}**	**σ**	**ΔG_{bind}**	**σ**	**ΔG_{bind}**	**σ**	**ΔG_{bind}**	**σ**	**ΔG_{bind}**	**σ**
3Z1B	0.3	-42.7	3.2	-57.7	5.5	-65.8	5.5	-72.1	6.2	-75.3	6.3	-76.8	6.5
3ZC4	-1.6	-45.6	3.5	-59.7	4.4	-68.2	4.9	-74.6	5.8	-77.8	6.1	-79.3	6.2
3ZC5	-2.2	-60.9	3.4	-73.4	5.0	-83.3	5.4	-92.9	6.0	-99.5	6.4	-103.3	6.7
3ZK6	-0.1	-39.4	3.3	-40.3	5.2	-44.7	6.1	-54.3	6.3	-64.4	5.8	-72.0	5.7
3ZLN	-1.6	-46.5	2.5	-59.1	4.8	-67.3	5.0	-74.2	6.1	-78.1	6.7	-80.0	7.1
3ZLO	-2.8	-57.7	3.5	-70.0	5.8	-78.3	6.2	-86.8	6.5	-93.2	6.5	-97.2	7.3
3ZLR	-3.0	-66.4	4.0	-90.6	7.0	-101.9	7.2	-112.2	8.0	-119.3	8.5	-122.8	8.8

Table S39. Predicted binding energies (kcal/mol) obtained from the analysis of the 4th ns of MD simulations run on HPC for the BCL-X_L system (RUN 3).

		**Nwat = 0**		**Nwat = 10**		**Nwat = 20**		**Nwat = 30**		**Nwat = 40**		**Nwat = 50**	
**Cmp**	**log(IC_{50})**	**ΔG_{bind}**	**σ**	**ΔG_{bind}**	**σ**	**ΔG_{bind}**	**σ**	**ΔG_{bind}**	**σ**	**ΔG_{bind}**	**σ**	**ΔG_{bind}**	**σ**
3Z1B	0.3	-41.6	2.7	-58.2	4.9	-68.8	5.5	-76.1	6.5	-80.1	6.7	-82.1	6.8
3ZC4	-1.6	-42.1	3.2	-59.0	4.4	-68.3	5.0	-75.9	6.1	-79.5	6.1	-81.3	6.2
3ZC5	-2.2	-62.4	3.5	-75.1	5.3	-84.9	5.6	-93.3	6.2	-98.9	6.8	-102.0	7.1
3ZK6	-0.1	-40.3	3.4	-41.1	5.9	-46.1	6.9	-56.4	7.6	-66.0	7.0	-73.7	6.6
3ZLN	-1.6	-45.2	2.7	-60.8	5.1	-70.3	5.6	-77.0	6.2	-80.4	6.7	-82.0	6.9
3ZLO	-2.8	-60.4	3.4	-73.9	4.5	-82.9	5.1	-91.2	5.7	-96.9	5.7	-99.7	5.9
3ZLR	-3.0	-60.0	3.6	-86.7	5.6	-97.3	5.9	-108.9	7.1	-117.5	8.0	-122.6	8.3

Table S40. Correlation values between experimental data and predicted binding energies obtained for the penicillopepsin system.

GPU, 1 ns	RUN 1	RUN 2	RUN 3			HPC, 1 ns	RUN 1	RUN 2	RUN 3		
NWAT	r^2	r^2	r^2	Avg	St. Dev		r^2	r^2	r^2	Avg	St. Dev
0	0.38	0.31	0.17	0.29	0.11		0.18	0.50	0.15	0.28	0.19
10	0.8	0.71	0.77	0.76	0.05		0.57	0.93	0.67	0.72	0.19
20	0.8	0.71	0.81	0.77	0.06		0.61	0.91	0.72	0.75	0.15
30	0.79	0.72	0.83	0.78	0.06		0.69	0.91	0.76	0.79	0.11
40	0.78	0.73	0.82	0.78	0.05		0.76	0.91	0.76	0.81	0.09
50	0.76	0.74	0.79	0.76	0.03		0.82	0.87	0.75	0.81	0.06
GPU, 4 ns						HPC, 4 ns					
0	0.13	0.17	0.06	0.12	0.06		0.23	0.00	0.00	0.08	0.13
10	0.83	0.82	0.70	0.78	0.07		0.59	0.85	0.89	0.78	0.16
20	0.73	0.78	0.80	0.77	0.04		0.59	0.86	0.89	0.78	0.17
30	0.68	0.73	0.77	0.73	0.05		0.61	0.84	0.86	0.77	0.14
40	0.70	0.67	0.75	0.71	0.04		0.64	0.84	0.81	0.76	0.11
50	0.73	0.61	0.72	0.69	0.07		0.68	0.84	0.74	0.75	0.08

Table S41. Correlation values between experimental data and predicted binding energies obtained for the HIV1-protease system.

GPU, 1 ns	RUN 1	RUN 2	RUN 3			HPC, 1 ns	RUN 1	RUN 2	RUN 3		
NWAT	r^2	r^2	r^2	Avg	St. Dev		r^2	r^2	r^2	Avg	St. Dev
0	0.22	0.06	0.03	0.10	0.10		0.17	0.07	0.03	0.09	0.07
10	0.08	0.03	0.12	0.08	0.05		0.04	0.01	0.12	0.06	0.06
20	0.13	0.09	0.12	0.11	0.02		0.15	0.05	0.13	0.11	0.05
30	0.52	0.60	0.68	0.60	0.08		0.51	0.36	0.61	0.49	0.13
40	0.60	0.77	0.74	0.70	0.09		0.58	0.64	0.71	0.64	0.07
50	0.62	0.80	0.74	0.72	0.09		0.59	0.72	0.72	0.68	0.08
60	0.66	0.80	0.74	0.73	0.07		0.60	0.73	0.72	0.68	0.07
70	0.64	0.80	0.74	0.73	0.08		0.60	0.73	0.72	0.68	0.07
GPU, 4 ns						HPC, 4 ns					
0	0.12	-0.02	0.00	0.03	0.08		0.34	0.01	0.01	0.12	0.19
10	0.13	0.00	0.03	0.05	0.07		0.37	0.19	0.07	0.21	0.15
20	0.19	0.01	0.02	0.07	0.10		0.4	0.06	0.01	0.16	0.21
30	0.53	0.24	0.23	0.33	0.17		0.8	0.32	0.13	0.42	0.35
40	0.65	0.50	0.40	0.52	0.13		0.77	0.52	0.36	0.55	0.21
50	0.67	0.58	0.45	0.57	0.11		0.76	0.57	0.45	0.59	0.16
60	0.66	0.60	0.47	0.58	0.10		0.77	0.58	0.48	0.61	0.15
70	0.64	0.62	0.48	0.58	0.09		0.79	0.58	0.51	0.63	0.15

Table S42. Correlation values between experimental data and predicted binding energies obtained for the BCL-X_L system.

GPU, 1 ns	RUN 1	RUN 2	RUN 3			HPC, 1 ns	RUN 1	RUN 2	RUN 3		
NWAT	r^2	r^2	r^2	Avg	St. Dev		r^2	r^2	r^2	Avg	St. Dev
0	0.75	0.73	0.79	0.76	0.03		0.7	0.75	0.74	0.73	0.03
10	0.71	0.69	0.72	0.71	0.02		0.75	0.72	0.65	0.71	0.05
20	0.67	0.68	0.69	0.68	0.01		0.74	0.69	0.62	0.68	0.06
30	0.67	0.71	0.69	0.69	0.02		0.73	0.71	0.62	0.69	0.06
40	0.68	0.71	0.69	0.69	0.02		0.69	0.7	0.61	0.67	0.05
50	0.69	0.69	0.69	0.69	0.00		0.65	0.68	0.58	0.64	0.05
GPU, 4 ns						HPC, 4 ns					
0	0.63	0.76	0.75	0.71	0.07		0.64	0.7	0.76	0.67	0.04
10	0.65	0.75	0.74	0.71	0.06		0.75	0.7	0.65	0.73	0.04
20	0.65	0.74	0.72	0.70	0.05		0.73	0.66	0.64	0.70	0.05
30	0.65	0.73	0.71	0.70	0.04		0.74	0.66	0.65	0.70	0.06
40	0.66	0.71	0.71	0.69	0.03		0.73	0.66	0.66	0.70	0.05
50	0.65	0.68	0.71	0.68	0.03		0.69	0.63	0.66	0.66	0.04

Table S43. Two-tailed t test (p < 0.05) between the r^2 values for Nwat = 0 ($r^2_{avg,0}$) and for each Nwat ($r^2_{avg,X}$) at any simulation condition and for all the systems (H_0: the two average r^2 are not significantly different).

**Penicillopepsin**							
GPU 1 ns					HPC 1 ns		
Nwat = 0 $r^2_{avg,0}$ = 0.29, Var = 0.0114					Nwat = 0 $r^2_{avg,0}$ = 0.28, Var = 0.0376		
Nwat	$r^2_{avg,X}$	Var_X	P(T<=t) two tails		$r^2_{avg,X}$	Varx	P(T<=t) two tails
10	0.76	0.0021	0.0059		0.72	0.0345	0.0450
20	0.77	0.003	0.0060		0.75	0.023	0.0298
30	0.78	0.0031	0.0058		0.79	0.0126	0.0291
40	0.78	0.002	0.0053		0.81	0.0075	0.0225
50	0.76	0.0006	0.0172		0.81	0.0036	0.0446
GPU 4 ns					HPC 4 ns		
Nwat = 0 $r^2_{avg,0}$ = 0.12, Var = 0.0031					Nwat = 0 $r^2_{avg,0}$ = 0.08, Var = 0.0176		
Nwat	$r^2_{avg,X}$	Varx	P(T<=t) two tails		$r^2_{avg,X}$	Varx	P(T<=t) two tails
10	0.78	0.0052	0.0002		0.78	0.0265	0.0045
20	0.77	0.0013	0.0004		0.78	0.0273	0.0045
30	0.73	0.002	0.0001		0.77	0.0193	0.0033
40	0.71	0.0016	0.0001		0.76	0.0116	0.0022
50	0.69	0.0044	0.0003		0.75	0.0065	0.0048
**HIV1-Protease**							
GPU 1 ns					HPC 1 ns		
Nwat = 0 $r^2_{avg,0}$ = 0.10, Var = 0.0104					Nwat = 0 $r^2_{avg,0}$ = 0.09, Var = 0.0052		
Nwat	$r^2_{avg,X}$	Varx	P(T<=t) two tails		$r^2_{avg,X}$	Varx	P(T<=t) two tails
10	0.08	0.002	0.7069		0.06	0.0032	0.5637
20	0.11	0.0004	0.8833		0.11	0.0028	0.7182
30	0.60	0.0064	0.0027		0.49	0.0158	0.0170
40	0.7	0.0082	0.0016		0.64	0.0042	0.0006
50	0.72	0.0084	0.0015		0.68	0.0056	0.0006
60	0.73	0.0049	0.0009		0.68	0.0052	0.0005
GPU 4 ns					HPC 4 ns		
Nwat = 0 $r^2_{avg,0}$ = 0.03, Var = 0.0057					Nwat = 0 $r^2_{avg,0}$ = 0.12, Var = 0.0363		
Nwat	$r^2_{avg,X}$	Varx	P(T<=t) two tails		$r^2_{avg,X}$	Varx	P(T<=t) two tails
10	0.05	0.0046	0.7508		0.21	0.0228	0.5563
20	0.07	0.0102	0.6126		0.16	0.045	0.8347
30	0.33	0.029	0.0686		0.42	0.1192	0.2836
40	0.52	0.0158	0.0107		0.55	0.0427	0.0570
50	0.57	0.0122	0.0023		0.59	0.0244	0.0292
60	0.58	0.0094	0.0016		0.61	0.0217	0.0244

**BCL-X$_L$**							
GPU 1 ns					HPC 1 ns		
Nwat = 0 $r^2_{avg,0}$ = 0.76, Var = 0.0009					Nwat = 0 $r^2_{avg,0}$ = 0.73, Var = 0.0007		
Nwat	$r^2_{avg,X}$	Varx	P(T<=t) two tails		$r^2_{avg,X}$	Varx	P(T<=t) two tails
10	0.71	0.0002	0.0850		0.71	0.0026	0.5343
20	0.68	0.0001	0.0539		0.68	0.0036	0.3070
30	0.69	0.0004	0.0508		0.69	0.0034	0.3274
40	0.69	0.0002	0.0489		0.67	0.0024	0.1449
50	0.69	0.0000	0.0634		0.64	0.0026	0.0678
GPU 4 ns					HPC 4 ns		
Nwat = 0 $r^2_{avg,0}$ = 0.71, Var = 0.0052					Nwat = 0 $r^2_{avg,0}$ = 0.70, Var = 0.0036		
Nwat	$r^2_{avg,X}$	Varx	P(T<=t) two tails		$r^2_{avg,X}$	Varx	P(T<=t) two tails
10	0.71	0.0030	1.0000		0.70	0.0025	1.0000
20	0.70	0.0022	0.8540		0.68	0.0022	0.6247
30	0.70	0.0017	0.7523		0.68	0.0024	0.7290
40	0.69	0.0008	0.6866		0.68	0.0016	0.7102
50	0.68	0.0009	0.5144		0.66	0.0009	0.3777

Table S44. Water-mediated H-bonds with occupancy > 20% detected from the analysis of a CPU MD simulation of 3NUO. Water-mediated H-bonds involving amprenavir are reported in bold.

**Nwat = 10**		**Nwat = 70**	
**Residues involved**	**occ%**	**Residues involved**	**occ%**
**31:ASP 203:AMP**	**58.0**	**31:ASP 203:AMP**	**58.0**
90:LEU 91:MET	54.0	129:GLY 131:ASP	56.0
129:GLY 131:ASP	51.0	90:LEU 91:MET	54.0
**132:ASP 203:AMP**	**47.0**	80:PRO 152:ILE	54.0
191:LEU 192:MET	41.0	**132:ASP 203:AMP**	**47.0**
		191:LEU 192:MET	41.0
		27:THR 28:GLY	34.0
		52:GLY 181:PRO	30.0
		30:ASP 31:ASP	28.0
		31:ASP 46:LYS	21.0
		128:THR 129:GLY	20.0

Table S45. Water-mediated H-bonds with occupancy > 20% detected from the analysis of a CPU MD simulation of 3NDW. The water-mediated H-bonds involving ritonavir are reported in bold.

**Nwat = 10**		**Nwat = 70**	
**Residues involved**	**occ%**	**Residues involved**	**occ%**
191:LEU 192:LEU	73.0	26:ASP 127:ASP	86.0
26:ASP 127:ASP	70.0	191:LEU 192:LEU	73.0
**132:ASP 203:RIT**	**54.0**	**132:ASP 203:RIT**	**54.0**
90:LEU 91:LEU	53.0	90:LEU 91:LEU	53.0
176:THR 177:VAL	36.0	28:GLY 30:ASP	38.0
		176:THR 177:VAL	36.0

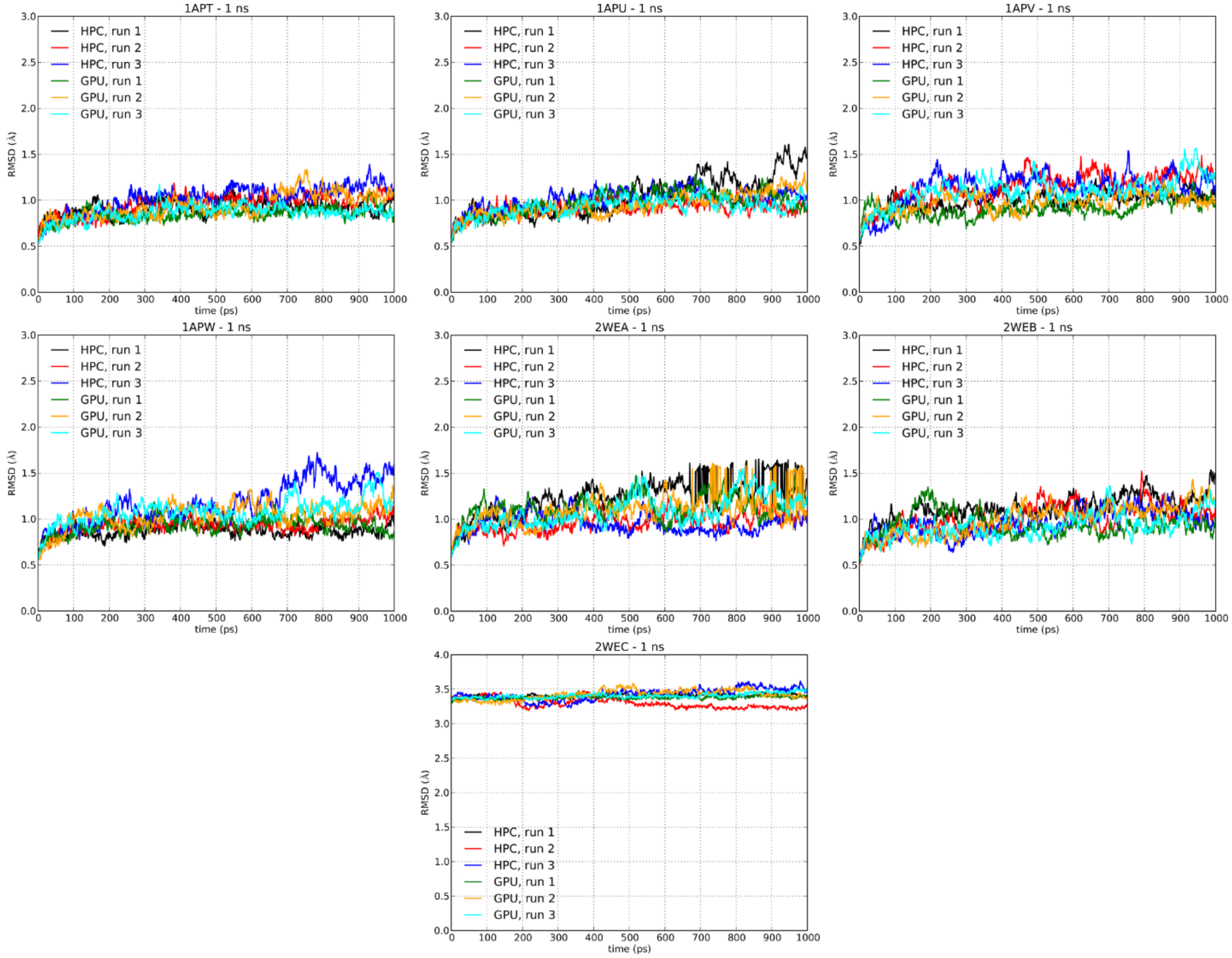

Figure S4. Comparison of RMSD values of the 1st ns of MD simulations of the penicillopepsin system obtained by using GPU or CPU hardware.

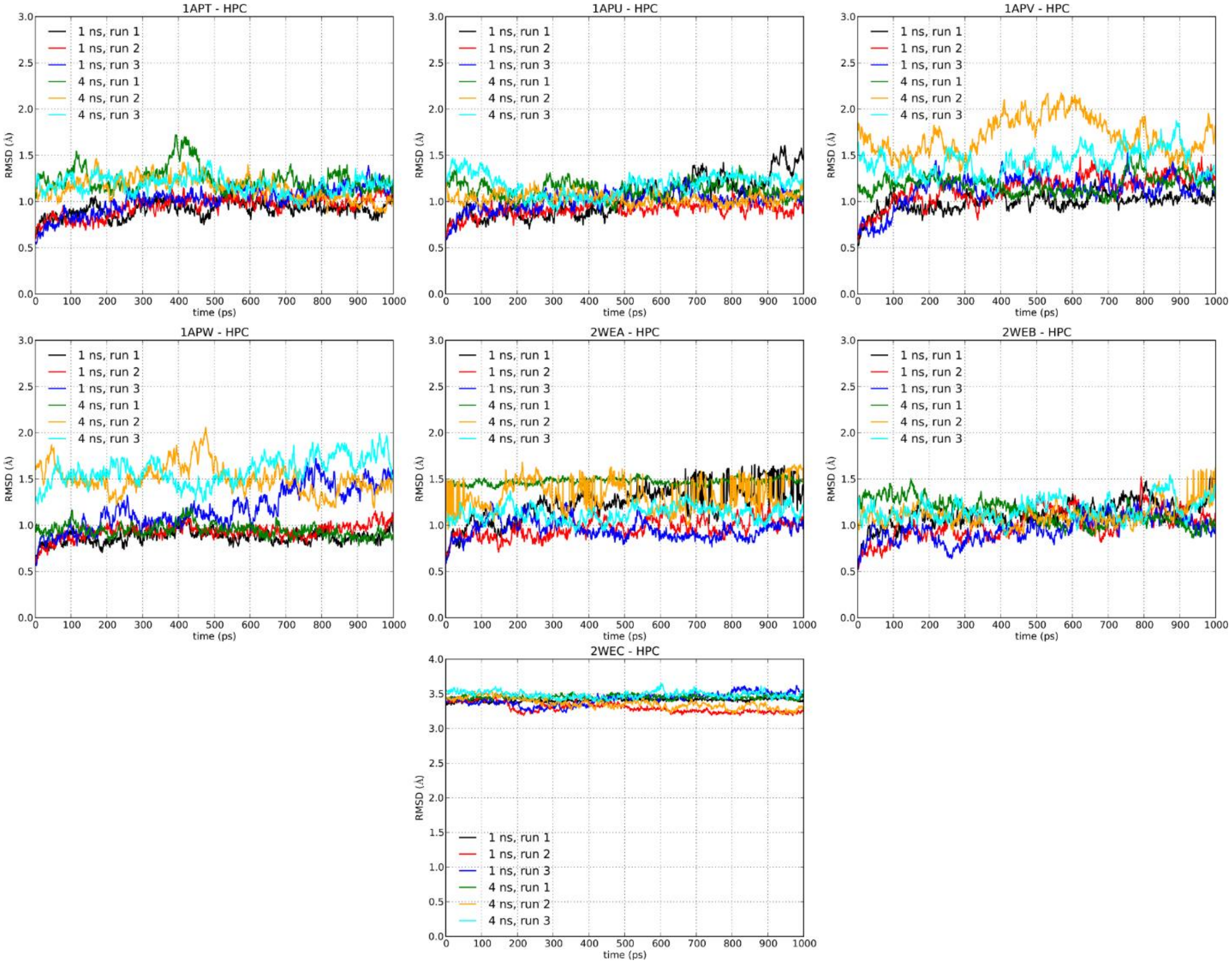

Figure S5. Comparison of RMSD values of the 1st ns and 4th of GPU MD simulations of the penicillopepsin system.

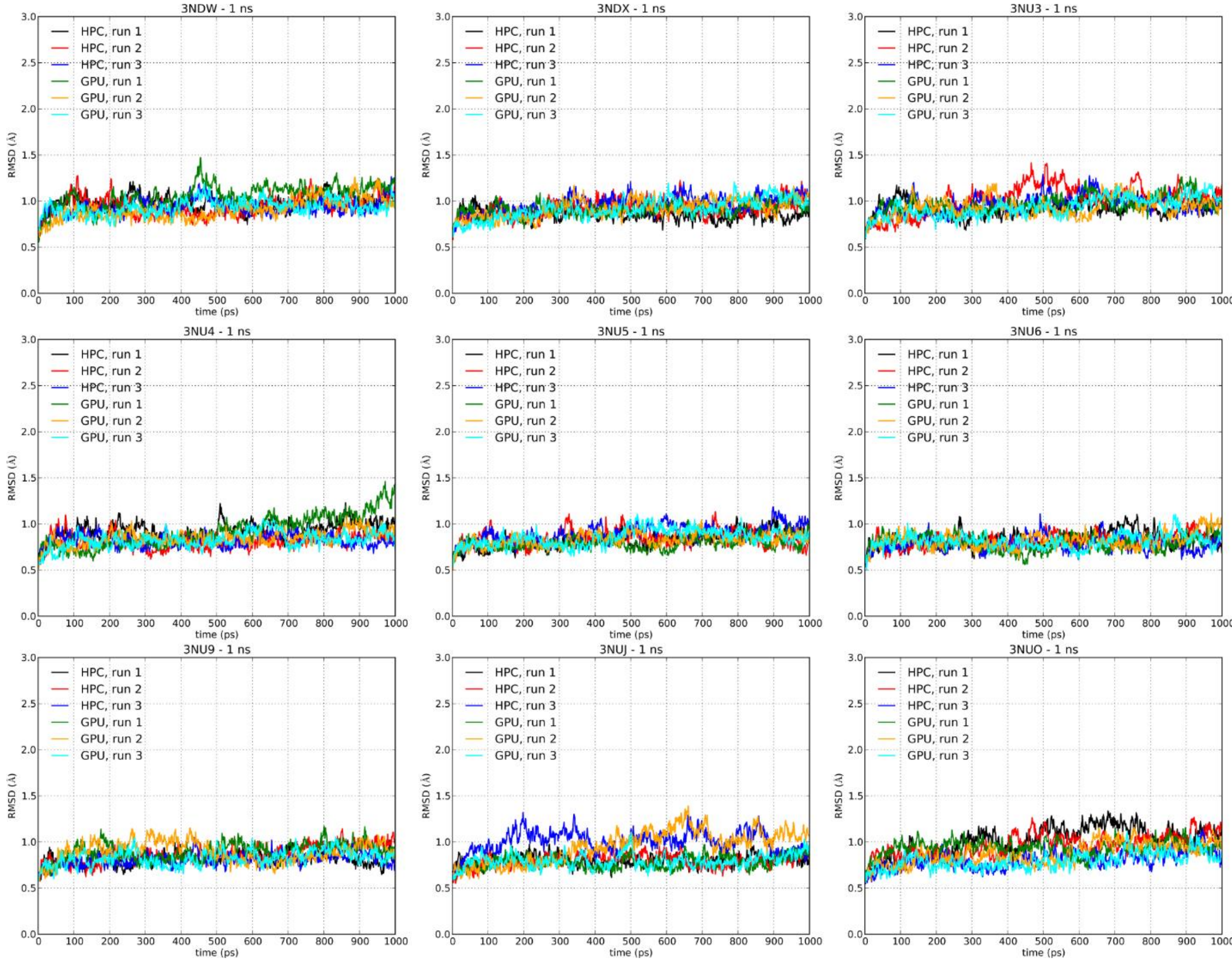

Figure S6. Comparison of RMSD values of the 1st ns of MD simulations of the HIV1-protease system obtained by using GPU or CPU hardware.

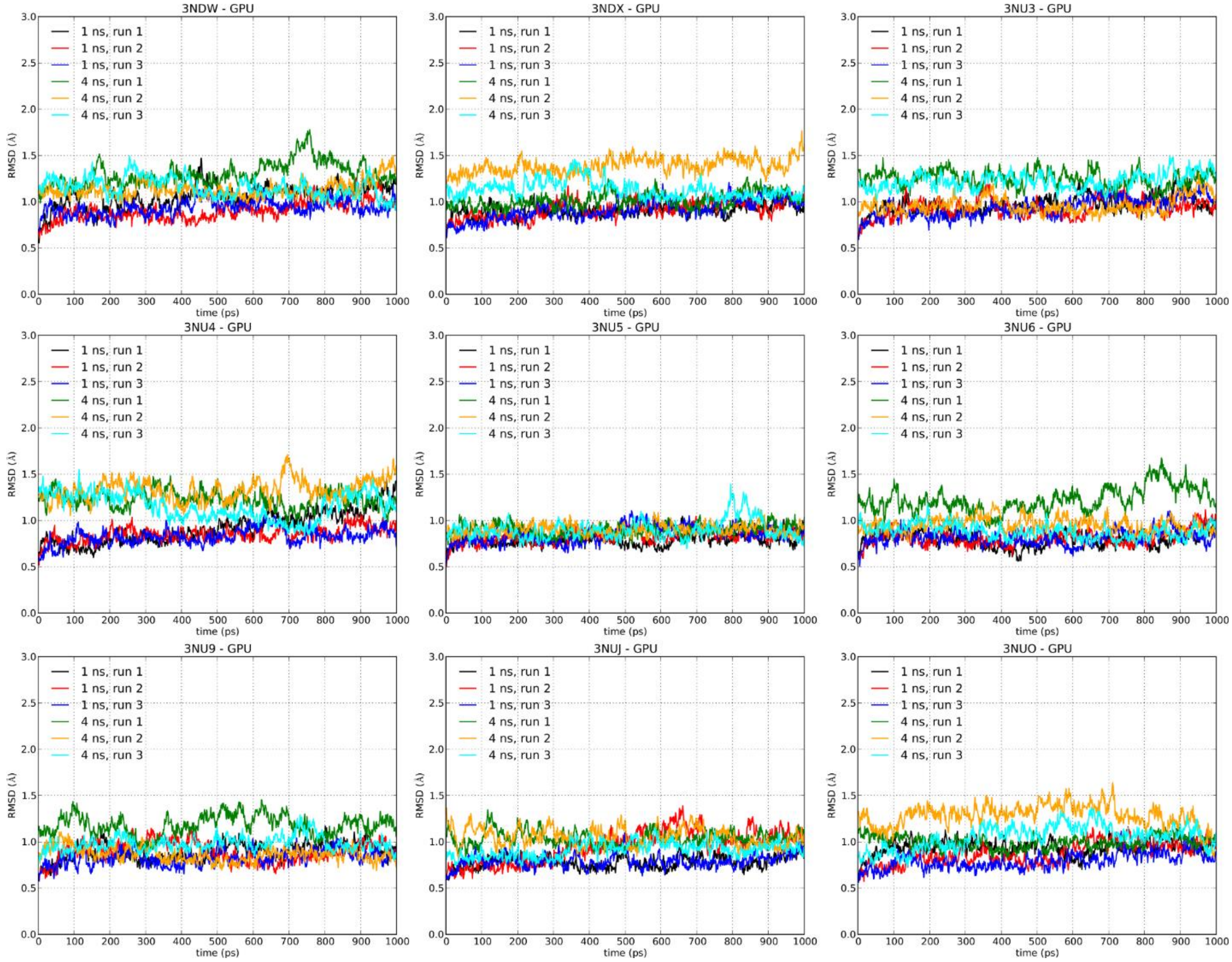

Figure S7. Comparison of RMSD values of the 1st ns and 4th of GPU MD simulations of the HIV1-protease system.

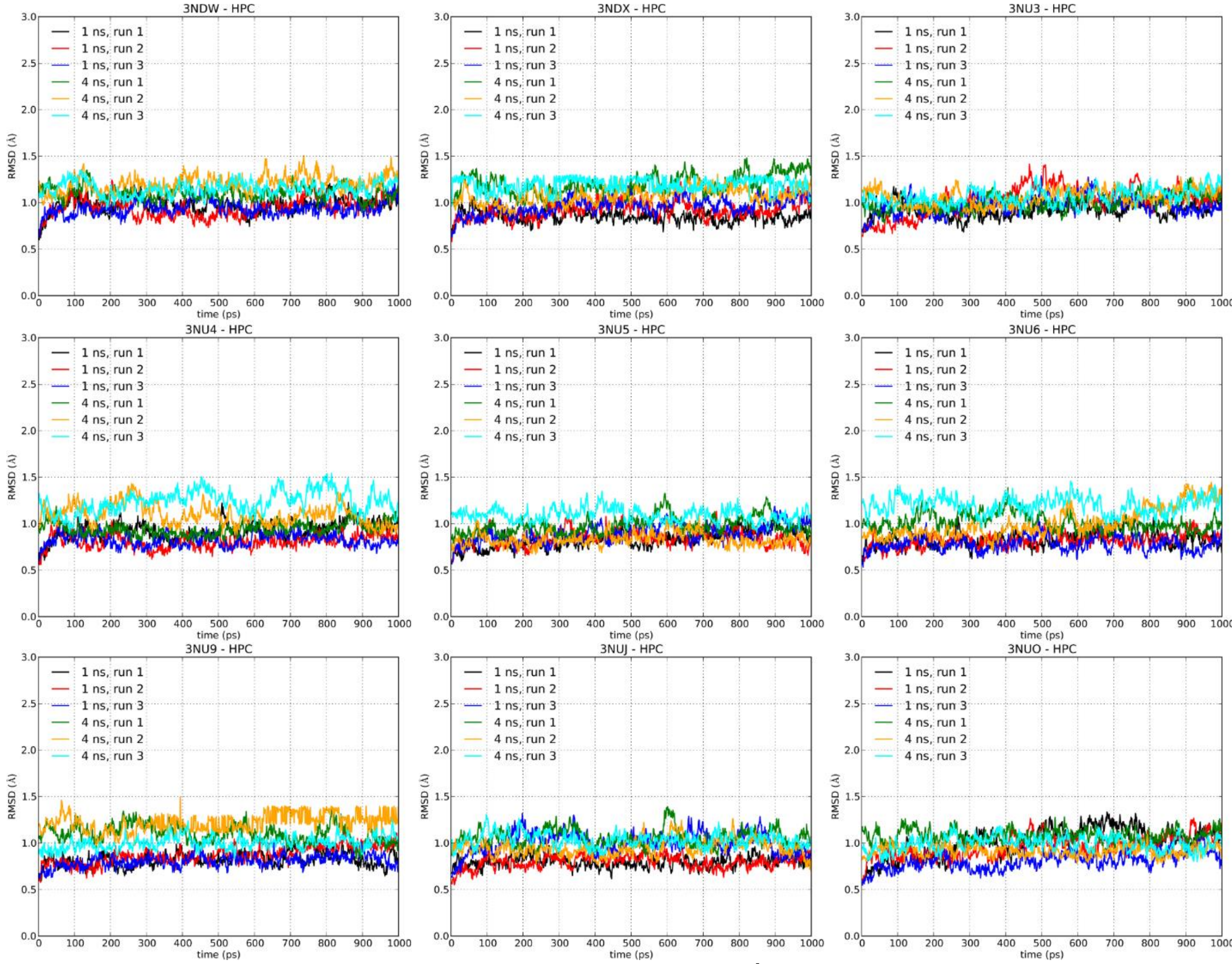

Figure S8. Comparison of RMSD values of the 1st ns and 4th of HPC MD simulations of the HIV1-protease system.

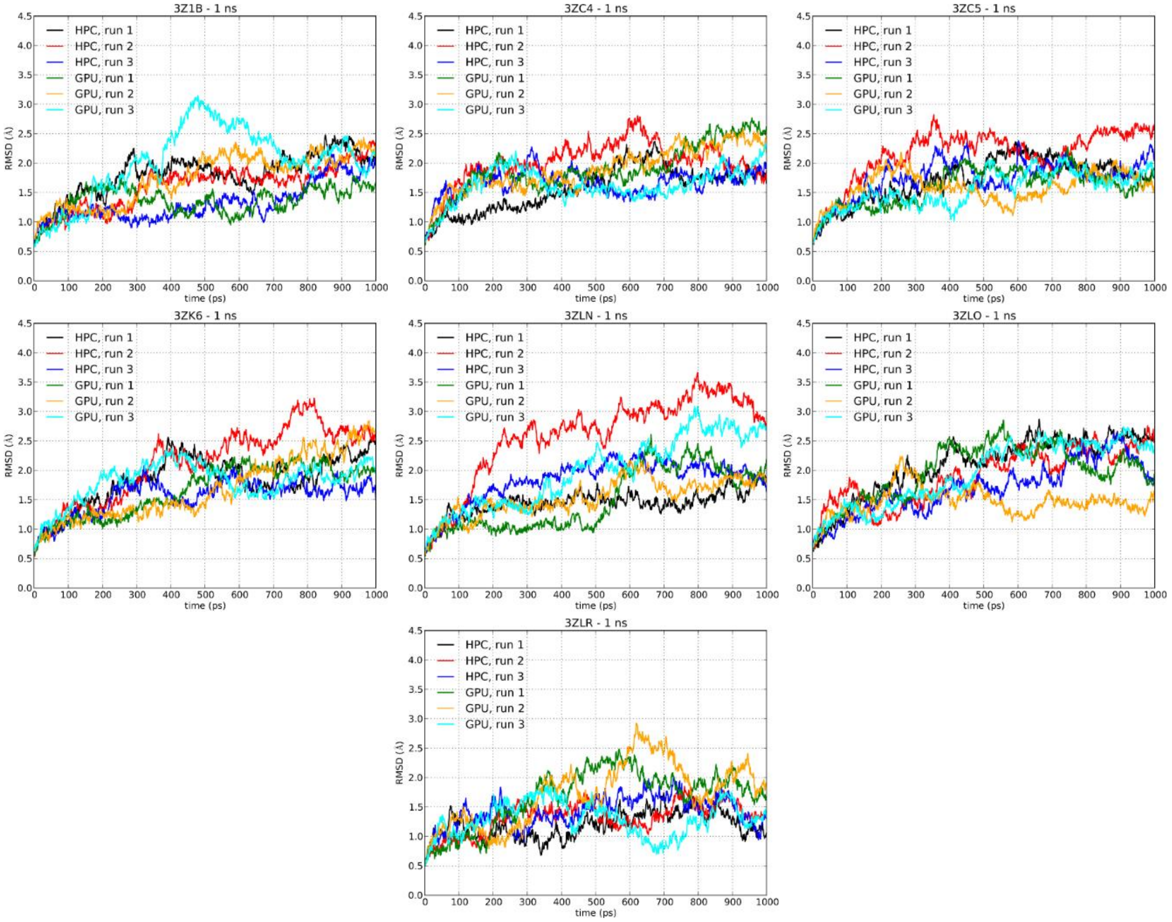

Figure S9. Comparison of RMSD values of the 1st ns of MD simulations of the Bcl-X_L system obtained by using GPU or CPU hardware.

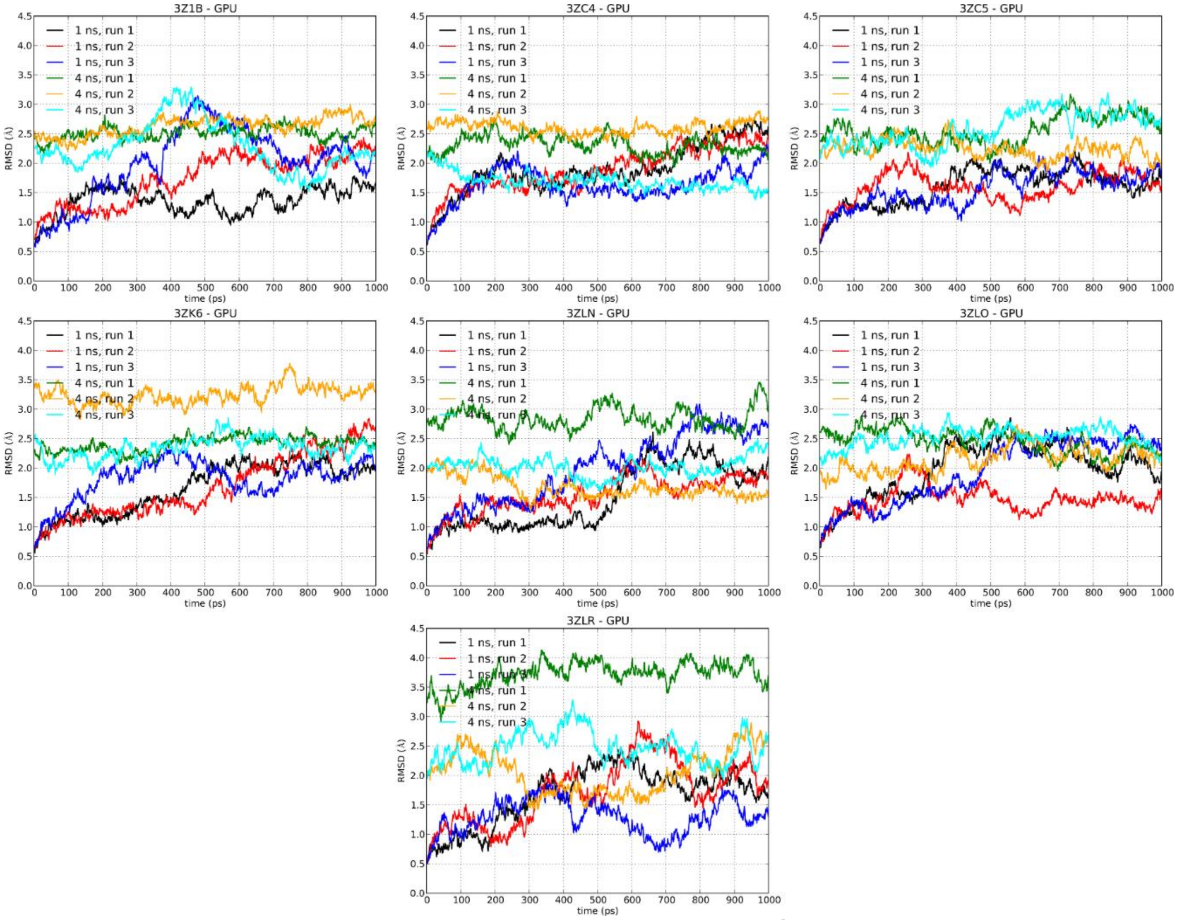

Figure S10. Comparison of RMSD values of the 1st ns and 4th of GPU MD simulations of the Bcl-X_L system.

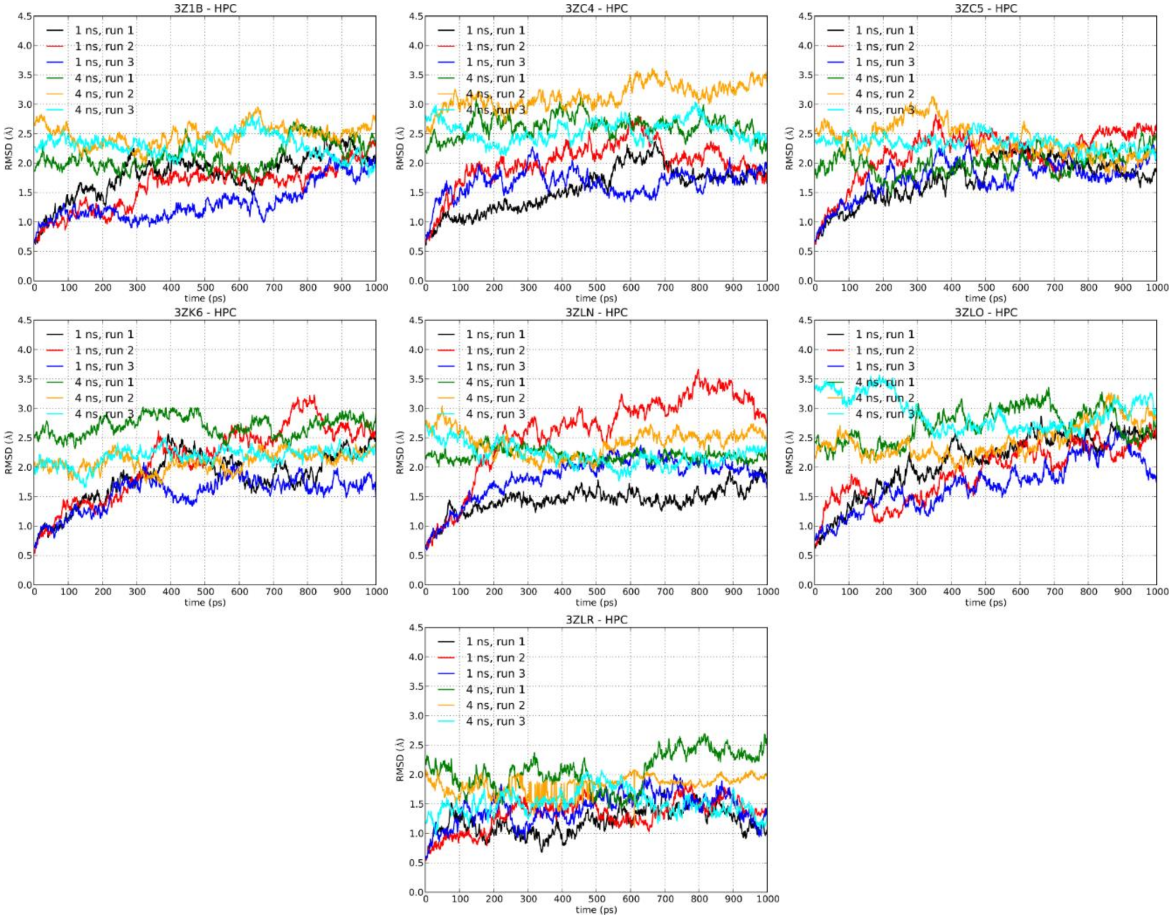

Figure S11. Comparison of RMSD values of the 1st ns and 4th of GPU MD simulations of the Bcl-X_L system.

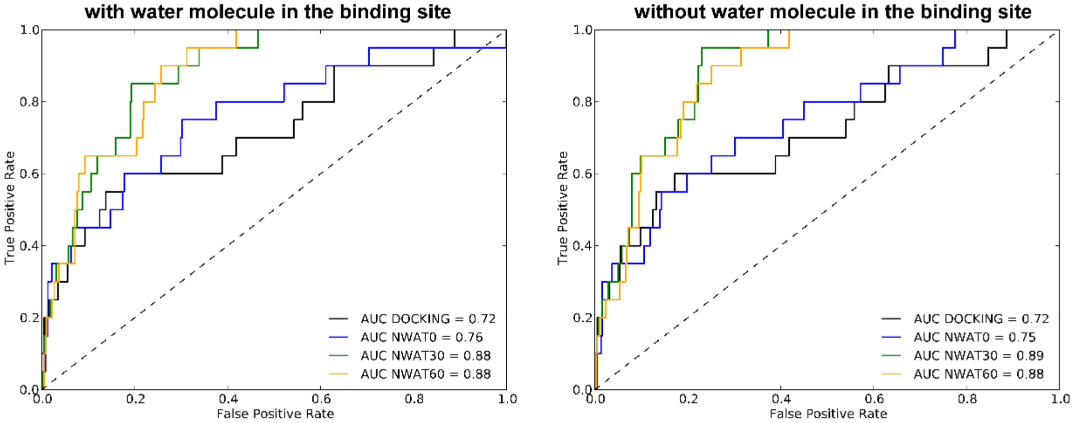

Figure S12. AUC of the ROC curves obtained from the docking experiments on AmpC by including (left) or not including (right) a water molecule in the binding site (x,y,z coordinates of the water oxygen: 77.0600, 4.2040, 24.8570)

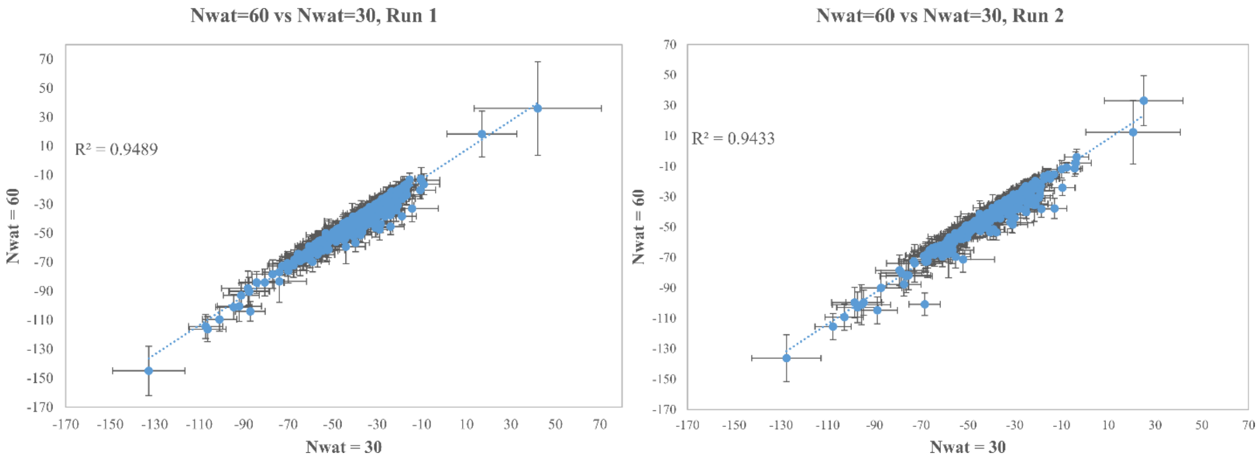

Figure S13. Ligand-to-ligand inter-method correlation between Nwat = 60 and 30 (results from two repetitions)

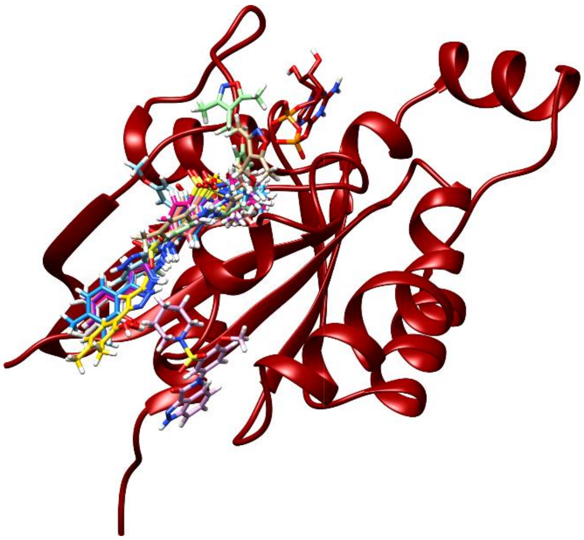

Figure S14. Docked poses of the active ligands from the docking.

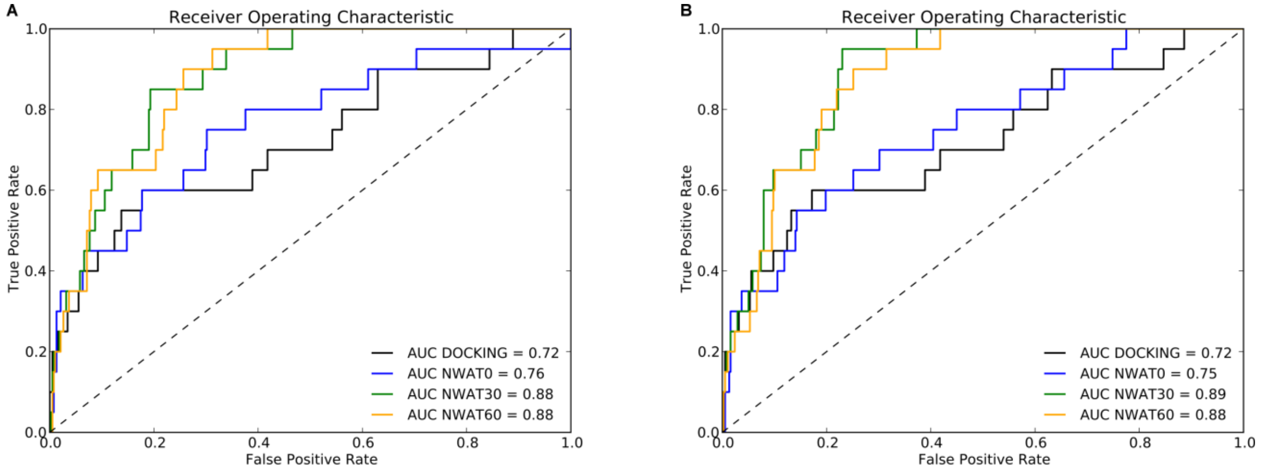

Figure S15. ROC AUCs obtained from the two repetitions of the AmpC VS

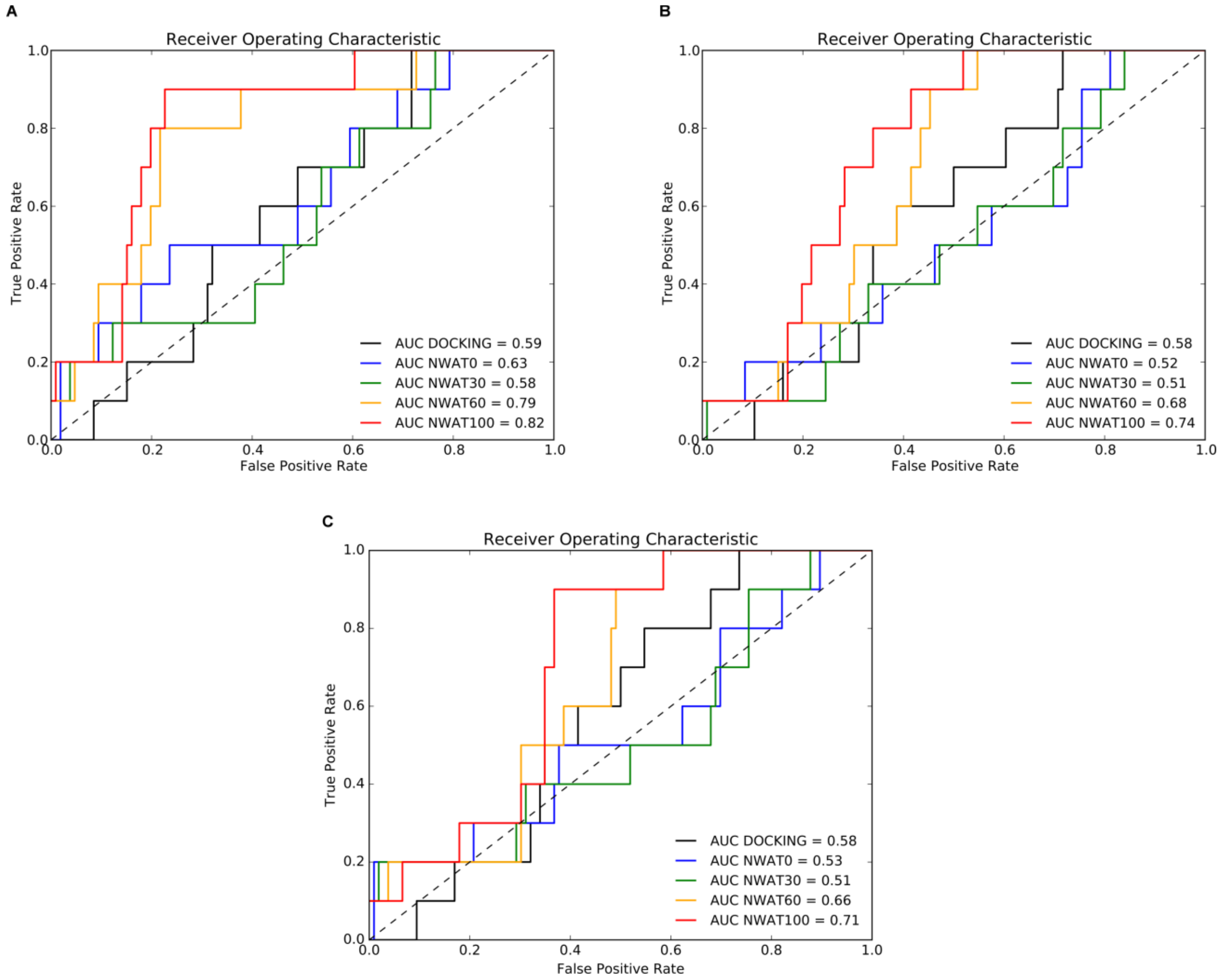

Figure S16. ROC AUCs obtained from the three repetitions of the Rac1 VS

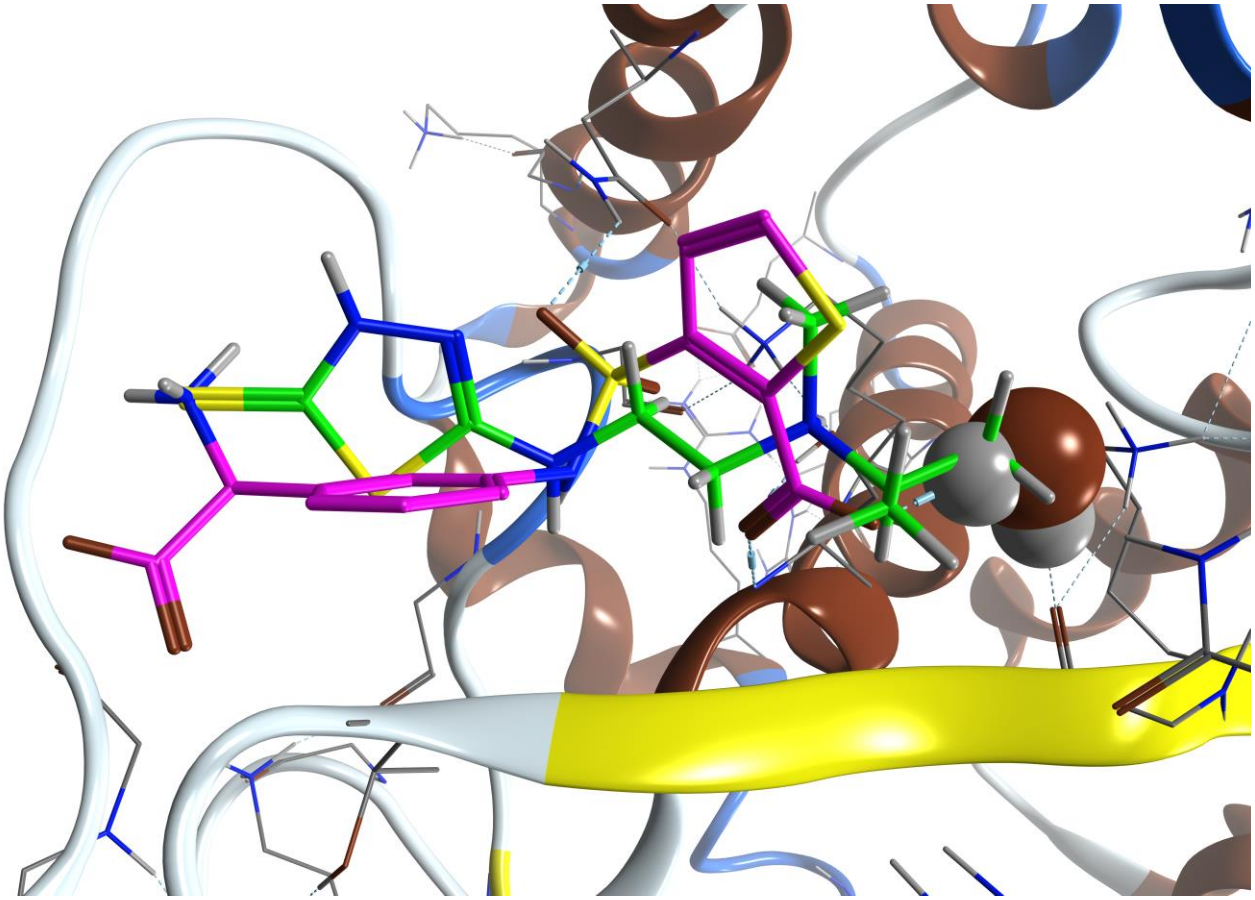

Figure S17. Predicted binding mode for the AmpC decoy 088 (green carbon atom). The crystallographic ligand (purple carbon atoms) is shown for comparison. It can be observed the overlap of the isopropyl moiety with the crystallographic water (represented as van der Waals spheres), which was not considered during docking.

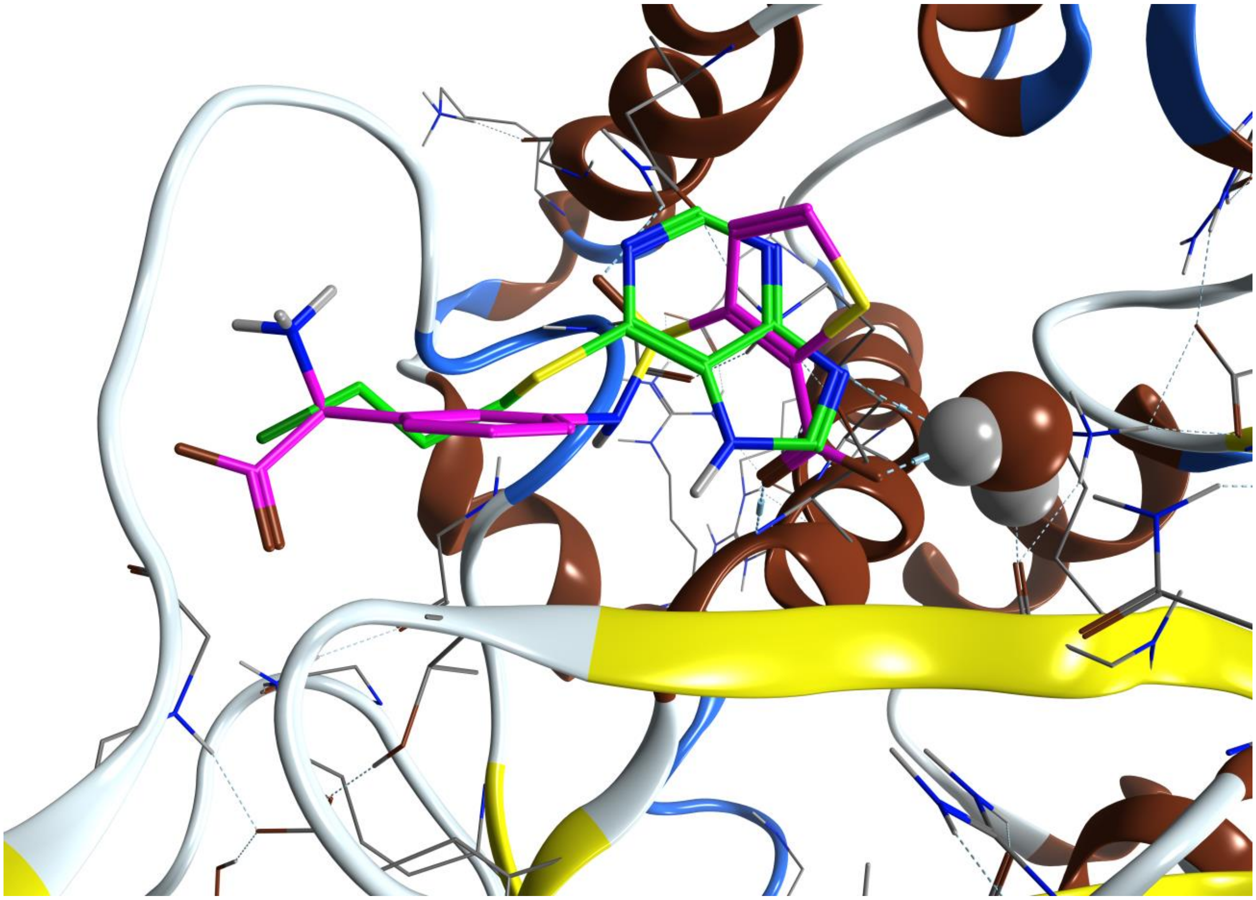

Figure S18. Predicted binding mode for the AmpC decoy 179 (green carbon atom). The crystallographic ligand (purple carbon atoms) is shown for comparison. It can be observed that the hydrophobic chloropropyl group is solvent-exposed and overlaps the hydrophilic amino acidic moiety of the crystallographic ligand.

Table S46. AmpC VS docking scores and Nwat-MMGBSA relative binding energies (kcal/mol) computed with Nwat = 0, 30, 60 and 100 (first repetition).

		Nwat=0		Nwat=30		Nwat=60	
ligand	docking score	ΔG	St.Dev.	ΔG	St.Dev.	ΔG	St.Dev.
ligand001	-70.9203	-21.0735	5.5033	-73.9765	12.205	-83.3602	14.4824
ligand002	-52.8023	-12.375	2.2397	-31.2734	4.9189	-35.4741	5.663
ligand003	-63.568	-32.9507	6.7406	-58.9545	6.9509	-58.7278	6.9506
ligand004	-49.2289	-22.449	2.8558	-87.5956	8.7532	-90.0181	9.4846
ligand005	-60.7876	-17.4622	2.123	-28.9397	3.2136	-29.5881	3.267
ligand006	-69.7475	-27.5147	3.1351	-36.7403	3.5492	-35.728	3.491
ligand007	-67.2227	-18.8992	3.1403	-38.5383	5.0104	-40.4554	4.8999
ligand008	-67.4324	-30.7253	2.9143	-27.9058	4.1606	-26.4221	4.1018
ligand009	-71.5044	-15.3561	1.9695	-31.08	4.4463	-35.4681	4.45
ligand010	-57.014	-10.9602	3.1057	-27.1359	4.203	-30.7341	3.2705
ligand011	-51.615	-13.1361	2.1058	-29.7222	3.2385	-30.4525	3.227
ligand012	-67.7158	-12.2916	3.103	-29.7997	4.6412	-36.8853	4.5655
ligand013	-64.7458	-17.529	2.6516	-27.3412	4.5138	-26.6888	4.5241
ligand014	-50.2738	-22.7554	2.6469	-25.3601	3.2633	-25.4289	3.2718
ligand015	-64.5819	-23.7584	3.7434	-25.9093	3.9325	-24.9329	4.0365
ligand016	-65.9854	-7.2767	2.7361	-22.7931	5.5771	-27.7493	5.4688
ligand017	-54.4025	-9.4167	1.7745	-23.9684	3.3675	-24.7922	3.7138
ligand018	-57.6642	-15.7799	2.871	-33.2218	4.2738	-34.4181	4.2651
ligand019	-58.983	-9.4859	2.4348	-27.6443	5.3859	-30.9142	5.5816
ligand020	-61.6109	-22.865	2.8673	-40.2357	4.466	-40.2828	4.3537
ligand021	-59.0468	-4.6855	2.5679	-20.9018	3.6088	-20.5938	3.9752
ligand022	-51.5421	-11.8891	2.5466	-23.5881	3.6645	-21.2736	3.6331
ligand023	-56.6875	-20.0218	2.7048	-46.014	5.2183	-49.6741	5.1036
ligand024	-72.1185	-20.0802	3.8363	-34.9917	4.8989	-37.6168	4.5094
ligand025	-78.3615	-12.4194	3.3628	-37.5107	5.1049	-51.4567	4.7963
ligand026	-59.9882	-15.9739	2.5403	-39.2298	5.7759	-42.9314	4.5022
ligand027	-72.4351	-4.8171	3.637	-14.3679	11.7948	-33.0381	9.1245
ligand028	-54.862	-23.2832	2.9524	-45.8982	5.0522	-49.952	4.9607
ligand029	-48.2035	-18.353	2.5698	-42.9498	4.8859	-51.2304	4.5306
ligand030	-67.4843	-25.1185	5.1815	-48.1643	5.074	-54.902	4.5405
ligand031	-63.9661	-24.8672	4.0515	-44.7328	5.247	-47.095	4.8027
ligand032	-9.74526	-29.4188	2.7175	-49.0585	4.5977	-51.4505	4.6742
ligand033	-75.3706	-14.3537	3.5264	-45.0433	5.9569	-54.7583	5.036
ligand034	-66.9673	-20.3755	3.2328	-32.5405	4.2994	-35.2295	4.2464
ligand035	-73.0725	-17.5083	3.3349	-36.8003	4.848	-39.5435	4.4498
ligand036	-65.9396	-13.9793	1.7604	-32.5657	3.9026	-38.4198	3.8236

ligand037	-71.2235	-25.9451	3.7584	-39.3214	4.5241	-39.0621	4.4072
ligand038	-54.8353	-23.2906	2.8611	-52.7159	5.2633	-54.3741	5.1833
ligand039	-66.2268	-18.5945	2.958	-39.9195	5.202	-42.2876	4.9119
ligand040	-57.2192	-16.0264	3.2476	-28.2016	5.3163	-27.3897	5.2568
ligand041	-60.2821	-26.0073	3.1028	-60.1465	5.3906	-68.2412	5.6036
ligand042	-60.8283	-12.5355	2.5303	-29.8003	4.619	-37.9374	4.574
ligand043	-73.801	-14.0976	2.3509	-29.1319	3.332	-30.6463	3.3511
ligand044	-65.5975	-15.2943	2.2595	-29.9154	4.7471	-29.3211	4.8819
ligand045	-59.6756	-7.3713	1.9576	-29.9671	4.3738	-35.4475	4.4937
ligand046	-55.7659	-46.3456	6.6061	-106.278	8.2894	-116.306	8.6858
ligand047	-77.6478	-20.4234	2.4314	-39.2315	4.8008	-48.6753	4.8651
ligand048	-77.2277	-18.0223	2.6394	-42.168	5.1165	-50.811	5.0825
ligand049	-59.4425	-18.5662	2.8289	-25.5105	3.8533	-25.1375	3.805
ligand050	-52.8139	-16.4217	2.5244	-40.7049	5.2331	-52.3279	5.0677
ligand051	-65.4548	-11.4596	3.6054	-33.061	5.0224	-35.4256	4.9348
ligand052	-61.9334	-19.5464	4.9277	-63.6376	6.8322	-68.0125	7.2953
ligand053	-69.6871	-8.0687	2.7326	-32.6701	6.0743	-40.1198	5.6983
ligand054	-78.755	-6.9938	2.8893	-21.5522	6.8552	-21.2346	6.8973
ligand055	-62.0796	-10.2501	2.3451	-15.4514	3.9984	-13.1324	4.4805
ligand056	-73.3657	-17.8671	2.2832	-40.9126	4.2799	-45.4594	4.2709
ligand057	-70.3457	-15.9653	3.3658	-46.8692	6.3845	-52.3935	6.2574
ligand058	-58.1784	-14.4774	2.6453	-42.3169	4.7945	-47.6861	4.9545
ligand059	-68.9453	-12.4072	3.278	-35.683	5.3413	-45.4066	4.6576
ligand060	-74.734	-19.8165	3.9091	-37.9296	3.8249	-37.5902	3.6733
ligand061	-46.3304	-22.7893	2.7028	-49.9102	4.5932	-52.4504	4.5764
ligand062	-61.3104	-23.5547	2.7334	-47.0516	4.8787	-51.4707	4.9866
ligand063	-71.8557	-14.0208	3.2802	-49.9198	4.4889	-53.1265	4.7542
ligand064	-49.7108	-25.6904	3.2098	-51.9015	4.8915	-61.2757	5.78
ligand065	-59.1384	-23.1455	3.2497	-38.6627	3.6848	-37.9428	3.7937
ligand066	-71.0738	-11.586	2.093	-34.0717	4.1546	-40.6308	4.4732
ligand067	-59.3108	-2.9908	1.5844	-28.4515	5.7364	-43.6943	5.9859
ligand068	-60.1386	-4.9624	2.5713	-31.3656	5.4993	-41.8748	5.5793
ligand069	-67.6578	-11.7313	2.3963	-41.2125	4.4789	-44.6231	4.6179
ligand070	-60.7614	-1.099	1.9277	-30.379	7.6567	-38.983	8.9225
ligand071	-57.6567	-15.9739	2.5637	-37.1659	4.4555	-38.3495	4.3777
ligand072	-58.1254	-7.7042	2.1721	-25.2246	4.7828	-32.8611	4.5641
ligand073	-70.6129	-18.8172	2.3426	-42.7502	3.7301	-44.9805	3.8484
ligand074	-62.1623	-26.149	3.3925	-42.7368	4.1161	-44.4549	3.8918
ligand075	-66.7052	-19.3367	2.341	-30.9406	5.7488	-35.3699	5.6373
ligand076	-73.3438	-26.8377	4.2482	-53.1254	6.1719	-54.1088	6.1574

ligand077	-72.1592	-5.8906	2.0057	-33.1036	4.0987	-35.825	4.3062
ligand078	-71.3268	-18.4647	2.3534	-46.41	5.6823	-48.7022	6.0268
ligand079	-74.7591	-17.9632	3.1572	-35.5418	5.1808	-43.4968	4.8824
ligand080	-69.6612	-16.8874	3.4169	-35.0066	4.2755	-41.1789	4.1015
ligand081	-71.7484	-13.0774	2.6611	-38.7228	4.1238	-42.3767	4.3317
ligand082	-61.2978	-26.3595	6.2414	-33.5279	5.3501	-34.0035	4.7639
ligand083	-59.3795	-9.9978	2.0301	-29.6594	4.8753	-35.776	4.7232
ligand084	-54.097	-23.7635	2.3624	-37.9415	4.9055	-37.9585	4.9931
ligand085	-63.4112	-28.8462	3.7012	-46.8929	5.6273	-53.0954	4.764
ligand086	-64.0652	-24.1039	3.3229	-27.5051	5.1579	-27.3239	4.9696
ligand087	-74.9419	-23.9756	2.8688	-52.3631	5.2335	-52.5727	5.3233
ligand088	-58.0237	-58.1458	11.1199	17.0427	15.7403	18.3456	15.8193
ligand089	-59.8896	-18.2351	2.6613	-36.9802	3.8054	-37.2214	3.8015
ligand090	-53.3426	-12.1541	2.6997	-25.0527	3.3398	-26.4884	3.077
ligand091	-62.2655	-7.7255	4.5078	-20.7097	7.2178	-30.8593	5.6077
ligand092	-61.3196	-20.8823	3.8516	-48.4652	5.353	-55.8779	5.1915
ligand093	-72.3266	-6.5243	2.2148	-26.197	5.9291	-36.8082	5.3705
ligand094	-74.3022	-16.6037	3.7161	-38.1309	4.845	-39.3937	4.8086
ligand095	-67.3977	-11.013	3.4533	-24.2505	4.6258	-27.6471	4.0534
ligand096	-66.3829	-13.5316	3.0759	-31.738	5.3415	-36.2814	4.9797
ligand097	-67.3384	-16.6438	3.5108	-35.6906	4.4135	-37.8931	4.3018
ligand098	-66.2933	-23.4419	3.2269	-37.9705	5.6931	-42.048	6.0788
ligand099	-73.9308	-22.7118	3.2814	-48.1406	6.2709	-48.8231	6.3252
ligand100	-69.3473	-28.4515	2.6137	-67.2742	6.3943	-68.3675	6.688
ligand101	-63.5795	-29.112	2.3626	-64.1095	5.7418	-66.7231	5.7125
ligand102	-63.7439	-13.8578	2.528	-28.8887	3.5384	-30.412	3.3854
ligand103	-69.7165	-12.8003	2.9752	-37.532	4.5665	-43.9415	4.7423
ligand104	-70.2701	-14.839	3.0236	-35.9325	5.2444	-38.2532	5.0627
ligand105	-66.0701	-28.6886	4.375	-40.8876	4.5482	-38.5822	4.5868
ligand106	-52.2063	-25.7539	2.5265	-37.4728	3.2647	-37.4076	3.2326
ligand107	-69.2833	-13.1832	2.391	-34.6379	4.9735	-38.1388	4.7287
ligand108	-70.5368	-22.0712	3.2283	-37.1055	4.247	-37.2245	4.1329
ligand109	-59.8842	-21.3823	3.9751	-37.4043	6.8763	-38.2036	6.6077
ligand110	-64.4591	-14.8865	3.0916	-25.6238	4.8976	-31.6355	4.5349
ligand111	-67.6769	-31.2735	3.416	-48.8912	3.4145	-48.7255	3.4119
ligand112	-60.1293	-13.7035	2.3875	-37.6689	6.1445	-41.1534	6.4652
ligand113	-62.856	-10.9394	3.08	-24.2261	4.2162	-30.8907	3.9605
ligand114	-71.7494	-21.1295	2.9449	-62.2891	7.3226	-65.5251	7.3669
ligand115	-59.7655	-8.5944	3.5732	-24.8125	3.3623	-26.1043	3.136
ligand116	-63.9563	-23.2668	4.6816	-76.9382	9.4384	-78.3601	9.8625

ligand117	-60.9184	-10.5846	2.4383	-36.864	6.3206	-39.245	6.769
ligand118	-69.8778	-11.5607	2.366	-33.158	6.0891	-34.726	5.3699
ligand119	-56.5269	-10.1941	2.2083	-37.2215	8.2025	-36.1398	8.5677
ligand120	-67.9796	-13.701	4.3096	-45.0468	5.6765	-47.117	5.4492
ligand121	-64.2745	-6.4395	2.5982	-28.9821	5.9634	-47.5093	7.265
ligand122	-76.5224	-26.7604	2.2327	-46.3806	4.3415	-48.2715	4.4234
ligand123	-69.1204	-22.1982	3.9858	-44.6729	8.5425	-44.6911	8.3511
ligand124	-65.9818	-13.5975	2.0402	-39.7259	4.9398	-43.7535	5.6331
ligand125	-76.5635	-27.5128	2.9111	-55.3004	5.7577	-54.838	5.7483
ligand126	-56.559	-5.1774	2.0858	-61.8732	8.9648	-66.4918	9.4267
ligand127	-64.7717	-5.4762	2.434	-44.0609	8.6876	-59.3237	11.7502
ligand128	-67.3177	-18.0654	3.0496	-44.8651	3.9001	-54.8976	4.4749
ligand129	-67.1942	-26.0253	3.3845	-43.2809	4.578	-41.7903	4.7114
ligand130	-66.9603	-8.813	1.6837	-34.4504	5.6656	-34.1762	5.9098
ligand131	-60.4024	-8.0183	2.1334	-36.7027	5.3461	-43.9515	6.1418
ligand132	-64.4603	-4.1068	1.8712	-30.7105	5.2011	-46.3062	6.42
ligand133	-63.9933	-28.0447	3.8219	-56.9335	6.8881	-58.5322	7.3254
ligand134	-67.9805	-22.2888	4.2584	-38.5257	3.9776	-38.9583	3.7893
ligand135	-64.9548	-26.2629	2.7554	-52.9438	4.5813	-51.5265	4.5376
ligand136	-57.0219	-12.8938	3.2541	-34.2165	6.2833	-35.1906	6.5981
ligand137	-65.1983	-19.0811	2.5326	-43.4466	4.6102	-45.1589	4.3869
ligand138	-56.8348	-21.3134	2.6695	-44.5697	4.0129	-44.3266	4.0962
ligand139	-76.3645	-10.9957	2.7371	-87.0629	6.6739	-104.01	6.7161
ligand140	-56.8394	-30.2281	4.2369	-65.8742	7.1152	-66.586	6.9872
ligand141	-48.9406	-8.8193	6.3755	-19.3696	9.1029	-24.7261	9.2029
ligand142	-57.0461	-34.1608	4.8	-65.5992	5.7447	-64.3774	5.6967
ligand143	-46.7731	N.C.[a]	N.C.	N.C.	N.C.	N.C.	N.C.
ligand144	-48.634	-11.3008	2.4494	-20.0383	4.6914	-19.548	4.6824
ligand145	-51.9429	-8.6299	1.594	-20.4152	2.6186	-20.4491	2.5082
ligand146	-10.2936	-25.0539	2.7329	-43.0878	3.9332	-45.9318	3.9303
ligand147	-14.5735	-27.7923	3.1104	-43.6805	4.3546	-44.7539	4.1073
ligand148	-53.4639	-22.7106	2.6447	-67.6424	7.2292	-72.3342	8.1724
ligand149	-57.9496	-22.4331	2.7199	-57.3421	5.9767	-57.9851	5.931
ligand150	-72.5411	-34.8862	7.5047	-63.9587	7.309	-65.0804	7.4556
ligand151	-49.398	-11.6664	3.2739	-21.9278	4.2838	-21.5417	3.9873
ligand152	-64.9399	-25.8227	4.5807	-30.7872	4.8292	-29.947	4.5259
ligand153	-57.8529	-9.899	1.747	-18.5574	4.1369	-19.0035	4.376
ligand154	-56.663	-7.0777	2.8903	-22.2051	4.2664	-25.2578	3.4253
ligand155	-61.1707	-25.1039	5.2952	-41.2699	5.4217	-44.3384	4.9008
ligand156	-62.4571	-13.5639	2.0695	-35.642	4.5337	-45.0307	4.6463

ligand157	-59.2612	-16.1505	2.8485	-17.1249	3.294	-16.2868	3.2998
ligand158	-56.4781	-13.4357	1.9941	-30.448	3.3473	-33.0027	3.151
ligand159	-60.2436	-16.5121	3.4145	-25.9267	4.4326	-25.5393	4.3233
ligand160	-55.9052	-12.5023	2.7328	-24.0483	4.366	-23.1029	4.5805
ligand161	-69.1778	-22.6861	2.8152	-38.1956	4.2913	-38.9248	3.9625
ligand162	-62.8588	-6.7878	2.7123	-24.898	5.5726	-34.12	4.4075
ligand163	-74.6895	-20.3915	2.3875	-39.496	3.4611	-43.2879	3.2451
ligand164	-68.5209	-10.6742	4.8846	-30.3826	5.0588	-36.9255	4.2296
ligand165	-69.3089	-20.0627	2.2835	-21.0938	2.6128	-20.7375	2.6651
ligand166	-79.9142	-19.5202	3.6997	-45.0312	4.8394	-45.5353	4.6524
ligand167	-63.478	-5.2245	2.0335	-17.5959	3.8246	-24.6564	3.7176
ligand168	-61.1631	-21.1082	2.7806	-33.7955	4.2168	-33.1759	4.4548
ligand169	-71.6199	-7.2847	1.8159	-20.0259	4.2515	-26.3637	4.8115
ligand170	-73.7881	-27.8959	3.3293	-41.0789	4.1241	-40.2488	4.0609
ligand171	-66.1003	-9.7904	4.8106	-25.9792	6.8146	-29.9411	5.8724
ligand172	-75.1149	-19.9938	3.319	-49.9964	5.8396	-51.8761	5.7312
ligand173	-59.3353	-23.0202	3.0658	-39.4155	4.6046	-39.8081	4.6889
ligand174	-59.0864	-16.4299	3.4422	-59.0487	7.3939	-69.8096	6.9799
ligand175	-58.314	-25.3725	4.7069	-58.8792	5.3481	-59.3407	5.4889
ligand176	-56.7707	-1.8751	1.9494	-18.8231	5.8757	-26.4222	6.5943
ligand177	-64.2868	-15.0159	2.6012	-36.9827	4.5452	-39.8631	4.3537
ligand178	-56.6888	-17.1289	2.7963	-43.5759	7.0921	-44.7641	7.353
ligand179	-70.4442	-51.3429	32.5702	42.1334	28.5713	35.9547	32.2512
ligand180	-63.7411	-17.1326	5.2238	-62.9475	7.5652	-67.7415	7.5802
ligand181	-65.7001	-36.8695	5.1799	-52.5502	6.9064	-52.0103	6.7319
ligand182	-70.8937	-20.8008	2.5987	-51.1267	4.7086	-53.3749	4.4505
ligand183	-52.1796	-13.1449	3.4487	-28.8064	3.0846	-30.3125	3.1787
ligand184	-73.2016	-13.1739	3.0689	-31.653	4.9121	-35.4418	5.4295
ligand185	-67.3188	-11.5485	2.4726	-24.3334	4.4598	-28.0656	4.2824
ligand186	-66.8783	-25.7371	2.8559	-35.2515	4.2988	-34.6833	4.236
ligand187	-67.6387	-10.8571	1.8155	-27.2057	4.3283	-31.4312	4.6246
ligand188	-62.3996	-6.4654	2.8527	-28.4051	5.18	-32.8543	5.3793
ligand189	-64.8109	-15.4189	2.2997	-37.9264	4.9656	-48.708	4.7055
ligand190	-57.8165	-21.1691	3.2791	-29.3261	4.0087	-29.1364	4.0367
ligand191	-79.7168	-46.123	4.4454	-72.6894	6.5824	-72.6266	6.5806
ligand192	-76.8963	-38.3967	6.1233	-65.7506	5.8358	-65.8462	5.842
ligand193	-63.7203	-7.8033	2.8725	-20.8565	5.5539	-25.147	4.5778
ligand194	-69.4294	-10.4809	2.3617	-22.8462	5.9693	-24.1586	6.2223
ligand195	-53.2197	-28.8689	2.8385	-54.5666	4.3629	-61.3114	4.5289
ligand196	-67.046	-20.1102	2.9626	-36.5966	4.3013	-36.3284	3.919

ligand197	-63.7956	-11.6409	1.8854	-26.465	4.7603	-37.8774	4.6515
ligand198	-70.5188	-13.2814	2.7791	-32.4145	4.385	-33.7524	4.1791
ligand199	-67.9146	-20.736	2.7947	-34.4795	4.5589	-33.8366	4.5007
ligand200	-71.2994	-11.6572	2.1841	-33.3921	3.9938	-37.3835	3.9372
ligand201	-66.3797	-18.7674	2.8653	-37.5998	4.4125	-37.2656	4.4289
ligand202	-78.068	-9.4259	2.6534	-40.7696	7.3097	-43.0382	8.867
ligand203	-72.5487	-17.3333	2.6453	-32.6377	3.8939	-35.465	3.4627
ligand204	-78.2098	-6.1651	2.2001	-23.9079	6.1955	-36.8454	5.1338
ligand205	-65.8803	-28.6449	4.1472	-46.9953	4.802	-46.8074	4.6491
ligand206	-52.7776	-12.9224	2.5765	-42.3098	4.8569	-45.0965	4.741
ligand207	-61.2254	-34.328	3.5354	-45.9448	3.6675	-45.5425	3.4801
ligand208	-64.5766	-13.6611	3.5179	-37.3004	6.6619	-40.66	4.5607
ligand209	-59.6978	-9.7162	2.7962	-28.23	5.6408	-32.1133	5.0077
ligand210	-65.0904	-20.8745	2.5426	-34.9402	3.4279	-34.4106	3.5621
ligand211	-78.2073	-30.345	3.611	-54.1674	5.2757	-58.2354	5.5337
ligand212	-63.3281	-15.4205	3.0363	-29.4227	3.5145	-30.2998	3.1152
ligand213	-67.647	-31.19	2.804	-49.6638	4.1951	-49.1241	4.1818
ligand214	-67.8396	-27.8061	4.0682	-40.6361	3.8899	-40.3139	3.9114
ligand215	-71.893	-28.1454	2.2716	-39.1514	2.7195	-38.8866	2.711
ligand216	-71.1676	-15.933	4.111	-44.2394	6.6991	-46.069	6.7544
ligand217	-47.6016	-27.4796	4.0908	-51.8482	4.7926	-56.2562	4.4999
ligand218	-72.984	-19.944	2.7821	-59.6746	6.441	-62.1944	6.5827
ligand219	-75.8496	-30.7224	8.4864	-55.7527	7.9433	-57.7375	7.9118
ligand220	-59.8716	-9.1659	2.5978	-23.7263	4.8404	-32.5132	4.5325
ligand221	-50.9974	-11.8898	1.8228	-25.6489	4.2472	-25.5513	3.9871
ligand222	-66.2638	-21.8056	2.5777	-35.1762	3.8736	-35.4985	3.7887
ligand223	-66.4069	-19.2904	2.4959	-37.1371	4.5396	-38.785	4.3354
ligand224	-56.0219	-27.1319	3.1854	-37.123	3.9238	-36.5379	3.9559
ligand225	-68.2101	-6.3369	3.3214	-26.535	7.2789	-38.6083	5.4452
ligand226	-73.7114	-32.9699	4.1909	-57.8433	6.7636	-57.4721	6.6257
ligand227	-69.1126	-29.3036	4.9348	-43.9389	4.9211	-43.3423	5.0379
ligand228	-67.6682	-13.2307	4.1266	-32.6304	6.6458	-37.3558	5.0129
ligand229	-45.2809	-14.3321	1.9959	-17.1186	2.0591	-16.979	2.0584
ligand230	-47.4811	-8.9305	1.839	-17.3416	2.5633	-16.5277	2.5779
ligand231	-56.1843	-13.1714	8.4831	-51.7635	10.1951	-51.2307	10.8529
ligand232	-63.9422	-17.2528	3.1863	-28.568	6.3088	-33.8454	5.3024
ligand233	-41.738	-3.2399	1.8562	-10.4455	2.6507	-12.4792	2.4997
ligand234	-61.9853	-7.1577	2.2367	-27.6706	4.455	-30.1614	4.4362
ligand235	-67.1502	-9.1115	3.4776	-9.0867	7.1202	-16.3453	7.1599
ligand236	-55.0482	-12.3233	2.0402	-27.3953	2.7565	-27.2681	2.5791

ligand237	-50.6047	-6.2756	1.5452	-18.9907	3.8394	-25.0382	3.4766
ligand238	-56.5132	-19.1742	2.0641	-31.0172	4.3451	-31.144	4.317
ligand239	-61.8579	-21.3432	2.7985	-28.7652	3.9628	-28.4016	3.8141
ligand240	-67.8444	-15.6756	2.5788	-36.57	6.894	-36.5467	7.1474
ligand241	-60.8371	-6.7836	2.4253	-64.5954	9.2973	-69.3195	10.0458
ligand242	-59.0115	-1.5144	8.8097	-38.4215	11.4511	-43.0754	12.6861
ligand243	-73.0766	-19.9739	4.1044	-52.5288	7.8475	-61.6321	7.8214
ligand244	-65.428	-27.994	2.8368	-43.0929	3.5848	-43.4779	3.4983
ligand245	-64.9728	-18.4317	2.1477	-37.544	4.1217	-38.5387	4.0544
ligand246	-70.3445	-11.6411	3.8245	-10.6879	6.9391	-20.2749	5.9844
ligand247	-66.6945	-10.2743	2.6126	-29.6524	4.6148	-33.2683	4.4928
ligand248	32.2442	-15.727	2.5318	-39.7411	6.2748	-56.7722	6.0825
ligand249	-52.7841	-11.1406	3.4815	-56.8245	7.4294	-62.5621	7.3369
ligand250	-61.6969	-16.6067	3.6062	-87.5664	9.2861	-90.4458	9.8479
ligand251	-65.6978	121.1435	8.4831	-88.1546	11.8396	-88.1135	11.8949
ligand252	-53.2483	-7.0629	2.4125	-16.1631	3.4814	-16.4186	3.9219
ligand253	-57.5191	-8.5814	2.373	-26.1542	7.2657	-25.7846	7.4483
ligand254	-71.8124	-10.4137	2.7668	-26.7684	5.8077	-33.4725	6.3985
ligand255	-47.9181	-3.8412	2.3377	-17.083	3.5436	-19.7871	2.9164
ligand256	-65.6021	-13.7219	2.732	-48.1533	5.2679	-49.7457	4.7357
ligand257	-67.4186	-14.7838	2.5965	-38.5911	4.3933	-38.2294	4.2607
ligand258	-64.6614	-7.9584	2.5868	-17.9373	6.2825	-17.1604	6.1518
ligand259	-71.2288	-14.8427	3.8757	-32.818	4.5659	-33.2489	4.0787
ligand260	-67.7256	-13.6639	3.223	-31.0864	3.5448	-31.1751	3.389
ligand261	-56.8008	-12.1589	1.7437	-30.1946	4.6597	-31.7546	4.3456
ligand262	-62.5083	-11.9536	2.4033	-32.761	3.7789	-36.5426	3.5141
ligand263	-74.4096	-16.7771	2.8802	-34.1041	5.685	-39.4309	5.5968
ligand264	-51.1618	-15.0359	1.7386	-21.4606	1.9823	-20.8915	2.0252
ligand265	-69.4886	-8.4249	3.809	-26.4138	5.6282	-29.6505	5.2242
ligand266	-62.0072	-15.9645	4.5798	-33.2804	4.695	-34.9634	4.5794
ligand267	-59.4927	-12.1002	2.368	-30.3392	4.4666	-30.8368	4.0487
ligand268	-65.5182	-10.8868	2.7848	-22.1707	4.064	-22.104	4.0983
ligand269	-67.7304	-9.7693	2.5646	-28.0334	4.4176	-30.2586	4.8849
ligand270	-64.0143	-32.2699	3.1206	-45.1684	3.4301	-43.1681	3.3381
ligand271	-69.1175	-6.7645	3.0654	-29.8546	5.4604	-43.9689	4.3639
ligand272	-72.9702	-23.3575	2.0194	-50.1284	4.357	-52.7754	4.5235
ligand273	-75.1801	-28.9857	3.3054	-46.8009	4.5373	-50.1468	4.2735
ligand274	-54.7818	-11.7122	1.8066	-34.1333	5.6106	-37.4131	5.8293
ligand275	-51.238	-7.4771	1.7953	-18.7705	3.1924	-18.6023	3.1156
ligand276	-61.6155	-28.8627	2.7181	-47.4012	4.2554	-46.6661	4.1033

ligand277	-58.0128	-16.3407	1.706	-27.8847	2.9704	-28.5341	2.7964
ligand278	-65.2554	-20.8158	2.2706	-37.6751	3.6513	-38.3856	3.4891
ligand279	-64.21	-21.2075	2.5112	-37.9437	3.4479	-37.6621	3.3321
ligand280	-71.5663	-24.7669	2.1131	-45.8283	4.0445	-45.4881	3.954
ligand281	-63.8808	-9.5522	3.0951	-36.2339	6.3469	-43.1296	6.3005
ligand282	-66.9065	-13.8036	2.4753	-37.5668	4.8775	-39.1892	5.187
ligand283	-66.9817	-11.687	4.9716	-26.8355	6.0129	-29.117	5.0411
ligand284	-65.5844	-12.4303	2.7927	-26.1905	4.8065	-28.7869	5.0054
ligand285	-60.7934	-6.3036	2.6511	-24.4371	4.9476	-25.1572	5.2002
ligand286	-66.6176	-20.6622	2.6037	-34.9587	3.8781	-36.6063	3.8866
ligand287	-58.3294	-28.616	2.8714	-27.409	3.859	-26.7698	3.8767
ligand288	-58.1062	-10.8518	3.1864	-27.9087	4.121	-26.8463	4.31
ligand289	-53.4796	-11.6067	2.9648	-28.1237	4.2079	-27.2882	4.1662
ligand290	-75.553	-13.3068	2.2563	-36.9236	4.1002	-38.6941	4.2249
ligand291	-65.8609	-7.672	3.7979	-25.9794	5.4419	-30.2345	4.2466
ligand292	-61.9178	-15.7243	2.054	-31.7327	3.3588	-31.3728	3.3936
ligand293	-69.4667	-4.2612	1.6172	-23.4293	5.0936	-27.6801	5.2623
ligand294	-63.9003	-15.5841	2.3314	-34.7938	4.4416	-37.2337	4.8324
ligand295	-60.6756	-16.1904	2.8908	-43.2105	4.3925	-43.4563	4.533
ligand296	-62.7994	-23.3038	3.35	-48.065	5.1916	-47.1473	5.2744
ligand297	-65.0764	-17.3419	2.8816	-31.7829	3.887	-31.8935	3.8436
ligand298	-60.473	-14.4786	3.3339	-24.0557	4.4785	-23.2799	4.5394
ligand299	-69.0728	-8.73	2.5304	-27.3466	4.7676	-31.0865	4.6122
ligand300	-76.7561	-25.5699	2.6408	-45.5002	4.5243	-48.1439	4.1686
ligand301	-60.7074	-15.1272	3.6841	-10.2768	8.3391	-13.523	8.7663
ligand302	-70.1492	-9.4296	3.6615	-32.8503	7.3038	-41.9771	7.3301
ligand303	-66.2763	-17.4672	5.3876	-29.6815	6.8725	-30.4875	6.5303
ligand304	-69.466	-24.3649	3.612	-56.2515	7.0272	-55.4911	6.9464
ligand305	-64.9064	-15.4437	2.7832	-36.2559	4.579	-37.4538	4.4983
ligand306	-60.6485	-9.8518	3.1702	-24.6409	4.79	-30.06	3.8973
ligand307	-54.2965	-5.9918	2.7704	-30.9901	4.1938	-30.1517	4.2736
ligand308	-55.8192	-19.8117	3.0316	-37.4146	3.8848	-36.4057	3.5888
ligand309	-67.9988	-20.2174	3.1221	-60.6255	6.4093	-59.0682	6.5702
ligand310	-75.1061	-7.0532	2.8522	-42.4082	11.3884	-45.7546	9.3672
ligand311	-56.0826	-10.4626	1.535	-31.1568	4.6568	-32.3766	4.4659
ligand312	-57.2739	-22.1036	2.7105	-34.56	4.503	-34.3421	4.4941
ligand313	-58.7657	-11.8712	2.2117	-26.8865	3.7616	-27.5136	3.5101
ligand314	-54.0883	-12.4618	2.7415	-25.9966	3.7102	-26.6254	3.7352
ligand315	-51.7358	-4.8341	2.2633	-22.0715	3.9762	-28.3251	3.8617
ligand316	-59.2035	-21.4178	3.1232	-46.4189	5.3705	-48.459	5.1284

ligand317	-56.4038	-3.4729	1.6844	-16.7474	6.8405	-23.6356	6.5466
ligand318	-64.667	-7.5075	3.5578	-33.8843	5.4636	-43.7219	6.7952
ligand319	-74.5772	-12.4875	3.0812	-38.9624	5.8829	-42.3179	6.065
ligand320	-56.0198	0.0856	0.1144	-21.5086	3.8891	-34.4054	7.527
ligand321	-63.9938	-21.7123	2.9605	-39.9689	4.2431	-39.0652	4.1039
ligand322	-58.94	-8.7918	2.6829	-36.7927	6.8004	-38.4018	6.8477
ligand323	-58.7592	-15.4459	2.2278	-36.3768	3.9309	-37.3484	3.7782
ligand324	-55.9566	-9.1872	4.2519	-65.2867	6.2991	-65.8232	6.9434
ligand325	-55.6751	-25.696	3.1051	-53.1236	4.1386	-50.0026	4.1734
ligand326	-66.8882	-6.6825	1.988	-36.6035	4.9634	-39.679	5.2302
ligand327	-64.4418	-7.1953	2.9712	-28.8742	4.4505	-29.0718	4.7743
ligand328	-54.7935	-16.0179	3.2445	-48.7324	6.1398	-48.3924	6.2444
ligand329	-63.8868	-2.3197	1.4736	-31.4364	4.5482	-37.0518	4.8182
ligand330	-54.2386	-14.7825	2.3378	-22.6235	3.1906	-22.4303	3.15
ligand331	-63.4678	-0.3748	1.1113	-18.9055	6.4324	-38.3875	5.8521
ligand332	-68.7382	-30.6904	3.844	-59.1215	5.9888	-63.8057	5.9483
ligand333	-66.0057	-15.4675	2.7508	-38.7383	4.6878	-40.3911	4.7821
ligand334	-58.7343	-24.5615	4.7556	-38.4348	5.7909	-37.6674	5.7309
ligand335	-60.5894	-8.2498	1.7347	-24.6379	3.7693	-27.1354	3.8296
ligand336	-61.9255	-16.0485	2.5742	-33.4946	4.1877	-32.1089	4.3189
ligand337	-60.8582	-15.1175	3.643	-30.5968	4.2602	-32.9787	3.6598
ligand338	-51.2814	-8.2919	1.8751	-20.7984	3.4674	-20.7859	3.3233
ligand339	-52.7959	-12.5856	5.0567	-28.5631	4.5972	-29.0737	4.508
ligand340	-64.3067	-15.5582	3.1174	-31.7916	4.0505	-32.7807	3.8691
ligand341	-60.1889	-19.888	3.9729	-80.5212	8.6497	-84.0791	9.2407
ligand342	-54.0328	-36.4057	7.7564	-31.7638	8.3675	-33.718	9.0905
ligand343	-76.3087	-15.6557	3.5296	-57.3898	6.8464	-60.9196	7.2951
ligand344	-64.5742	-33.4771	7.4111	-45.7079	8.1388	-46.3433	7.8935
ligand345	-68.3927	-30.4199	4.0112	-58.165	6.7738	-58.978	6.7711
ligand346	-58.0589	-7.2429	2.2694	-25.1904	4.6652	-27.2682	4.9061
ligand347	-52.241	-13.1993	2.2849	-35.9283	6.4681	-39.339	6.6527
ligand348	-53.7426	-9.4574	5.6033	-91.987	9.9464	-100.447	10.5183
ligand349	-63.0484	-17.6971	3.1094	-37.4864	4.1316	-36.8936	3.9083
ligand350	-66.3432	-10.6778	2.3284	-32.8861	5.8941	-35.5603	5.4559
ligand351	-65.3405	-10.2978	2.8251	-40.5417	4.8236	-43.491	4.626
ligand352	-70.6136	-29.1665	2.2834	-56.3303	5.1071	-56.6365	5.1172
ligand353	-65.4915	-9.4226	2.5864	-40.4146	6.0873	-44.4198	5.9091
ligand354	-67.2653	-18.8309	2.327	-52.983	4.4163	-54.7261	4.3362
ligand355	-63.8608	-31.5258	4.254	-55.7986	6.2321	-55.8639	6.2457
ligand356	-55.4477	-2.9538	2.6273	-24.1427	5.0721	-37.5909	4.2955

ligand357	-55.3212	-4.2628	1.8668	-17.694	5.1561	-18.6696	4.847
ligand358	-62.2785	-7.4946	1.9198	-29.3235	4.0544	-32.2147	4.862
ligand359	-57.2271	-16.4595	2.7353	-31.6165	4.1614	-32.1805	4.3735
ligand360	-54.5854	-2.3985	2.7135	-26.0463	5.2333	-29.615	6.3198
ligand361	-73.0095	-20.6918	4.0746	-70.0002	8.2872	-76.0631	8.4627
ligand362	-62.3862	-16.3424	2.0336	-33.0645	5.7695	-34.8397	5.0561
ligand363	-64.4631	-4.7412	2.6598	-45.2779	7.7253	-46.1567	8.74
ligand364	-63.7268	-26.7609	9.6445	-41.2919	12.4895	-41.5803	12.2126
ligand365	-61.2968	-10.7671	3.1966	-23.6228	7.466	-24.3019	7.1445
ligand366	-57.057	-4.7301	2.1451	-24.8958	4.4739	-24.0861	4.7311
ligand367	-73.3829	-10.2838	2.3255	-36.3636	5.6826	-38.0994	5.5338
ligand368	-63.932	-11.4661	3.053	-44.3822	5.001	-53.8255	5.461
ligand369	-65.2814	-3.7286	2.1882	-24.0669	6.1658	-45.4261	5.5887
ligand370	-64.5362	-19.7608	3.6997	-57.1936	6.1522	-57.034	6.3823
ligand371	-63.1388	-17.4049	2.9458	-36.2933	3.6903	-35.6676	3.8569
ligand372	-63.5674	-10.5441	2.7875	-37.0685	4.4732	-40.8392	4.6088
ligand373	-60.2451	-14.0462	5.1034	-35.9986	4.8444	-36.7666	4.7219
ligand374	-52.5004	-2.7027	1.9534	-16.7901	3.855	-17.7469	3.924
ligand375	-57.0998	-10.1238	1.656	-26.6748	3.6158	-27.3025	3.798
ligand376	-56.48	-15.0418	2.0789	-25.0989	2.8035	-25.0253	2.8343
ligand377	-48.7478	-16.3638	2.6429	-27.701	4.5795	-29.6395	4.5892
ligand378	-50.7134	-28.2675	6.3006	-70.3391	8.2394	-71.3132	8.5233
ligand379	-56.3774	1.3138	3.8841	-132.675	16.2055	-145.041	16.9576
ligand380	-70.465	-20.4098	3.2985	-58.6076	6.5906	-60.4268	7.4441
ligand381	-65.1976	-18.4052	2.9355	-54.617	7.0095	-62.2174	6.5956
ligand382	-73.2092	-25.9443	3.5248	-61.8842	6.9756	-63.2502	7.4611
ligand383	-76.5039	-35.5465	5.8672	-70.3314	6.6854	-70.7843	6.7085
ligand384	-72.0449	-24.7946	3.8339	-59.3636	7.1188	-64.3977	7.3712
ligand385	-60.8048	-24.9017	3.7513	-47.5371	5.5288	-46.91	5.5011
ligand386	-53.5783	-11.4759	6.6134	-50.0421	7.9924	-50.4765	7.6962
ligand387	-79.5088	-50.5868	3.2857	-91.1603	8.0343	-92.9644	8.3777
ligand388	-79.0276	-42.4267	5.4037	-94.5152	8.154	-101.103	8.6886
ligand389	-85.3408	-45.1086	5.3369	-107.102	7.6834	-114.361	8.3501
ligand390	-78.1557	-50.9505	5.7516	-100.879	7.7607	-109.52	8.1946
ligand391	-75.0963	1.7092	3.6304	-36.7735	6.842	-49.2418	7.352
ligand392	-80.4299	-29.8848	4.4779	-73.9536	7.6399	-76.9669	7.7527
ligand393	-74.8032	-29.3996	5.2021	-63.8901	7.1701	-64.8882	7.025
ligand394	-65.6247	-13.1624	2.1062	-56.8068	7.6821	-64.8237	8.5745
ligand395	-71.7144	-20.5341	3.863	-47.3656	5.9897	-48.8866	6.5972
ligand396	-60.7966	-15.0728	2.3988	-42.3898	5.9789	-44.6499	6.1228

ligand397	-63.2456	-41.6626	4.3989	-48.0525	4.8662	-47.8904	4.8
ligand398	-55.7726	-21.4896	2.1127	-39.824	4.3754	-40.1787	4.3866
ligand399	-62.416	-71.3809	5.1013	-84.2218	7.8861	-84.0609	7.6188

a Parametrization did not converge

Table S47. AmpC VS docking scores and Nwat-MMGBSA relative binding energies (kcal/mol) computed with Nwat = 0, 30, 60 and 100 (second repetition).

		Nwat=0		Nwat=30		Nwat=60	
ligand	docking score	ΔG	St.Dev.	ΔG	St.Dev.	ΔG	St.Dev.
ligand001	-70.8736	-33.4501	5.1492	-76.4401	11.2841	-82.3722	10.8057
ligand002	-52.8033	-15.03	1.9011	-32.406	4.566	-35.8054	4.7337
ligand003	-63.5714	-30.3476	4.408	-58.7217	6.7842	-58.5501	6.892
ligand004	-49.2362	-18.583	3.0702	-87.2196	8.9743	-90.0191	9.6044
ligand005	-60.7895	-17.4807	1.8583	-27.9053	3.2855	-28.7063	3.2045
ligand006	-69.8091	-19.0711	3.7744	-33.4097	4.5651	-36.183	4.291
ligand007	-67.2144	-18.811	3.2012	-40.8117	4.7628	-43.3573	4.4497
ligand008	-67.4472	-20.5546	3.2084	-22.0654	5.1411	-22.1036	5.0512
ligand009	-71.4682	-13.2921	1.6844	-30.0612	3.6414	-35.4811	3.8149
ligand010	-57.0116	-13.8966	3.0562	-30.0721	4.4645	-30.9442	3.9979
ligand011	-51.6189	-12.8049	2.0782	-26.1217	3.6782	-27.4325	3.673
ligand012	-67.7418	-6.0637	2.868	-21.1011	5.3462	-34.862	4.5209
ligand013	-65.054	-15.0534	3.8754	-25.7983	4.7814	-25.7788	4.6263
ligand014	-50.2728	-11.3099	1.7728	-20.4822	4.9701	-20.682	4.8272
ligand015	-64.5804	-23.4055	2.8196	-24.469	4.2516	-23.5745	4.1469
ligand016	-65.9719	-12.2645	2.762	-31.743	4.7167	-35.6167	4.5658
ligand017	-54.402	-12.5604	2.4495	-24.6758	4.237	-24.5704	4.2396
ligand018	-57.662	-12.2127	2.1431	-31.8531	3.3923	-32.0227	3.4257
ligand019	-58.9827	-10.2824	2.0664	-25.7926	4.1894	-27.0694	4.8674
ligand020	-61.5967	-10.1235	1.6413	-31.539	4.0124	-34.9869	3.8646
ligand021	-59.0583	-7.9164	2.4283	-21.3334	3.2189	-19.7092	3.2314
ligand022	-51.5326	-11.9791	2.2663	-25.3546	3.8923	-23.1903	3.8595
ligand023	-56.245	-19.2267	2.5873	-44.49	4.7502	-48.9204	4.6216
ligand024	-74.2541	-22.2749	2.6824	-36.1243	4.1961	-36.7666	3.8751
ligand025	-76.2109	-24.2268	3.0268	-52.8629	5.1084	-57.6948	4.909
ligand026	-60.0403	-19.6773	2.8204	-40.5396	4.8418	-46.2705	4.4913
ligand027	-72.6273	-23.7554	3.9453	-47.6507	4.7456	-48.1006	4.8169
ligand028	-54.8108	-31.1765	3.1572	-47.0695	4.4655	-49.7521	4.0927
ligand029	-48.2129	-20.2823	2.1889	-44.7591	4.4064	-52.6585	4.6045
ligand030	-67.6425	-37.5783	3.3437	-56.7791	4.612	-60.8939	4.2372
ligand031	-63.7372	-21.5195	3.8299	-36.872	5.6083	-40.2315	6.2802

ligand032	-9.74387	-23.9897	2.5202	-42.0301	4.3893	-46.4233	4.4399
ligand033	-75.3808	-14.4987	3.2823	-39.032	4.9488	-51.5427	4.8945
ligand034	-69.1925	-20.4628	2.8476	-36.2602	4.1377	-39.7656	4.4006
ligand035	-73.1411	-16.8609	2.7105	-37	4.1911	-38.7365	3.9561
ligand036	-66.0336	-8.8858	2.1564	-25.3682	4.9787	-38.642	4.0363
ligand037	-71.3334	-28.7706	3.5669	-42.6553	4.5878	-42.6667	4.3997
ligand038	-54.8357	-20.6242	2.2587	-50.8085	4.3085	-53.4322	4.4648
ligand039	-65.9131	-18.3883	2.1867	-40.9721	4.7778	-43.3833	4.8124
ligand040	-57.2143	-16.2965	2.6301	-24.9716	5.7508	-24.1787	5.6239
ligand041	-60.3934	-35.8206	3.9436	-77.4514	7.398	-87.7827	7.6582
ligand042	-59.9097	-11.6054	2.6278	-34.3713	4.5531	-38.603	4.5547
ligand043	-73.8069	-2.5311	1.6674	-9.6624	5.5076	-24.2348	5.0666
ligand044	-65.6142	-13.6548	1.7178	-35.3856	3.883	-35.2906	4.1945
ligand045	-59.6673	-9.1234	2.0882	-31.0296	4.5379	-33.0557	4.3979
ligand046	-55.7169	-30.3773	5.2537	-88.7815	8.5496	-104.797	8.8316
ligand047	-76.953	-20.2114	2.6149	-41.3806	4.4548	-50.4821	4.9993
ligand048	-77.0919	-15.6472	2.527	-42.3695	5.0358	-50.4864	5.0035
ligand049	-59.4491	-18.7754	3.2165	-28.6966	4.133	-28.1829	3.9402
ligand050	-52.8406	-19.7425	2.3584	-40.5261	4.3157	-53.4963	5.0121
ligand051	-64.3707	-10.705	3.992	-35.2871	4.4006	-39.1745	4.4279
ligand052	-61.9059	-14.8723	3.5091	-55.3045	6.5451	-69.6711	7.3227
ligand053	-69.6195	-8.7112	2.543	-32.2735	5.8657	-39.8826	6.772
ligand054	-78.8004	-14.2644	2.6206	-47.7456	5.2248	-48.1268	4.9253
ligand055	-62.1473	-11.3992	2.3829	-37.2918	4.6939	-37.1419	4.6284
ligand056	-73.1321	-15.5851	2.3886	-36.8533	4.1542	-43.4095	4.2231
ligand057	-70.2812	-22.9063	3.3185	-53.3906	5.43	-56.0385	5.2004
ligand058	-58.1838	-17.3435	2.2911	-43.6684	4.6116	-48.9422	4.1966
ligand059	-68.9767	-19.7922	3.8324	-43.4314	4.5395	-44.7877	4.7506
ligand060	-74.7404	-14.0003	2.4522	-34.1953	4.3887	-36.8054	3.798
ligand061	-46.8924	-22.5792	2.3953	-48.4491	4.0838	-52.2444	4.1963
ligand062	-61.2851	-20.2118	2.5732	-44.5713	5.2144	-50.6261	5.6066
ligand063	-71.6755	-20.8762	2.6322	-41.9943	4.534	-41.2071	4.6885
ligand064	-52.7141	-30.5391	2.8484	-52.252	3.656	-57.8125	3.4391
ligand065	-59.1486	-24.3501	3.5398	-39.1417	5.7291	-38.8186	5.5217
ligand066	-71.0388	-13.2596	3.7906	-37.3264	4.4122	-41.2258	4.5741
ligand067	-58.5131	-5.7632	2.6098	-29.977	5.1607	-44.0223	5.1521
ligand068	-60.2162	-4.3243	2.2706	-26.0732	5.9079	-28.3484	7.0692
ligand069	-69.6281	-11.1719	2.3577	-37.3834	4.7784	-39.2087	4.9943
ligand070	-60.7594	-7.0189	2.8506	-42.8846	4.3264	-44.131	4.5353
ligand071	-57.6564	-17.0555	2.7569	-38.4703	3.8786	-39.7595	3.8789

ligand072	-58.1202	-6.7795	3.0462	-23.9241	4.823	-31.4011	4.08
ligand073	-70.1512	-20.9154	3.2075	-43.2743	4.5066	-46.388	4.0642
ligand074	-62.1564	-27.4713	3.2879	-44.6026	4.4604	-45.8852	4.1597
ligand075	-66.6178	-16.0862	2.5156	-23.7524	5.4903	-30.2307	5.3905
ligand076	-73.352	-24.7434	3.4098	-52.6559	5.9898	-52.8304	6.1145
ligand077	-72.1379	-15.4395	3.4933	-34.8188	4.7466	-34.6044	4.3344
ligand078	-71.2988	-20.5448	2.4851	-48.7313	4.5789	-50.3142	4.885
ligand079	-74.7449	-4.2861	1.9927	-18.6345	5.1533	-38.2028	5.2968
ligand080	-69.6266	-20.3876	3.0796	-37.005	4.0648	-40.4194	3.3995
ligand081	-71.8541	-10.155	2.8978	-39.2091	4.2042	-42.7396	4.2751
ligand082	-61.3149	-21.4644	6.0916	-28.7563	8.969	-31.1977	7.3366
ligand083	-61.3935	-22.8342	3.1723	-36.7347	4.2025	-36.799	4.2213
ligand084	-54.1001	-21.7002	2.7777	-38.5935	4.8453	-39.0048	4.9992
ligand085	-63.3705	-29.9426	2.7667	-45.2912	4.1484	-48.5353	4.2302
ligand086	-64.0545	-24.2156	2.9976	-30.346	5.1366	-30.1476	5.0653
ligand087	-74.989	-26.7246	5.8932	-55.9766	5.575	-57.302	5.8431
ligand088	-58.0258	-48.8511	15.7413	25.1776	16.886	33.1098	16.4534
ligand089	-59.8857	-15.0144	3.6843	-37.2024	3.9946	-37.3566	3.6769
ligand090	-53.3482	-15.0399	1.5427	-26.8562	2.9895	-29.2906	2.8895
ligand091	-62.251	-18.6565	3.294	-34.2774	4.5988	-36.9122	4.569
ligand092	-61.1433	-13.7639	1.5084	-37.8393	4.695	-53.5033	5.0967
ligand093	-72.4006	-16.2236	3.6016	-30.8671	4.8176	-35.8352	4.7002
ligand094	-74.2915	-16.7005	2.4095	-35.0993	4.3917	-37.2952	3.8732
ligand095	-67.421	-12.4342	2.634	-32.7229	5.0119	-33.2093	5.059
ligand096	-66.6877	-7.0567	2.4609	-31.0692	4.9452	-40.1109	4.8703
ligand097	-67.338	-12.2543	2.694	-32.696	4.1691	-39.4649	4.3771
ligand098	-66.3009	-13.6973	2.8818	-36.9594	5.4517	-39.4603	5.5475
ligand099	-73.9137	-28.639	5.9047	-52.8812	5.9472	-54.1526	6.2384
ligand100	-69.3176	-29.2891	3.1027	-66.192	7.1816	-67.6791	7.5511
ligand101	-63.5958	-29.1653	3.5487	-68.0883	7.2894	-71.4269	7.5889
ligand102	-63.7427	-11.2603	2.5027	-22.7976	4.1488	-29.3912	4.6508
ligand103	-69.1476	-28.624	2.9599	-47.9809	3.8228	-48.5786	3.795
ligand104	-70.3421	-11.8251	3.5317	-35.1838	5.8108	-38.7869	4.9355
ligand105	-66.0978	-24.033	3.1064	-36.4858	4.6676	-34.7255	4.668
ligand106	-52.0467	-14.1167	2.0194	-25.101	4.9076	-25.6999	5.0727
ligand107	-69.3275	-18.4252	3.2323	-36.0633	3.9282	-37.7715	3.9803
ligand108	-70.5258	-23.6985	2.6797	-37.2627	3.5189	-37.2023	3.5136
ligand109	-60.5565	-21.5477	3.134	-38.6805	5.4906	-39.3499	5.6219
ligand110	-63.3425	-20.2041	1.9895	-30.324	3.344	-31.9958	3.3254
ligand111	-67.6784	-29.906	2.286	-47.0539	3.8592	-47.8452	3.8341

ligand112	-60.1187	-10.2163	2.9762	-36.9273	5.4028	-42.2553	5.6536
ligand113	-62.8853	-19.5689	2.52	-30.2508	3.1567	-31.9139	3.035
ligand114	-71.6795	-28.6192	3.6154	-56.0356	6.4963	-56.218	6.3939
ligand115	-59.7622	-6.8124	2.6464	-22.5157	4.2368	-25.3819	3.861
ligand116	-63.8882	-33.9738	7.0351	-79.3015	10.2641	-78.4786	10.1062
ligand117	-60.7223	-9.0439	3.2554	-34.0191	5.1999	-36.3873	5.4828
ligand118	-69.4364	-18.2295	3.5843	-38.292	4.4111	-38.1003	4.2936
ligand119	-56.5245	-7.8731	2.3415	-32.1114	7.4517	-33.8037	8.3832
ligand120	-67.995	-12.768	3.4241	-44.6483	8.7866	-44.1768	9.8499
ligand121	-64.2921	-8.2804	1.9319	-39.1227	4.6759	-51.5887	6.6691
ligand122	-76.5576	-22.8826	3.7694	-44.8286	5.6478	-45.1711	5.4875
ligand123	-69.1113	-23.1203	3.16	-48.9916	3.9519	-49.4111	4.0323
ligand124	-65.9615	-18.8948	2.857	-39.3575	5.6033	-40.05	5.9657
ligand125	-76.5666	-24.5203	4.1163	-51.9835	5.3993	-52.4972	5.3493
ligand126	-56.5204	-7.2777	2.6359	-56.5599	9.0302	-65.2121	8.8105
ligand127	-64.728	-31.2445	5.9774	-66.4541	7.6364	-65.2744	7.7059
ligand128	-67.2841	-16.7413	3.0672	-52.3318	4.2516	-55.8131	4.5991
ligand129	-67.1943	-19.5428	2.9915	-46.9302	4.5545	-45.9177	4.6874
ligand130	-66.9712	-14.2097	4.0321	-43.7212	6.3803	-44.1936	6.0234
ligand131	-60.3751	-9.1155	3.1127	-40.8943	5.6906	-45.6904	5.9879
ligand132	-64.461	-5.0193	2.637	-31.3036	9.298	-47.2193	6.4498
ligand133	-63.9621	-26.2048	2.5569	-56.3991	6.8931	-57.6017	6.7005
ligand134	-67.9829	-24.7463	4.4131	-38.5611	3.6222	-40.0989	3.5927
ligand135	-64.9035	-19.7942	4.6544	-50.3479	5.412	-56.9659	6.6353
ligand136	-57.0045	-13.0777	2.6022	-36.346	4.1241	-37.3293	4.0574
ligand137	-65.1934	-18.6995	2.6646	-42.1544	5.7936	-43.9162	6.3602
ligand138	-56.8342	-17.0335	2.742	-44.8146	5.1089	-45.8231	4.6716
ligand139	-76.4889	-12.7325	2.2731	-68.5119	6.6613	-100.827	7.4467
ligand140	-56.9201	-20.371	3.4693	-60.9536	7.1076	-69.5429	6.5692
ligand141	-49	-12.1068	4.1703	-26.6619	6.8929	-31.8426	7.318
ligand142	-57.0472	-37.0566	5.0114	-65.1737	5.2875	-63.8762	5.6877
ligand143	-46.9285	N.C.	N.C.	N.C.	N.C.	N.C.	N.C.
ligand144	-48.6334	-10.9502	2.5963	-21.6979	2.7546	-21.2284	2.8502
ligand145	-51.9493	-7.1205	2.239	-15.9609	3.5852	-16.0613	3.4849
ligand146	-10.2191	-19.6962	3.3844	-36.2549	4.9628	-40.54	4.2897
ligand147	-14.5807	-28.2915	3.5154	-44.9939	4.1649	-44.8962	4.3462
ligand148	-53.3213	-22.5554	4.2638	-68.0239	7.1906	-72.9698	6.8743
ligand149	-58.2036	-36.8406	5.0799	-55.5185	6.3863	-54.8822	6.2557
ligand150	-72.4411	-19.3142	6.1895	-60.9761	7.9719	-63.725	7.7509
ligand151	-49.4343	-9.1305	3.3418	-16.6417	5.0445	-16.6392	4.9539

ligand152	-64.9338	-29.6338	3.4272	-26.9631	5.253	-26.4733	5.262
ligand153	-57.8217	-9.087	2.2842	-15.6036	4.2127	-15.6845	4.2073
ligand154	-56.66	-6.6554	1.6022	-21.7266	4.6302	-22.2689	4.1131
ligand155	-61.1741	-30.2082	2.7205	-45.5367	4.088	-46.5215	3.9062
ligand156	-62.4529	-13.6365	2.0918	-36.1692	4.9332	-44.4106	4.3644
ligand157	-59.2506	-10.3745	6.459	-9.9617	8.0792	-12.023	5.81
ligand158	-56.477	-15.5515	2.2051	-33.4026	3.3052	-34.6588	3.0911
ligand159	-60.2864	-11.4762	2.0944	-24.1071	3.9311	-26.9701	3.2749
ligand160	-55.9162	-15.7798	3.2066	-21.3962	4.546	-20.3967	4.5035
ligand161	-69.1778	-17.6637	3.4461	-32.5074	4.2444	-32.719	3.9842
ligand162	-62.8584	-7.5076	2.6423	-30.8392	4.2045	-31.4079	4.0391
ligand163	-74.6863	-20.0105	3.5525	-35.9293	3.9381	-39.5772	3.5231
ligand164	-68.5198	-10.6081	2.6926	-31.8494	5.2196	-36.4149	4.2312
ligand165	-69.3835	-7.7873	1.4443	-22.7906	4.1279	-26.0234	4.2527
ligand166	-79.9213	-23.3806	3.2498	-43.5956	4.6661	-44.0722	4.0675
ligand167	-63.4987	-12.3467	2.5337	-24.9847	3.1101	-26.0995	2.7733
ligand168	-61.161	-11.7875	3.1649	-27.6347	4.0899	-32.9248	3.3656
ligand169	-71.6114	-8.4045	1.6244	-25.2788	3.4758	-29.3884	3.4024
ligand170	-73.8066	-21.8055	2.7616	-39.7128	5.4858	-41.2163	5.374
ligand171	-66.1157	-12.9693	2.4753	-31.5045	3.836	-32.7537	3.586
ligand172	-73.2997	-27.6602	3.0979	-42.9055	5.0801	-42.8211	4.8783
ligand173	-59.3466	-32.9432	5.4124	-41.5379	4.1534	-41.3324	4.1623
ligand174	-59.0884	-15.8529	4.6257	-58.2201	6.8654	-67.3315	6.122
ligand175	-58.0832	-14.1565	8.273	-53.2265	7.294	-57.8079	7.0707
ligand176	-56.7691	-5.7421	3.2023	-24.9456	4.6225	-30.3613	3.2353
ligand177	-64.2864	-11.9484	3.1685	-42.1766	4.4007	-45.9982	4.2503
ligand178	-56.686	-15.017	2.4694	-41.9724	7.2444	-44.5757	7.3621
ligand179	-70.3805	-86.4797	15.1791	20.624	20.2479	12.3451	20.9188
ligand180	-63.7357	-24.8028	3.6769	-63.4844	6.6617	-64.6399	6.7333
ligand181	-65.6897	-27.7353	5.0037	-50.4111	4.9166	-50.0701	5.0057
ligand182	-70.7817	-26.6558	3.622	-53.2506	4.6474	-55.3196	4.3552
ligand183	-52.1654	-15.9074	2.18	-26.187	4.5411	-25.3141	4.461
ligand184	-73.2702	-14.2175	2.9064	-32.0476	5.1378	-38.4055	5.0699
ligand185	-66.5299	-9.9174	1.7475	-14.8491	3.97	-18.252	3.8405
ligand186	-65.9178	-17.4811	3.2736	-32.2807	3.8131	-32.0233	3.8403
ligand187	-67.5913	-17.2943	2.6486	-38.0756	4.2137	-43.3419	4.4929
ligand188	-62.4171	-17.9929	2.7701	-41.2233	4.6679	-40.995	4.3511
ligand189	-64.8143	-27.597	2.6044	-46.3628	4.6094	-48.147	4.6167
ligand190	-57.8174	-17.1764	2.9616	-31.9965	4.3384	-32.2365	4.3962
ligand191	-79.6909	-45.3854	4.7834	-73.2374	6.4399	-72.6418	6.3787

ligand192	-76.9184	-26.4625	3.9667	-68.049	8.4749	-68.4567	8.5604
ligand193	-63.7175	-15.7926	2.8261	-21.0422	4.3536	-20.0009	4.3312
ligand194	-69.4305	-10.6016	3.2403	-22.0617	4.8938	-23.2801	4.5343
ligand195	-52.4214	-17.8623	2.4711	-31.9132	4.4115	-46.9003	4.298
ligand196	-67.0166	-16.3556	2.9245	-33.1318	4.0738	-33.3552	4.1374
ligand197	-63.8368	-12.3658	2.1932	-27.6849	4.1685	-37.852	4.2471
ligand198	-70.1429	-9.2924	1.7511	-25.0743	5.3005	-30.303	4.7429
ligand199	-67.911	-14.0978	2.7026	-29.9586	4.611	-31.2401	4.4067
ligand200	-71.2903	-5.2133	1.6199	-23.6228	4.8858	-34.881	4.5072
ligand201	-66.3658	-12.6479	2.6031	-33.0347	4.7207	-34.2621	4.2693
ligand202	-78.1063	-0.2868	1.3586	-21.583	7.4511	-35.5836	10.7475
ligand203	-72.5511	-21.4578	4.7732	-37.0677	4.2853	-37.9755	4.0237
ligand204	-78.2288	-15.115	2.2381	-34.6992	4.1943	-37.5877	3.6747
ligand205	-65.8803	-30.2352	3.7222	-48.5459	4.2904	-48.5227	4.3093
ligand206	-52.7795	-11.3291	2.9706	-45.3391	4.4052	-48.8931	4.7841
ligand207	-61.1463	-30.7249	3.3473	-48.5209	4.6675	-48.3149	4.6122
ligand208	-64.8274	-8.3539	2.9144	-32.9146	5.3727	-41.952	5.0085
ligand209	-59.6896	-11.9288	3.0751	-30.722	4.6888	-31.8971	4.4067
ligand210	-64.9258	-16.0349	2.7377	-34.2846	3.7846	-34.9809	3.3348
ligand211	-78.2191	-33.5862	3.8649	-55.8072	5.6266	-59.6873	5.6433
ligand212	-63.3363	-20.4258	2.5339	-31.3596	3.4496	-31.4197	3.3254
ligand213	-67.658	-17.4221	4.4437	-34.8158	5.4448	-39.5866	5.0956
ligand214	-67.8352	-27.0786	3.1962	-43.7562	3.7698	-43.9695	3.68
ligand215	-71.8944	-14.9509	2.0356	-29.961	4.3295	-35.8355	4.2017
ligand216	-71.0029	-15.2368	2.6532	-47.3928	4.9339	-46.5481	5.2094
ligand217	-49.0484	-24.8872	2.1946	-49.5371	4.5073	-54.0958	4.6613
ligand218	-73.4337	-18.8388	4.383	-58.5278	7.086	-61.4926	7.0639
ligand219	-76.0616	-34.4062	7.2278	-62.6665	8.6344	-64.0896	8.4834
ligand220	-59.8682	-13.2088	2.4606	-32.7698	3.8099	-33.7958	3.8603
ligand221	-50.9973	-10.2595	2.7792	-23.7119	4.0156	-23.9417	3.604
ligand222	-66.2658	-19.1779	3.1438	-29.7821	3.977	-29.3528	3.937
ligand223	-66.3913	-18.3104	2.7729	-35.3543	4.5662	-38.5011	4.3178
ligand224	-53.1111	-27.3558	3.79	-36.6357	4.2894	-35.9641	4.2303
ligand225	-68.1715	-9.3207	3.4644	-38.3778	5.6026	-44.1553	5.1397
ligand226	-73.7332	-22.1112	6.0083	-53.4122	6.8822	-54.4395	6.8402
ligand227	-69.1872	-38.8824	4.2511	-50.6292	5.1557	-48.9817	5.057
ligand228	-67.6645	-27.3932	3.4417	-50.3882	5.2982	-48.9396	5.5574
ligand229	-45.2792	-5.7987	1.2075	-13.1944	2.376	-15.4716	2.3668
ligand230	-47.4782	-8.2718	1.5317	-17.4735	2.5056	-16.7451	2.5322
ligand231	-56.1834	-29.7841	7.2117	-50.9637	5.9748	-49.2063	5.9167

ligand232	-62.0024	-12.2536	2.9517	-19.3013	6.2304	-26.2313	5.7779
ligand233	-41.6479	-2.4398	3.1419	-7.8506	4.0456	-10.8528	3.5292
ligand234	-61.9861	-7.3196	3.2642	-27.1473	5.1984	-30.0366	4.8388
ligand235	-67.1438	-24.2393	3.0183	-30.5847	4.3049	-28.6157	4.2494
ligand236	-55.0426	-18.3021	2.058	-28.452	2.4795	-28.1301	2.4334
ligand237	-50.6039	-6.3402	2.7242	-18.8261	4.6366	-22.9171	3.5884
ligand238	-56.5122	-19.5139	2.1545	-31.029	4.4089	-30.9747	4.5899
ligand239	-61.85	-20.3771	3.5985	-28.1504	5.2481	-28.263	4.9584
ligand240	-67.8413	-17.8098	2.6778	-33.8979	3.5442	-33.6432	3.1726
ligand241	-60.8185	-9.2979	3.1233	-67.3453	10.0598	-72.534	10.6413
ligand242	-58.4355	-7.3371	3.6354	-58.3213	9.3626	-67.7011	15.7357
ligand243	-73.0394	-21.7289	6.3419	-62.2075	7.3312	-66.6594	7.2978
ligand244	-65.4135	-25.2303	4.6088	-40.7453	4.2898	-41.5469	4.0826
ligand245	-64.753	-11.5545	1.9734	-30.1803	3.581	-36.0726	3.715
ligand246	-70.3561	-25.6507	3.0836	-37.3777	3.771	-35.2743	3.7071
ligand247	-66.7058	-13.0101	3.1411	-35.623	3.9951	-37.9715	4.0286
ligand248	37.5774	-11.4307	2.3023	-25.132	4.8342	-40.1873	4.3243
ligand249	-52.7815	-21.5827	4.4699	-62.5546	7.0487	-63.8683	7.2617
ligand250	-61.6842	-20.5136	3.2387	-95.5551	12.4896	-101.121	13.1233
ligand251	-65.9348	103.8008	12.635	-72.8589	11.9871	-73.9381	12.4334
ligand252	-53.2369	-6.0398	3.0183	-15.7896	3.7859	-15.9901	3.8127
ligand253	-57.5046	-9.9986	2.7201	-26.1658	5.0161	-24.8348	5.0664
ligand254	-71.7336	-19.4926	2.8564	-29.7375	4.3167	-30.9916	4.5644
ligand255	-47.5903	-10.1168	1.8825	-21.2272	2.6613	-21.2563	2.6412
ligand256	-65.5988	-24.6787	2.1521	-48.6826	4.5285	-50.1126	4.3044
ligand257	-67.3966	-9.959	2.6647	-35.4216	4.4822	-38.8304	4.4917
ligand258	-64.6662	-5.2236	3.026	-22.9295	5.2196	-27.3734	4.196
ligand259	-71.2242	-9.8223	4.359	-28.1771	4.9839	-30.2954	4.5746
ligand260	-67.5952	-17.4934	2.458	-33.8081	4.3059	-33.3583	4.0784
ligand261	-56.7369	-22.4986	2.4086	-36.714	3.4549	-35.8049	3.3412
ligand262	-62.3209	-9.9208	2.3727	-33.6563	3.9156	-36.1939	3.8142
ligand263	-74.6665	-17.6997	3.9839	-30.3508	5.9179	-31.6088	5.93
ligand264	-51.1674	-13.022	1.7804	-24.1635	2.6538	-23.7665	2.7844
ligand265	-69.216	-8.4162	2.1834	-25.1866	4.6197	-33.4993	4.897
ligand266	-62.0138	-22.1056	2.6527	-36.919	3.7909	-37.6361	3.7453
ligand267	-60.5265	-8.3304	2.451	-22.9541	4.5811	-26.0551	4.756
ligand268	-65.5178	-11.5907	2.3808	-29.4463	3.6745	-30.2401	3.8577
ligand269	-67.7307	-9.3156	2.0295	-27.3747	4.6634	-28.1195	4.6107
ligand270	-64.0025	-29.561	3.4247	-44.1158	3.9226	-43.4237	3.9538
ligand271	-66.2943	-14.0015	2.5419	-38.0928	5.0159	-41.1508	5.1641

ligand272	-72.9021	-20.085	4.2255	-43.0557	6.6933	-49.0623	4.9652
ligand273	-75.262	-24.6857	3.3843	-48.3715	4.884	-50.6905	4.3362
ligand274	-54.7765	-20.1445	3.0196	-36.5747	5.4751	-37.2306	5.6564
ligand275	-51.0784	0.0187	0.0924	-4.4153	3.3157	-11.5148	5.1474
ligand276	-61.6179	-27.0928	4.8631	-44.5618	5.5544	-44.1019	5.2747
ligand277	-58.0141	-16.9989	1.9488	-28.8782	2.9664	-28.7539	2.922
ligand278	-65.2549	-24.357	2.2624	-39.8582	4.0088	-39.6743	4.0033
ligand279	-64.986	-17.4562	1.8438	-39.156	3.6617	-39.9322	3.5548
ligand280	-71.5723	-20.7795	2.406	-43.7535	3.8199	-46.5001	3.8013
ligand281	-63.8205	-8.2884	3.8041	-33.0761	6.0983	-37.9731	5.6867
ligand282	-66.904	-16.7402	3.892	-38.7084	4.5678	-40.2304	4.2726
ligand283	-66.9794	-17.3479	3.0707	-35.3116	3.6996	-35.8087	3.7573
ligand284	-65.5285	-16.1978	2.9965	-39.6108	5.0179	-41.4859	4.6822
ligand285	-59.6148	-19.4749	3.302	-30.2417	4.1246	-28.8272	4.2594
ligand286	-66.6118	-20.7298	2.771	-35.0152	5.0942	-36.8665	5.0523
ligand287	-58.3389	-7.0209	2.3827	-3.4698	5.1646	-4.0242	5.1532
ligand288	-58.1056	-11.5173	3.021	-26.3345	5.2262	-25.0248	5.296
ligand289	-52.1125	-10.359	3.1771	-25.1331	4.1786	-24.6574	4.2377
ligand290	-75.5658	-11.5096	2.388	-31.9165	3.6324	-36.2624	3.449
ligand291	-65.8571	-8.7581	2.5322	-17.2772	5.1653	-18.6081	5.2783
ligand292	-61.9197	-8.6169	2.2563	-24.6929	3.8965	-27.2122	3.6592
ligand293	-69.4092	-8.2523	1.8213	-34.0419	5.1961	-37.4572	4.9586
ligand294	-63.8968	-29.1151	3.571	-43.1998	5.1687	-42.7914	5.1584
ligand295	-60.1722	-15.197	3.7498	-41.6542	4.3942	-40.9657	4.3139
ligand296	-62.8038	-28.2386	2.4947	-51.3663	4.3891	-50.4928	4.4585
ligand297	-64.7659	-17.9824	2.7595	-31.0007	3.8475	-30.5473	3.8476
ligand298	-60.47	-10.5049	3.1635	-25.787	4.0338	-25.2671	4.2788
ligand299	-69.0725	-5.5532	2.8948	-22.8137	5.8107	-31.4656	5.154
ligand300	-76.7898	-29.9075	4.053	-53.2241	4.7935	-53.2526	4.6637
ligand301	-60.5567	-14.2285	3.7859	-3.961	6.694	-7.8738	7.2735
ligand302	-70.1541	-16.5722	3.4944	-41.2113	6.9202	-45.2286	6.9593
ligand303	-66.2748	-18.4888	4.1093	-28.3615	6.281	-28.9153	6.5844
ligand304	-69.4435	-33.1138	7.1626	-57.4648	6.891	-56.8426	6.8411
ligand305	-64.9053	-11.8981	1.9806	-31.0873	6.4082	-34.863	7.0275
ligand306	-60.6495	-13.9946	1.8012	-31.6916	4.0583	-32.3029	3.9266
ligand307	-54.2871	-6.8007	3.0336	-31.2401	4.0738	-30.5336	4.3109
ligand308	-55.8451	-20.5649	2.5596	-37.1233	3.4419	-36.2382	3.2824
ligand309	-68.0855	-24.9277	2.8346	-55.5291	5.9138	-54.1895	5.7754
ligand310	-75.0873	-10.4395	1.9972	-45.2335	4.5473	-50.2754	4.4444
ligand311	-55.789	-4.8985	2.0201	-19.6326	4.7997	-30.7768	4.4561

ligand312	-57.2852	-25.35	3.3441	-37.3543	3.5457	-37.3286	3.577
ligand313	-58.7577	-8.9802	3.374	-25.7246	4.4647	-27.7591	4.2381
ligand314	-54.0899	-13.9806	2.5111	-26.793	3.7423	-27.4324	3.6766
ligand315	-51.7472	-11.7439	2.3243	-28.5639	3.83	-28.6258	3.8308
ligand316	-59.2056	-18.6488	2.4334	-44.104	6.3415	-45.8105	5.9331
ligand317	-56.3668	-5.9753	1.6234	-26.1744	3.9437	-28.5372	4.3003
ligand318	-64.9873	-10.8563	3.2598	-43.6715	6.0648	-49.9985	6.8441
ligand319	-74.5517	-16.2645	3.5203	-40.5907	6.2043	-43.6966	6.3042
ligand320	-55.999	-4.207	3.2708	-29.0319	5.5408	-38.2602	3.6812
ligand321	-63.998	-13.6483	2.3139	-34.0934	4.0067	-32.4125	3.8979
ligand322	-58.9373	-11.3114	2.889	-35.0594	6.546	-38.181	6.277
ligand323	-58.7581	-10.9241	3.8833	-30.1117	6.0082	-33.6206	4.8833
ligand324	-56.1102	-5.6907	4.2421	-52.1031	13.5464	-71.3331	8.3327
ligand325	-55.7229	-21.6087	4.1097	-44.9505	8.1013	-41.3592	8.4889
ligand326	-66.8907	-10.2557	2.0974	-39.6747	4.0017	-41.1038	4.4695
ligand327	-64.4149	-4.5249	2.4903	-28.6113	4.7761	-36.0547	5.8204
ligand328	-54.6613	-15.7191	3.2178	-48.8449	7.0187	-48.4296	7.2103
ligand329	-63.8881	-2.6829	2.0181	-35.5962	6.1444	-35.5358	6.4474
ligand330	-54.2257	-3.1545	2.7323	-20.9827	3.2839	-20.7503	3.2375
ligand331	-63.4696	-4.7341	2.183	-36.773	4.665	-39.4363	4.2778
ligand332	-68.6829	-27.873	3.8652	-58.8927	6.6633	-65.0462	6.1425
ligand333	-65.9698	-24.7386	3.0229	-42.1774	3.688	-43.5623	3.8079
ligand334	-58.8209	-22.1502	4.9123	-36.1556	6.9944	-35.9968	6.9122
ligand335	-60.5931	-13.7231	3.3026	-28.4127	3.5123	-28.313	3.5031
ligand336	-61.9192	-11.622	2.5312	-32.0595	4.4729	-31.0893	4.4031
ligand337	-60.8766	-9.6511	2.3327	-27.5923	4.48	-31.7476	4.4467
ligand338	-51.2818	-8.553	2.2634	-17.3706	3.636	-16.6341	3.627
ligand339	-52.7848	-16.8796	2.6049	-29.9869	3.5629	-30.0292	3.5758
ligand340	-64.2974	-14.3361	3.2581	-27.1162	5.5443	-27.1263	5.9439
ligand341	-60.2074	-16.782	10.5369	-75.4831	9.5961	-81.8063	9.4241
ligand342	-53.9143	-47.4842	4.7652	-39.483	8.3053	-37.3081	8.5268
ligand343	-76.3217	-23.0453	6.6743	-68.2929	8.5494	-69.2371	8.4281
ligand344	-64.5604	-50.8519	4.8896	-65.4924	5.9004	-66.5776	5.8368
ligand345	-68.3236	-25.9081	2.6403	-56.2281	5.2856	-56.9264	5.3079
ligand346	-58.0748	-12.5035	4.8922	-29.3511	5.216	-29.6315	4.4486
ligand347	-52.2417	-4.3327	1.8592	-35.9111	5.2917	-39.8118	5.5298
ligand348	-53.7417	-23.4736	4.3009	-98.5392	9.7979	-99.7449	10.0455
ligand349	-63.0612	-16.6673	3.2565	-38.6696	4.5833	-38.8461	4.5312
ligand350	-66.3393	-14.9111	2.2452	-30.2533	5.0285	-31.1231	4.8068
ligand351	-65.35	-11.1844	2.7691	-43.1975	4.8598	-42.7717	4.8046

ligand352	-70.6389	-26.8648	2.7911	-55.3605	5.4233	-56.2864	5.5479
ligand353	-65.4946	-11.6262	2.0039	-44.1758	4.7991	-45.8182	4.5616
ligand354	-71.043	-13.067	5.4443	-44.849	5.8011	-46.3267	5.2757
ligand355	-63.387	-39.2264	5.4962	-63.7613	6.7477	-63.1151	6.7854
ligand356	-55.4503	-1.4424	1.4309	-29.0961	3.9417	-37.2001	4.6698
ligand357	-55.321	-5.3487	2.7569	-22.8581	4.3736	-23.496	4.543
ligand358	-62.2778	-6.5687	3.9763	-25.3129	4.9882	-28.4629	6.7403
ligand359	-57.2408	-15.8507	2.8168	-32.4959	4.7139	-31.2916	4.6415
ligand360	-54.6363	-1.208	2.4672	-19.2632	5.638	-29.3868	8.8432
ligand361	-73.0586	-19.6304	4.5868	-67.1423	7.3676	-69.7319	7.2493
ligand362	-62.1403	-16.1288	3.9375	-27.5282	4.5035	-30.6145	4.0558
ligand363	-64.4926	-24.5796	3.9727	-43.8173	5.8933	-42.7916	5.8631
ligand364	-63.6827	-37.6927	12.4792	-54.4774	12.6187	-56.3153	13.157
ligand365	-61.2926	-6.4007	2.1634	-22.7307	5.0726	-24.597	5.8327
ligand366	-57.0564	-4.3603	2.3966	-22.7523	4.113	-22.2965	4.2242
ligand367	-73.3334	-3.2632	2.9369	-30.8975	6.462	-48.4011	5.5024
ligand368	-63.8941	-6.1798	2.1596	-37.1448	5.0261	-41.9042	5.1627
ligand369	-65.2812	-11.5193	2.6634	-36.578	4.3725	-42.7021	4.9441
ligand370	-64.5134	-20.9899	3.189	-57.9269	5.2514	-56.8728	5.5752
ligand371	-63.1289	-15.6845	3.1631	-35.667	4.2382	-34.9855	4.1412
ligand372	-63.501	0.2641	0.1225	-13.0286	5.3212	-37.845	6.6581
ligand373	-60.1371	-13.6841	2.7507	-33.4587	4.6332	-33.3745	4.7589
ligand374	-52.5008	-13.7056	2.1767	-22.4217	2.5279	-21.6694	2.5895
ligand375	-57.0217	-11.1163	2.4217	-25.5047	3.4339	-26.2535	3.1887
ligand376	-56.4762	-15.1563	2.1015	-22.1828	3.5877	-22.2335	3.7891
ligand377	-48.7469	-19.8194	1.996	-31.1614	3.9669	-31.793	4.0638
ligand378	-50.7144	-14.8588	5.0061	-64.3404	7.1415	-68.0877	7.7309
ligand379	-56.3775	-21.5663	3.9882	-127.673	14.7756	-136.256	15.3618
ligand380	-70.5359	-21.7666	4.4928	-59.5642	7.4197	-60.727	7.6887
ligand381	-65.0127	-18.347	3.0193	-56.79	5.9486	-62.2419	6.2562
ligand382	-73.218	-26.4648	2.8313	-61.999	6.2599	-62.3921	6.3662
ligand383	-76.5004	-44.6952	4.5893	-66.2489	6.4033	-66.589	6.2577
ligand384	-72.0781	-17.2283	2.2684	-60.6778	6.9613	-65.7791	6.9369
ligand385	-60.7493	-28.588	4.0611	-45.3032	5.7942	-44.6519	5.775
ligand386	-53.5548	-12.6309	2.7328	-50.0792	5.874	-50.5866	6.108
ligand387	-79.4375	-45.6572	5.1625	-97.2462	8.8865	-102.84	10.0944
ligand388	-79.1045	-45.2139	4.922	-94.6382	7.6346	-99.768	8.2456
ligand389	-85.3136	-42.2538	6.9188	-107.721	7.7293	-115.501	8.5791
ligand390	-78.0745	-60.0788	4.1045	-102.899	8.1658	-109.266	8.7324
ligand391	-74.788	-10.3502	3.7951	-46.4623	6.7805	-49.1357	7.0496

ligand392	-80.4445	-27.6107	3.2601	-64.9937	6.7385	-67.1763	7.3919
ligand393	-75.0061	-34.0457	4.7151	-68.9005	6.1157	-69.1173	6.0322
ligand394	-65.6208	-24.1661	4.9994	-60.0259	6.6038	-61.3425	6.4838
ligand395	-71.7042	-14.2907	2.5667	-47.232	6.4454	-50.979	6.4414
ligand396	-60.8399	-10.9734	3.7675	-46.6585	6.9496	-48.2716	6.4131
ligand397	-63.257	-26.6977	2.8753	-52.5873	5.3744	-52.2615	5.5172
ligand398	-55.7034	-20.5095	2.1643	-39.5358	4.7792	-40.7177	4.9133
ligand399	-62.4151	-63.7323	6.5579	-78.4409	8.7213	-80.2673	9.1691

a Parametrization did not converge

Table S48. Rac1 VS docking scores and Nwat-MMGBSA relative binding energies (kcal/mol) computed with Nwat = 0, 30, 60 and 100 (first repetition).

		Nwat=0		Nwat=30		Nwat=60		Nwat=100	
ligand	docking score	ΔG	St.Dev.	ΔG	St.Dev.	ΔG	St.Dev.	ΔG	St.Dev.
ligand001	-70.9032	-16.7861	2.159	-35.3121	4.5384	-51.025	5.4582	-52.8921	5.2351
ligand002	-68.6085	-22.798	2.4288	-33.3837	5.4176	-49.691	5.7748	-54.3782	5.8337
ligand003	-73.6353	-19.5255	3.4303	-30.5643	5.6973	-44.7675	5.4477	-46.6096	4.8032
ligand004	-66.8974	-20.6309	2.381	-32.1237	4.8492	-47.4428	5.4059	-49.0349	5.2348
ligand005	-74.8985	-7.9133	2.1732	-12.2693	6.1418	-33.5268	6.0176	-42.9604	6.1081
ligand006	-82.2695	-14.9868	2.9528	-36.0706	6.7108	-60.696	6.4907	-66.7269	5.8848
ligand007	-85.2919	-16.7364	2.7997	-27.2188	4.9824	-52.884	5.6123	-62.9868	6.046
ligand008	-76.1078	-12.9972	2.9031	-32.0255	7.0734	-51.3068	6.9965	-56.2674	6.574
ligand009	-74.341	-23.1196	2.691	-51.201	5.6327	-56.6251	5.4816	-55.3677	5.4524
ligand010	-59.8709	-19.7235	1.9159	-34.6138	4.0621	-45.6112	3.9942	-47.3216	3.7531
ligand011	-78.5888	-18.8241	3.0671	-42.4767	6.0673	-50.4495	5.508	-50.0837	5.3341
ligand012	-79.9404	-15.1507	2.228	-32.8098	5.6605	-50.917	5.7644	-52.4287	5.584
ligand013	-77.1692	-23.904	2.6454	-49.4778	6.0947	-55.6905	6.4294	-53.7615	6.1472
ligand014	-75.4356	-15.2339	2.5889	-28.4024	5.4652	-47.6734	5.8429	-51.6723	5.7643
ligand015	-86.8566	-8.8186	4.1499	-30.4238	12.1556	-51.7679	8.4551	-56.4969	6.3966
ligand016	-80.5585	-24.3845	4.7753	-40.7603	6.0752	-54.808	5.8148	-57.9275	6.1772
ligand017	-80.3863	-16.813	3.0387	-55.0108	6.2366	-70.0615	6.0875	-70.0898	6.1684
ligand018	-82.3102	-18.2781	3.2035	-54.4532	5.733	-70.4783	6.425	-70.1029	6.4221
ligand019	-78.1472	-13.5535	2.6862	-29.7531	5.3059	-44.8723	5.398	-48.0636	5.2732
ligand020	-77.1408	-25.8711	3.835	-47.7556	5.4276	-60.6193	5.6859	-62.4492	5.5559
ligand021	-77.5195	-5.6319	2.2384	-7.3174	7.444	-38.3688	6.5538	-50.4814	6.6124
ligand022	-78.1927	-17.7837	3.0537	-35.4869	5.9709	-49.524	5.8745	-52.1914	6.0961
ligand023	-76.952	-10.1783	2.8487	-25.6111	5.7879	-45.9575	5.9838	-49.9963	5.823
ligand024	-71.5927	-20.9015	2.817	-44.1086	5.1775	-54.2428	4.8606	-53.4835	4.7177
ligand025	-58.7667	-18.8484	2.1903	-29.5764	5.8897	-50.0663	6.269	-54.4338	5.9496
ligand026	-71.724	-4.6164	2.4766	-35.9849	6.1422	-57.2672	5.1532	-61.1041	5.2195
ligand027	-78.271	-18.6383	4.4827	-36.4723	8.5713	-47.0774	6.8757	-47.8888	6.776
ligand028	-77.252	-25.1811	3.2861	-48.2018	6.2145	-57.3874	5.9733	-57.6143	5.8108
ligand029	-79.4969	-10.7675	2.4121	-17.5849	6.6356	-44.6296	6.2954	-52.4798	5.3508
ligand030	-59.9182	-7.2966	2.0333	-36.0767	4.3536	-46.9486	4.9863	-47.2257	4.9751
ligand031	-68.2406	-13.2147	2.3692	-25.2432	4.1484	-40.0907	4.6858	-42.3526	4.5082
ligand032	-78.313	-13.1252	2.448	-27.9275	5.5843	-55.5495	6.21	-65.5317	6.4648
ligand033	-84.2444	-16.3955	3.0921	-26.6268	5.6403	-56.8202	6.8189	-69.9251	7.5908
ligand034	-76.2475	-14.8664	5.4206	-33.6515	8.8151	-47.5249	5.8298	-49.0519	6.0655
ligand035	-74.8863	-18.4977	2.0893	-50.1061	5.593	-58.4376	5.4821	-58.0465	5.3094
ligand036	-67.131	-19.5766	3.3175	-33.9753	7.1526	-59.6972	7.3355	-69.7869	7.2186

ligand037	-80.4996	-12.3742	3.0443	-25.6271	5.0507	-53.1629	6.1402	-63.7282	6.8381
ligand038	-86.0783	-23.5176	2.32	-33.7419	4.992	-55.6932	5.4909	-62.6186	5.441
ligand039	-71.1128	-23.4675	2.8604	-31.545	5.3561	-55.066	6.227	-63.1345	5.8452
ligand040	-72.6537	-16.755	2.5895	-42.6709	5.0516	-49.7677	5.1883	-48.6558	5.0673
ligand041	-73.2428	-5.1491	2.8288	-10.7172	7.9912	-40.2811	7.2342	-54.619	6.1954
ligand042	-59.8028	-18.4623	1.8793	-38.5688	4.3004	-45.2187	4.3525	-44.4901	4.3157
ligand043	-67.3992	-20.0825	2.2303	-37.3053	5.4458	-45.985	5.2793	-46.0726	5.2756
ligand044	-78.4218	-17.2964	2.2732	-27.9102	5.8596	-51.3865	7.0684	-56.5368	6.6957
ligand045	-87.6807	-23.1379	2.6033	-53.5875	4.5808	-60.9932	4.8899	-59.8595	4.6343
ligand046	-85.9399	-20.0761	3.0666	-32.5671	5.3701	-56.5264	5.7155	-64.2568	5.7297
ligand047	-64.7174	-15.4593	2.2339	-38.3939	4.8768	-49.5439	5.5979	-49.6116	5.231
ligand048	-76.0615	-16.3126	2.211	-58.3798	7.0352	-65.3079	6.2494	-62.0689	5.8943
ligand049	-83.3634	-18.0639	3.4808	-42.0594	6.8409	-61.9819	6.4817	-66.3983	6.5711
ligand050	-65.4325	-17.7329	2.1141	-24.8396	3.6349	-30.4532	3.7924	-30.3697	3.7347
ligand051	-81.9793	-8.8107	3.3829	-24.7577	10.1568	-49.8248	7.2233	-58.0142	5.9937
ligand052	-65.3774	-35.3989	2.5201	-48.5615	3.8777	-50.339	3.9644	-49.8638	3.9195
ligand053	-80.5658	-23.9034	3.9454	-41.3435	5.7586	-49.6053	5.0999	-49.2042	4.8462
ligand054	-76.4759	-12.9716	2.6724	-27.9374	8.1414	-53.3449	7.5468	-60.8836	6.401
ligand055	-82.3823	-17.8391	2.5925	-30.0138	4.1753	-55.6083	4.6691	-64.7098	5.2322
ligand056	-78.5623	-19.6254	3.0458	-44.4997	6.8934	-51.0732	6.9562	-50.2452	6.7149
ligand057	-75.3924	-24.5348	2.8673	-49.3885	5.4968	-57.0191	6.0683	-55.9724	5.9685
ligand058	-66.0557	-16.0076	2.3814	-17.6481	6.334	-21.9835	6.189	-21.9956	6.2902
ligand059	-61.9034	-7.674	1.7998	-19.5014	4.3732	-29.1693	4.816	-28.9579	4.5788
ligand060	-70.9228	-22.44	1.9876	-35.4588	5.4642	-61.7499	5.6814	-70.6112	5.6745
ligand061	-80.3016	-19.7122	2.3876	-34.0865	5.8896	-38.8027	6.0313	-37.8202	6.3285
ligand062	-80.1137	-12.4737	3.0543	-23.7625	8.2993	-35.5053	8.497	-42.7735	8.6611
ligand063	-80.0282	-16.4716	2.1885	-31.3604	5.2065	-52.3737	5.1457	-55.6194	5.1442
ligand064	-76.69	-8.6403	2.0914	-31.2025	5.4207	-57.0011	5.5124	-66.0138	5.5282
ligand065	-84.6903	-12.5478	2.1669	-37.7792	5.6184	-60.1444	6.5679	-65.6239	6.3329
ligand066	-63.9172	-2.2814	3.2736	-3.8459	8.5021	-22.6243	8.8677	-27.629	10.7829
ligand067	-85.1407	-12.9352	2.8442	-26.9605	6.7996	-54.3503	6.6182	-64.3633	6.6415
ligand068	-77.5486	-10.1431	4.3283	-23.3275	10.159	-46.0265	7.0597	-52.0543	6.1206
ligand069	-76.4347	-18.2365	4.3939	-40.5557	8.2837	-51.6863	7.1962	-51.7846	6.6768
ligand070	-81.0141	-18.0631	3.2378	-33.9491	7.6172	-55.5339	6.448	-62.6616	6.64
ligand071	-79.7143	-7.0201	1.4271	-36.9854	5.9392	-60.3852	6.6016	-63.3807	6.2384
ligand072	-79.6122	-15.3551	3.8321	-31.798	7.3192	-47.7902	6.3473	-48.8494	6.0677
ligand073	-86.3879	-20.5102	2.3899	-39.6359	5.1618	-65.5774	5.4476	-72.0192	4.8266
ligand074	-76.5506	-21.0447	3.3217	-46.2898	5.2549	-52.9453	5.7678	-51.9813	5.5898
ligand075	-88.6695	-34.6829	2.7684	-43.8029	5.2939	-64.7811	6.1658	-70.9574	5.9608
ligand076	-80.213	-12.4571	2.0881	-35.5557	4.0329	-56.5618	5.5791	-62.7194	5.5227

ligand077	-85.5187	-16.9905	2.9692	-25.7445	6.8067	-52.4561	6.8299	-62.4542	6.3404
ligand078	-86.2436	-22.7639	3.3355	-36.1062	8.9524	-61.8366	7.0361	-70.6602	5.7378
ligand079	-82.2538	-17.6786	2.3446	-44.8061	5.4118	-59.4773	4.8591	-60.3025	4.7826
ligand080	-82.907	-19.7019	2.1942	-30.7882	5.3414	-57.0559	6.1053	-66.1387	5.7459
ligand081	-72.7086	-8.3678	2.1493	-16.2522	5.2225	-41.2793	6.5141	-50.6091	6.6216
ligand082	-83.5276	-21.8981	3.0503	-44.2809	5.816	-63.095	5.8519	-65.8324	5.6961
ligand083	-87.1491	-11.5224	4.7446	-19.3781	9.1431	-51.232	8.8608	-69.5191	7.415
ligand084	-68.3562	-20.1321	1.9263	-36.5847	4.8822	-49.3321	4.7854	-50.4154	4.69
ligand085	-84.4718	-18.5808	3.0823	-28.4504	6.0227	-58.6266	6.032	-71.0228	6.059
ligand086	-82.5432	-19.0454	3.7363	-38.8044	7.2078	-62.8619	6.9867	-71.4379	6.7395
ligand087	-83.354	-16.2115	3.1638	-28.3765	6.0673	-53.1287	6.1845	-57.4479	6.1442
ligand088	-79.9494	-16.571	2.6315	-33.8348	5.5078	-53.4933	5.5521	-57.462	5.394
ligand089	-66.4971	-16.6927	2.4018	-28.9907	4.04	-45.3132	5.4096	-48.6503	5.2368
ligand090	-82.9494	-22.655	2.4041	-42.4564	5.355	-63.2838	5.7272	-68.1936	5.1565
ligand091	-82.4893	-13.0205	2.47	-35.6218	5.5068	-52.661	5.1144	-58.0612	5.1743
ligand092	-82.2442	-17.9086	2.9474	-30.5403	6.1095	-55.1038	5.4276	-62.5091	5.1217
ligand093	-84.3961	-20.9723	3.6783	-38.1594	6.3033	-57.7235	5.9744	-62.0776	5.8839
ligand094	-80.93	-9.2792	3.8928	-20.0987	8.4156	-52.3323	8.0872	-67.7386	6.8928
ligand095	-87.1825	-4.8414	3.2809	-4.8522	7.7278	-36.7329	9.9477	-59.3312	13.8793
ligand096	-80.9316	-10.2437	1.9524	-21.387	4.7956	-51.1645	5.8371	-65.9551	6.2056
ligand097	-85.1169	-21.288	2.2931	-31.9072	4.8	-55.9618	5.6582	-63.7756	5.7094
ligand098	-74.6567	-7.3935	1.5631	-22.8501	5.8822	-51.9095	6.1884	-63.8433	6.0702
ligand099	-84.713	-15.9757	4.2257	-34.9402	5.1317	-57.2401	5.6985	-63.8415	5.959
ligand100	-85.1886	-8.4963	3.0276	-14.6308	6.2594	-47.6482	6.4364	-67.5464	6.6901
ligand101	-73.0467	-17.8754	2.7657	-34.7918	5.9239	-49.2194	6.023	-51.5087	5.5222
ligand102	-84.518	-5.6305	2.7484	-10.3241	7.6046	-44.0061	8.5133	-65.3874	7.2265
ligand103	-80.4568	-24.5697	2.9595	-46.6315	5.1162	-61.7244	5.6405	-63.5791	5.3486
ligand104	-79.9853	-14.8368	3.105	-28.6011	5.7403	-51.8436	6.223	-60.2848	5.7574
ligand105	-81.6498	-23.8925	2.6177	-41.0397	5.7884	-66.0469	5.6515	-72.3874	5.729
ligand106	-72.686	-18.863	2.4656	-41.7871	5.5723	-60.9632	5.5701	-64.6729	5.1971
ligand107	-74.792	-13.8595	2.7475	-27.395	6.1497	-48.6382	6.4187	-54.5552	6.2831
ligand108	-78.6972	-33.5019	2.6908	-58.8626	4.6966	-73.3789	4.9729	-75.0422	4.8748
ligand109	-74.727	-23.5378	2.4414	-51.4493	5.1511	-65.293	5.7761	-65.684	6.0037
ligand110	-76.6817	-16.2197	2.7469	-35.4929	6.3335	-60.0215	6.2406	-66.9608	5.8329
ligand111	-84.5358	-16.6399	2.9078	-27.1227	4.8129	-55.3079	5.8054	-66.7008	6.0685
ligand112	-85.8043	-21.381	3.9205	-34.0971	7.4765	-61.8652	6.3901	-72.1618	6.8375
ligand113	-81.3142	-27.0539	2.3181	-45.228	4.4739	-62.2657	5.0952	-65.0308	4.9825
ligand114	-81.7906	-20.4966	2.628	-32.5706	4.7691	-58.9721	5.9877	-67.4163	6.2195
ligand115	-82.2553	-11.5615	2.3797	-30.8472	6.1405	-58.0346	6.0148	-65.9788	5.4411
ligand116	-80.2063	-17.6169	4.3897	-32.9526	6.3418	-57.9787	6.8161	-66.1752	7.0372

Table S49. Rac1 VS docking scores and Nwat-MMGBSA relative binding energies (kcal/mol) computed with Nwat = 0, 30, 60 and 100 (second repetition).

		Nwat=0		Nwat=30		Nwat=60		Nwat=100	
ligand	docking score	ΔG	St.Dev.	ΔG	St.Dev.	ΔG	St.Dev.	ΔG	St.Dev.
ligand001	-71.9322	-15.0326	2.3222	-30.2597	6.4555	-49.1025	6.1018	-52.2385	5.3757
ligand002	-68.5546	-18.9163	3.6003	-30.0576	6.3078	-47.2303	6.0987	-52.898	6.1428
ligand003	-73.6737	-23.8343	2.1787	-35.2004	3.737	-45.6537	4.3695	-46.5433	4.2059
ligand004	-67.0682	-10.8654	2.8266	-21.2069	8.3283	-43.4973	7.0247	-50.9764	5.7763
ligand005	-75.0559	-16.3203	2.3126	-31.8823	4.6817	-45.8841	5.2763	-47.9215	5.2191
ligand006	-82.2897	-1.9392	2.7091	1.5749	9.4359	-32.0914	9.1088	-58.2304	7.7801
ligand007	-85.0969	-11.754	3.0182	-25.6862	7.363	-55.0152	7.8219	-67.2387	6.6025
ligand008	-83.3316	-22.5206	2.6246	-39.3275	5.8254	-55.6215	6.4901	-57.2018	6.4279
ligand009	-77.8068	-14.8782	5.3336	-32.7486	12.0523	-50.2617	6.3761	-53.1795	6.653
ligand010	-62.2758	-13.0872	2.5521	-20.7678	5.2488	-38.5052	5.6946	-44.7402	5.5886
ligand011	-78.6115	-13.3851	2.256	-31.5908	5.3744	-49.1774	5.7833	-50.6219	5.8111
ligand012	-79.9309	-15.3878	2.2794	-33.2079	5.1617	-51.1775	6.0533	-52.3625	5.8641
ligand013	-75.4044	-25.3551	3.1751	-52.5168	6.2887	-57.5898	6.2302	-56.0857	5.9361
ligand014	-75.6156	-17.1264	4.3564	-34.5661	6.0115	-49.4857	5.1031	-52.878	5.1603
ligand015	-87.0753	-5.6623	2.6686	-25.1275	8.7824	-47.2845	8.4163	-52.2093	8.9219
ligand016	-80.5604	-18.2615	2.9329	-33.8603	5.9892	-53.8015	5.6672	-60.8379	5.6039
ligand017	-81.1617	-27.6803	5.4885	-61.1388	5.4895	-64.4113	4.6721	-61.6128	4.7949
ligand018	-77.9454	-9.4816	2.0155	-42.0257	6.5665	-68.5629	6.2557	-73.3353	6.1551
ligand019	-78.1496	-9.7313	3.9401	-22.901	7.3676	-43.022	6.3261	-48.5595	5.8599
ligand020	-76.9891	-24.0063	2.5641	-40.2221	5.7425	-58.3374	5.748	-61.5011	5.6091
ligand021	-77.5787	-11.9789	2.2333	-22.5702	5.6776	-46.1499	6.0265	-52.0801	6.0023
ligand022	-78.178	-20.6626	3.6457	-42.309	5.6966	-48.2577	5.382	-47.8544	5.308
ligand023	-76.9999	-16.0022	3.7117	-35.8899	8.4698	-46.3856	6.1164	-46.9747	5.7265
ligand024	-59.6886	-0.0641	1.4862	5.6507	7.2309	-24.1604	7.9338	-40.5636	12.4825
ligand025	-63.9452	-19.8324	3.1111	-37.8288	5.4218	-55.1285	5.6267	-57.8606	5.6171
ligand026	-72.3488	-12.3289	3.3383	-49.6844	5.2225	-63.4601	5.8865	-63.3837	5.653
ligand027	-78.2312	-19.9284	4.6565	-38.6592	7.615	-47.5723	5.4055	-48.3132	5.3181
ligand028	-79.5983	-18.2217	2.8935	-34.4725	5.2853	-53.0888	5.8118	-56.5034	5.8737
ligand029	-79.8273	-20.4498	3.3475	-44.4186	6.863	-52.1655	6.7333	-51.7163	6.7455
ligand030	-60.5127	-10.3786	2.0458	-40.2977	5.4424	-55.6276	5.8446	-54.9573	5.5609
ligand031	-67.4659	-14.4139	2.2753	-37.4256	4.7644	-44.1027	4.7432	-43.4164	4.7218
ligand032	-79.2082	-9.9946	3.7198	-16.8689	9.7307	-48.1526	8.3515	-64.0299	6.3783
ligand033	-83.6017	-20.8901	2.7756	-33.5316	6.0014	-56.9073	6.4388	-64.2956	6.4244
ligand034	-78.0663	-18.4555	2.7401	-35.2987	5.2576	-51.1445	6.1244	-53.2767	6.1831
ligand035	-74.755	-18.7763	2.5347	-49.4744	5.7858	-57.7868	5.7773	-57.4659	5.6761

ligand036	-67.1964	-17.7967	2.1291	-33.6478	6.2941	-60.5621	6.9248	-70.2359	6.8248
ligand037	-79.4238	-18.5535	2.4638	-23.9346	5.677	-55.2616	5.9162	-70.7494	5.9468
ligand038	-82.9556	-13.5696	1.8464	-29.7812	5.1121	-47.6705	5.7575	-52.1525	5.7792
ligand039	-72.4193	-17.5356	2.5066	-27.344	5.4882	-53.6856	5.3338	-64.9512	5.3738
ligand040	-72.6611	-23.6418	2.7814	-43.0047	4.121	-46.9032	4.1964	-46.1341	4.0906
ligand041	-70.3186	-23.4299	2.8576	-45.74	5.9143	-52.9178	5.9606	-51.8977	5.9332
ligand042	-59.7994	-17.7246	2.1429	-36.8812	4.3399	-43.8939	4.3741	-43.2932	4.2302
ligand043	-69.5319	-11.55	2.251	-24.3308	5.7598	-44.4278	5.4948	-48.8253	5.4422
ligand044	-78.4474	-14.8181	2.2893	-30.7898	5.321	-49.2906	5.178	-51.1898	5.1613
ligand045	-87.5721	-22.0534	2.624	-54.2267	5.3721	-63.7324	5.1274	-62.8144	5.0677
ligand046	-86.0692	-24.7765	2.7622	-45.4355	5.1662	-66.9777	5.9022	-71.9311	6.2818
ligand047	-63.1531	-14.9356	2.5971	-36.7657	5.3314	-48.1249	5.4927	-48.0623	5.4074
ligand048	-76.805	-10.777	1.71	-48.776	6.2667	-65.0656	6.0918	-64.0139	5.7808
ligand049	-83.3338	-22.6218	4.5785	-40.5445	7.1213	-59.0342	6.7169	-62.5105	5.878
ligand050	-65.4171	-17.819	2.3364	-25.1851	3.3387	-30.7749	3.6662	-30.6179	3.5623
ligand051	-81.8759	-8.238	2.0476	-18.6065	7.2203	-43.7749	7.0961	-51.6269	6.9335
ligand052	-65.4182	-35.0117	2.9488	-47.8685	4.1117	-50.4912	4.212	-49.8793	4.0441
ligand053	-79.5331	-23.2892	2.6282	-40.8366	4.7059	-50.6339	5.337	-51.0567	5.0451
ligand054	-75.8879	-31.7325	2.9338	-52.9504	4.8205	-59.6802	4.9287	-58.6698	5.0183
ligand055	-82.3813	-19.5223	2.0414	-33.663	5.0776	-56.6963	5.3439	-63.5517	5.4794
ligand056	-78.6128	-13.9868	2.7081	-32.9252	5.6971	-49.1799	6.3067	-51.6203	6.3303
ligand057	-75.6098	-24.7005	2.9292	-50.3666	5.2348	-57.7883	5.5222	-56.9241	5.481
ligand058	-70.7339	-18.3746	2.2056	-15.1295	4.8191	-21.0627	5.0638	-21.6693	5.1021
ligand059	-61.8931	-13.9479	2.3501	-26.6166	3.9484	-30.1869	3.7456	-28.8039	3.4866
ligand060	-70.964	-22.7008	1.9176	-34.9957	4.8172	-61.2113	5.7683	-70.9429	5.6761
ligand061	-86.577	-23.8956	2.2683	-41.0254	5.2915	-56.5733	5.3997	-57.3192	5.4522
ligand062	-81.5952	-15.4425	2.2562	-26.4967	7.3091	-49.8185	6.4564	-57.3725	6.2805
ligand063	-79.9879	-17.7579	2.5904	-33.764	5.5107	-53.4873	6.1698	-57.3867	6.3714
ligand064	-76.6876	0.0072	0.0161	-15.0444	7.4726	-34.1808	12.7844	-39.3638	16.646
ligand065	-84.443	-10.3887	3.0057	-27.1722	6.4272	-57.419	7.3034	-67.5169	7.2247
ligand066	-64.0004	-12.7588	2.4077	-30.793	4.2483	-39.491	4.4233	-38.7284	4.3449
ligand067	-85.3518	-22.851	2.7182	-36.6661	6.1468	-61.5149	5.6945	-70.5101	6.1478
ligand068	-76.4181	-6.5873	3.2371	-20.0811	6.6785	-43.8711	6.6671	-49.4796	6.2507
ligand069	-77.5264	-6.6955	4.0647	-15.7892	7.3641	-42.0689	6.1644	-49.7959	5.5845
ligand070	-80.9963	-19.7821	2.5796	-39.3528	6.4742	-55.985	6.7014	-60.0271	6.1644
ligand071	-86.8569	-15.2689	2.0818	-52.2663	6.2516	-69.2299	5.8085	-69.0699	5.8633
ligand072	-79.6695	-16.7766	2.4083	-32.5509	5.7767	-47.0361	5.8549	-47.3515	5.5828
ligand073	-86.3542	-19.8194	2.8981	-28.3235	6.2796	-58.9141	6.6895	-72.4128	6.3456
ligand074	-76.5747	-13.8516	2.4624	-34.454	5.182	-51.3952	5.7894	-52.8701	5.7043
ligand075	-92.0141	-24.9819	2.6848	-30.4594	6.2977	-57.18	6.3009	-67.8044	6.2168

ligand076	-80.313	-22.1578	2.3488	-43.6429	5.4574	-62.7023	6.2354	-66.8322	5.9057
ligand077	-85.828	-23.0428	3.5141	-37.0994	5.9836	-58.847	6.7426	-64.7498	6.871
ligand078	-86.3394	-17.309	4.31	-28.1237	6.8273	-54.7876	6.4477	-64.8839	6.4433
ligand079	-82.3065	-12.6833	3.1318	-32.7643	6.9316	-55.8393	6.5769	-61.3812	6.3936
ligand080	-82.8357	-19.8749	2.4592	-34.4049	5.5125	-59.9736	6.2109	-67.9591	6.1029
ligand081	-72.5936	-10.5844	2.6478	-26.0327	6.0187	-47.0422	6.4881	-53.7806	6.4743
ligand082	-83.3447	-16.127	4.6562	-37.0322	6.3362	-58.9243	6.0806	-64.0199	5.613
ligand083	-86.9932	-24.3581	2.1337	-43.1941	4.8019	-66.1605	5.3199	-71.948	5.6274
ligand084	-68.3478	-18.587	1.9019	-33.142	4.4922	-48.297	5.0759	-51.1278	4.75
ligand085	-84.6206	-21.3382	3.4991	-33.2036	6.1181	-58.5105	5.8617	-66.715	6.1325
ligand086	-82.6194	-20.1397	2.137	-31.852	5.0817	-57.2756	5.9577	-66.1608	6.2663
ligand087	-76.341	-17.3144	2.4061	-34.5311	5.1594	-53.5819	5.6585	-57.1872	5.7866
ligand088	-79.9222	-15.8599	2.6234	-34.9414	4.8247	-55.5782	5.5751	-58.8993	5.5579
ligand089	-69.9927	-6.3442	2.0805	-9.922	5.476	-34.2691	5.9566	-43.8882	6.235
ligand090	-83.0355	-19.8539	4.0146	-32.079	6.0157	-60.9237	6.2303	-72.5354	5.848
ligand091	-82.371	-11.9711	2.8463	-22.6651	5.5921	-48.2084	5.7104	-61.2082	6.4157
ligand092	-82.2442	-17.6885	3.1784	-27.3423	6.451	-58.0317	6.7358	-72.3807	7.1136
ligand093	-84.4685	-23.1054	3.0568	-44.8101	5.9826	-62.8786	5.6603	-66.3843	5.1516
ligand094	-80.8895	-8.6034	3.3386	-15.3471	8.3029	-46.262	7.8501	-65.011	8.1763
ligand095	-87.1492	-20.7597	4.063	-36.7515	7.265	-61.6504	7.5989	-69.4028	7.3934
ligand096	-80.9111	-18.2057	1.9148	-30.5269	4.8681	-53.419	5.7195	-59.662	6.0207
ligand097	-85.4636	-15.8887	3.4289	-32.772	6.5793	-61.0028	6.817	-70.9866	6.7329
ligand098	-74.7174	-4.198	2.3627	-15.8086	7.0646	-45.4477	5.8645	-61.5597	5.8244
ligand099	-84.762	-21.7359	3.7688	-34.2855	6.5189	-61.2619	5.9332	-69.3077	5.6314
ligand100	-85.1578	-16.7638	5.1844	-26.2968	7.1353	-57.1865	6.721	-71.4931	6.8926
ligand101	-73.0664	-20.9238	3.2862	-36.0391	6.085	-51.4583	5.853	-55.0031	5.725
ligand102	-84.2518	-18.7434	2.5354	-43.6682	5.6538	-62.9308	5.236	-67.3447	5.0781
ligand103	-80.2426	-21.5851	2.0701	-44.2868	4.9137	-60.6321	5.3673	-63.1258	5.475
ligand104	-80.1219	-13.5356	2.8664	-22.846	7.0693	-49.916	6.7812	-61.5265	5.9689
ligand105	-81.8418	-23.4436	3.117	-35.0458	6.0934	-61.8025	7.5058	-70.923	7.6539
ligand106	-71.8352	-14.972	2.4996	-24.6162	5.4058	-50.4268	5.7759	-58.925	5.6792
ligand107	-75.1139	-16.2991	3.4494	-40.0664	6.5667	-55.544	6.2666	-57.3268	6.5452
ligand108	-78.882	-34.7452	3.2362	-60.7638	5.4911	-73.7405	5.719	-75.5476	5.579
ligand109	-74.7711	-13.8472	5.1593	-34.0144	9.7196	-57.5906	7.9753	-62.9976	6.5391
ligand110	-77.4082	-21.3521	4.0465	-38.3368	6.1898	-61.0979	6.6205	-66.9008	6.0391
ligand111	-84.4923	-11.8735	5.0382	-27.6823	6.4689	-55.0436	6.0845	-64.0519	5.7262
ligand112	-85.7402	-23.98	2.8534	-36.761	6.48	-60.8681	5.9286	-67.6814	6.4864
ligand113	-81.4953	-19.598	2.3998	-33.009	5.1344	-54.379	5.7234	-61.1657	5.3397
ligand114	-81.6305	-13.3197	3.0984	-23.3112	5.8417	-51.3172	6.5269	-64.4208	7.1139
ligand115	-82.2377	-18.1083	3.2334	-29.3823	7.9517	-57.5593	7.6455	-67.6521	6.8277

ligand116	-80.4904	-13.3133	3.3869	-25.4243	5.2418	-55.6651	5.6155	-66.6393	6.0124

Table S50. Rac1 VS docking scores and Nwat-MMGBSA relative binding energies (kcal/mol) computed with Nwat = 0, 30, 60 and 100 (third repetition).

		Nwat=0		Nwat=30		Nwat=60		Nwat=100	
ligand	docking score	ΔG	St.Dev.	ΔG	St.Dev.	ΔG	St.Dev.	ΔG	St.Dev.
ligand001	-71.7415	-8.9597	4.2941	-16.1394	8.0944	-39.6799	7.7775	-47.1612	7.8716
ligand002	-68.3504	-18.0727	3.0762	-29.6498	5.4539	-47.3767	5.6246	-53.0195	5.9917
ligand003	-73.6619	-21.0608	2.9324	-33.241	4.668	-46.3265	4.3314	-47.4072	3.9023
ligand004	-67.1401	-1.1	2.0422	3.7685	7.159	-24.8492	7.9823	-43.0715	9.5557
ligand005	-75.0377	-15.5798	3.1368	-28.6243	6.0195	-43.4172	5.4957	-46.909	5.4852
ligand006	-82.2155	-23.9284	3.3094	-41.7877	5.9949	-62.3903	6.3709	-66.9694	6.2368
ligand007	-85.1276	-17.6635	2.6691	-37.7675	5.7928	-61.0519	5.8101	-67.9973	5.9051
ligand008	-83.3385	-21.3834	2.2345	-37.2027	5.668	-55.1527	5.9495	-57.4526	5.801
ligand009	-77.0482	-23.1132	3.1398	-50.0378	6.1607	-56.7317	6.1235	-54.5642	5.8811
ligand010	-62.3067	-24.0568	2.1231	-34.9979	4.399	-44.5694	4.825	-45.5734	4.6463
ligand011	-78.6126	-13.0604	2.6356	-31.8954	6.3345	-49.0375	6.5183	-51.8008	6.3418
ligand012	-77.2884	-19.9707	3.1023	-37.7755	6.02	-50.1298	6.1391	-50.4992	6.0101
ligand013	-77.1379	-18.0764	2.8406	-44.1464	4.8893	-54.2691	5.2176	-53.0218	5.1765
ligand014	-75.968	-14.9286	2.3358	-31.3885	5.1222	-50.4862	5.5367	-57.0378	5.4694
ligand015	-86.5134	-14.0501	2.9536	-47.7161	6.006	-60.8423	5.8755	-59.6143	5.7922
ligand016	-80.4469	-15.2784	3.8596	-30.8317	6.9942	-52.2425	6.4916	-59.8436	6.502
ligand017	-82.9034	-6.8092	1.9518	-46.3841	5.148	-66.1214	5.4696	-65.7654	5.8328
ligand018	-81.132	-17.9786	2.4917	-56.0412	6.8613	-71.3911	6.9409	-70.8831	6.6794
ligand019	-78.2265	-1.4743	3.2418	-2.6604	9.1212	-32.9841	7.8859	-47.491	7.3226
ligand020	-77.1055	-22.5613	2.0588	-35.8474	4.7389	-54.6915	5.1818	-60.3394	5.4334
ligand021	-77.5167	-8.0712	2.5853	-18.4839	6.4526	-45.5201	6.1745	-51.3988	6.2412
ligand022	-78.2031	-11.6514	2.7243	-26.3898	6.0532	-45.1497	6.1367	-49.7602	6.4578
ligand023	-76.9101	-14.9178	3.0124	-32.2291	5.8193	-46.279	6.2269	-47.6093	6.0136
ligand024	-71.9083	-26.4521	3.256	-49.317	5.1378	-55.949	5.1644	-55.007	5.1064
ligand025	-72.0578	-27.8614	2.773	-50.6684	5.8284	-60.528	5.7403	-60.5924	5.6684
ligand026	-72.1865	-8.9018	2.4691	-32.5614	6.2479	-57.9546	5.5916	-66.8869	5.4358
ligand027	-78.2589	-12.717	3.1766	-27.6948	7.1285	-44.6504	6.1227	-47.1641	6.1342
ligand028	-79.5939	-15.0873	2.5918	-32.1843	4.9536	-52.4098	6.0449	-56.2016	6.1705
ligand029	-80.2073	-12.271	3.0231	-27.6233	7.1227	-47.4244	5.9055	-50.181	5.2467
ligand030	-61.6397	-4.4683	1.2462	-30.736	5.0102	-56.0166	6.694	-60.5344	7.1577
ligand031	-67.4716	-15.936	1.8894	-37.8163	3.6766	-43.5372	4.0326	-42.7265	3.9036
ligand032	-78.4875	-20.9583	3.5252	-33.7171	6.8041	-57.0038	7.39	-65.1688	7.282
ligand033	-84.3289	-14.7598	4.4773	-30.0496	6.3858	-56.6953	5.9752	-66.4245	6.0314
ligand034	-76.474	-11.6813	4.3066	-24.3021	9.6176	-44.435	5.6727	-49.104	5.33

ligand035	-74.4373	-18.7526	2.2608	-48.3604	6.671	-58.9892	6.4629	-59.3404	6.561
ligand036	-67.1474	-17.5935	2.628	-32.2659	6.1594	-59.5834	6.7101	-69.1457	6.3503
ligand037	-80.3484	-11.2677	5.7063	-18.5178	10.5996	-47.1588	8.2834	-61.2276	5.8542
ligand038	-87.571	-22.7392	2.2037	-34.0836	4.8957	-54.3334	6.3468	-60.2035	6.3187
ligand039	-74.014	-21.099	2.8226	-26.9292	5.6922	-50.1308	6.6337	-59.7089	6.1661
ligand040	-72.2103	-12.5675	1.9555	-37.3565	4.3623	-47.2846	4.1514	-46.6746	3.9917
ligand041	-73.5971	-15.4377	2.3532	-39.0028	5.9063	-51.0541	6.2003	-51.1056	5.6677
ligand042	-70.2934	-21.8307	2.0646	-37.8831	3.8087	-44.6641	3.8185	-44.3806	3.7182
ligand043	-72.2481	-16.5164	2.2226	-32.0793	5.0104	-46.1272	4.8767	-47.4223	4.7374
ligand044	-78.3889	-14.9782	3.4209	-33.3173	5.1087	-51.2838	5.4342	-54.0254	5.4861
ligand045	-87.6346	-21.1451	2.3555	-52.4157	4.1705	-59.9367	4.3593	-58.8322	4.3649
ligand046	-86.0615	-19.1707	5.8139	-30.6957	8.9467	-60.4015	7.4687	-73.6268	7.2573
ligand047	-64.817	-9.2363	4.4264	-24.2564	8.6472	-43.3006	6.3491	-45.2954	5.6136
ligand048	-77.3175	-4.2591	3.5992	-36.1452	9.6042	-61.1527	6.7145	-65.1085	6.8567
ligand049	-83.3649	-16.0614	4.1187	-39.9374	7.4519	-61.4344	6.9496	-66.1316	6.3124
ligand050	-65.4188	-17.0274	2.3836	-24.5933	3.8187	-30.3683	3.5697	-30.1759	3.5237
ligand051	-79.9095	-13.2998	2.2407	-36.1002	6.3388	-59.9616	5.5748	-63.2255	4.8323
ligand052	-65.3917	-33.1874	2.5987	-47.5095	3.9828	-50.0762	4.0695	-49.5284	3.934
ligand053	-82.0577	-23.0357	2.6494	-39.4673	4.523	-47.5157	4.6463	-47.1575	4.4936
ligand054	-76.3067	-11.6952	3.6132	-20.0949	7.854	-48.1909	6.702	-61.1169	6.0904
ligand055	-82.6167	-19.0614	2.621	-30.8133	6.2637	-55.5048	5.9203	-63.6306	6.0858
ligand056	-78.6233	-13.3021	2.4632	-31.6668	5.2781	-49.089	5.8396	-51.8226	5.8137
ligand057	-75.6454	-23.3731	3.1769	-48.3949	4.8825	-56.1346	5.0249	-55.2695	5.067
ligand058	-67.1024	-13.5949	2.4301	-12.6192	4.637	-22.7644	5.0508	-24.5736	5.2809
ligand059	-61.8712	-8.2381	1.7701	-24.4865	4.793	-30.7715	4.165	-29.5806	4.0286
ligand060	-67.5879	-22.3401	1.8789	-34.8026	5.0124	-61.3768	5.5549	-70.176	5.4966
ligand061	-83.6679	-11.4654	2.9506	-21.097	7.0577	-38.2263	6.9176	-47.0625	7.8914
ligand062	-86.5678	-13.5649	2.2764	-27.6922	7.5614	-53.6719	8.5043	-63.9882	7.6221
ligand063	-72.438	-16.4372	3.0769	-30.8835	6.7913	-52.8548	6.696	-57.8153	6.4465
ligand064	-76.6989	-10.5695	2.0098	-35.2464	4.8025	-58.0488	5.3445	-64.6874	5.2694
ligand065	-84.3284	-12.2971	2.1537	-27.7829	5.6402	-55.8435	6.26	-66.0584	6.5115
ligand066	-63.9548	-12.891	2.1068	-26.4968	4.8794	-36.7779	5.0124	-37.2752	4.9091
ligand067	-85.3346	-13.2715	3.1695	-25.0109	6.1654	-55.3131	7.0341	-68.6551	7.11
ligand068	-76.3942	-12.4757	2.2277	-28.2712	5.4883	-47.4993	5.9598	-50.1545	5.8539
ligand069	-77.5062	-13.8203	2.3614	-29.2319	6.6741	-47.1787	5.9795	-49.0909	5.743
ligand070	-81.1354	-20.0435	2.7794	-39.2552	8.0966	-55.4208	7.5381	-59.0677	6.7334
ligand071	-83.6051	-6.9547	3.7756	-39.2183	12.881	-58.4649	6.1745	-60.9912	5.8508
ligand072	-79.8882	-15.2813	2.7898	-34.119	5.1959	-48.7148	5.6857	-49.629	5.6744
ligand073	-86.4123	-22.3669	3.0557	-32.5953	5.2729	-62.5365	6.92	-75.4655	7.0247
ligand074	-78.0962	-24.1079	3.0765	-48.7732	5.3557	-54.4206	5.2502	-53.173	5.264

ligand075	-91.653	-26.2028	7.2432	-30.0434	11.7156	-58.011	8.2042	-69.4469	7.0936
ligand076	-80.2663	-15.0024	3.6866	-35.7489	5.7856	-58.6506	6.7928	-65.0504	6.8109
ligand077	-85.7166	-20.4936	2.5887	-36.5691	4.7167	-60.2905	5.4745	-66.9769	5.8428
ligand078	-86.1872	-20.5097	3.279	-32.1342	7.8324	-61.5804	6.7819	-73.6826	6.7519
ligand079	-81.3117	-5.437	2.2392	-13.1098	7.145	-44.6841	7.6663	-60.5342	9.6057
ligand080	-82.863	-13.8215	2.3675	-29.7919	6.7601	-59.8118	6.7471	-71.7966	6.9413
ligand081	-78.9824	-17.224	2.4577	-34.8348	5.1793	-55.7637	5.384	-59.796	5.2344
ligand082	-83.3551	-19.3592	2.4404	-38.4278	5.8726	-62.3802	6.1574	-68.3365	5.8783
ligand083	-87.1379	-22.4179	2.4039	-30.7084	5.0465	-60.7997	6.3091	-75.4862	6.593
ligand084	-68.3554	-18.328	1.8034	-32.3393	3.5715	-46.5055	4.3497	-49.0976	4.3239
ligand085	-84.5862	-22.2125	3.2967	-37.8588	6.9177	-59.9415	5.7895	-65.4094	5.5246
ligand086	-82.6222	-17.9239	3.3439	-27.497	5.8456	-53.0532	5.5471	-63.4996	5.538
ligand087	-83.3434	-17.4068	2.5189	-35.3753	5.1797	-51.8431	6.0272	-52.8167	5.98
ligand088	-79.946	-19.3814	3.0267	-35.5588	5.0465	-56.1775	5.796	-60.6071	5.8855
ligand089	-68.0628	-3.6896	3.8709	-3.841	9.6933	-33.2458	8.4834	-49.352	6.4663
ligand090	-83.0351	-23.4173	5.8778	-42.6886	6.8342	-65.2124	6.7368	-71.1583	6.469
ligand091	-82.3608	-12.1768	3.8418	-22.0875	7.1169	-47.911	6.6035	-59.736	6.6515
ligand092	-82.2809	-22.2177	2.3959	-33.6584	5.6068	-62.2318	6.1455	-73.4483	6.494
ligand093	-84.6604	-20.3024	2.7666	-36.6295	6.4917	-56.8971	6.3057	-62.3678	6.3867
ligand094	-81.1758	-14.6654	3.9188	-23.9905	8.1579	-52.1435	6.5101	-64.3468	6.865
ligand095	-87.1433	-4.7822	4.9093	-2.6243	12.5217	-34.3236	12.2088	-59.7178	10.7184
ligand096	-80.8712	-16.8106	2.3667	-27.3488	6.1763	-53.33	5.6651	-62.572	5.8304
ligand097	-85.3986	-23.0717	2.0927	-41.6392	4.7273	-64.9851	5.3587	-71.7769	5.6952
ligand098	-78.811	-21.7729	2.2751	-45.0237	4.5406	-48.4358	4.6098	-46.8493	4.4854
ligand099	-84.8937	-21.4887	2.88	-38.0889	5.3476	-62.8041	5.9234	-70.1644	6.3934
ligand100	-85.2133	-9.9992	2.6211	-20.6194	7.426	-55.3326	6.9395	-73.3068	6.2016
ligand101	-73.02	-15.3151	2.5434	-26.0506	6.3296	-47.0776	6.5132	-54.6934	6.4068
ligand102	-84.2759	-9.014	2.169	-14.1963	6.9455	-49.0968	7.0388	-68.6318	7.4226
ligand103	-80.4194	-6.9455	3.1335	-24.6892	6.13	-51.1377	6.2311	-59.9371	6.195
ligand104	-79.525	-10.1548	4.1887	-15.7661	9.7685	-48.8748	9.6149	-65.0208	7.7302
ligand105	-81.7741	-22.429	2.2283	-32.0456	5.8213	-61.758	5.4108	-75.2277	5.6306
ligand106	-75.5161	-25.1191	2.874	-44.864	5.3537	-59.5198	5.3658	-61.8499	5.2445
ligand107	-76.4572	-28.9998	2.6696	-51.0571	4.7618	-57.7913	5.1657	-56.7699	5.2217
ligand108	-78.3836	-32.7342	2.5066	-60.5048	5.5197	-75.237	5.6384	-76.8255	5.4087
ligand109	-74.7477	-8.2279	2.3418	-21.038	4.9433	-50.559	5.5159	-62.2207	5.8269
ligand110	-78.9191	-18.4215	2.2363	-32.1737	4.6808	-57.2315	5.5382	-64.496	5.5084
ligand111	-84.4903	-14.4218	4.6003	-29.2163	6.2277	-53.5945	6.9768	-62.0004	7.4833
ligand112	-85.9739	-13.2437	2.212	-27.1276	6.5373	-55.8116	6.69	-67.222	6.837
ligand113	-81.508	-13.1772	2.3343	-36.6818	4.6899	-57.4034	5.4101	-62.6772	5.7731
ligand114	-81.8196	-21.785	3.4323	-36.4144	6.4024	-63.4856	6.6958	-71.9547	6.4151

ligand115	-82.2579	-18.2881	1.9181	-28.4524	4.7547	-53.8013	5.3744	-62.8049	5.6943
ligand116	-80.3855	-10.9686	3.3883	-26.9702	6.4298	-53.7378	6.5129	-62.839	6.7104
